# Single-Center Trifunctional Organocatalyst Enables Fast and Controlled Polymerization on *N*-Carboxyanhydride

**DOI:** 10.1021/acscentsci.4c01346

**Published:** 2024-10-29

**Authors:** Kang Chen, Yueming Wu, Minzhang Chen, Jiangzhou Wang, Min Zhou, Xin Chen, Runhui Liu

**Affiliations:** † State Key Laboratory of Bioreactor Engineering, East China University of Science and Technology, Shanghai 200237, China; ‡ Shanghai Frontiers Science Center of Optogenetic Techniques for Cell Metabolism, Frontiers Science Center for Materiobiology and Dynamic Chemistry, Engineering Research Center for Biomedical Materials of Ministry of Education, Key Laboratory of Specially Functional Polymeric Materials and Related Technology (Ministry of Education), School of Materials Science and Engineering, East China University of Science and Technology, Shanghai 200237, China; § Department of Biomaterials and Stem Cells, Suzhou Institute of Biomedical Engineering and Technology, Chinese Academy of Sciences, Suzhou 215163, China; ∥ Key Laboratory for Ultrafine Materials of Ministry of Education, School of Materials Science and Engineering, East China University of Science and Technology, Shanghai 200237, China

## Abstract

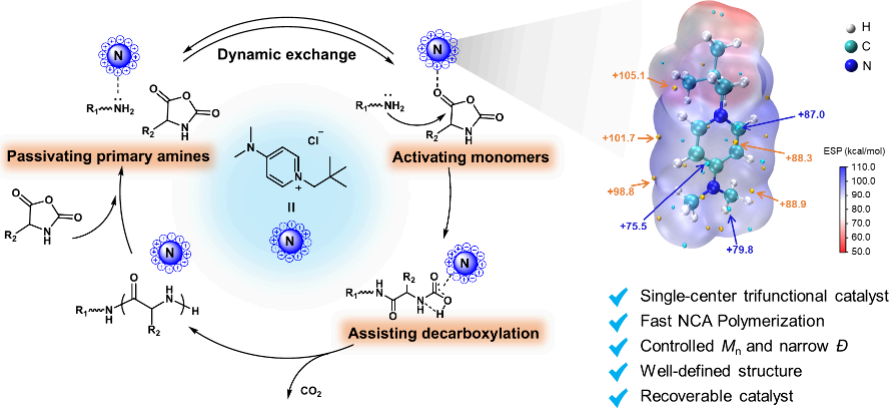

Ring-opening polymerization on *N*-carboxyanhydrides (NCA) initiated by primary amines has been the dominantly used method to prepare polypeptides with widespread applications. However, this polymerization chemistry suffers from slow polymerization rate, limited controllability, and difficulty in preparing high molecular weight polypeptides. Herein, we develop a conjugated cationic catalyst featuring cation–dipole interaction, which remarkably enhances the reaction rate and controllability of NCA polymerization, simultaneously, to afford polypeptides in a short time with predictable molecular weights (DP = 20–500) and narrow dispersities. Experimental data and computational study altogether indicate that conjugated cationic catalysts manifest a single center with triple functions by activating C5-carbonyl on NCAs to enhance the electrophilic activity of NCA monomer, activating carbamate intermediates to accelerate decarboxylation, and moderately passivating primary amines to improve controllability. Notably, this cationic-catalyst is well recyclable while keeping excellent catalytic performance. Thus, the highly efficient cationic-catalyst strategy implies practical and promising applications, representing a new avenue of catalyst design for polymerization chemistry.

## Abstract 2

*N*-Carboxyanhydride (NCA) polymerization initiated by primary amine is an convenient and extensively used chemistry to prepare polypeptides. However, this polymerization method suffers from slow polymerization, limited controllability, and difficulty in preparing high molecular weight polypeptides. We propose a new class of catalysts using an unprecedented mode, cation−dipole interaction, that completely overcomes these longstanding challenges in classical primary amine-initiated NCA polymerization.

## Introduction

Synthetic polypeptides, also known as poly(amino acids), have widespread applications in various fields such as antimicrobials,^1−10^ tissue engineering,^11−13^ and drug and gene delivery.^14,15^ Ring-opening polymerization on amino acid *N*-carboxyanhydrides (NCAs) is a classical method for the synthesis of polypeptides.^16−100^ Primary amines are the dominantly used initiators for NCA polymerization,^26−28^ with the advantages of easily accessible sources and diverse structures, enabling the synthesis of polypeptides with different topologies, such as linear and branched shapes.^29,30^ However, the classical primary amine-initiated NCA polymerization suffers from slow reaction rates and sensitivity to moisture without catalysts, hindering the facile synthesis of high molecular weight polypeptides.^17,31,32^ Moreover, the basicity of primary amines can deprotonate NCAs, leading to side reactions via the activated monomer mechanism.^33,34^ Catalysts can accelerate polymerization or improve polymerization controllability, such as 1,1,3,3-tetramethylguanidine and crown ether to accelerate the polymerization rate of primary amine-initiated NCA polymerization,^35−39^ and borane to passivate primary amines to improve the controllability of NCA polymerization.^40−42^ Although the aforementioned efforts have been devoted to NCA polymerization, concurrently achieving fast and precisely controlled NCA polymerization, initiated by primary amines, to synthesize high molecular weight polypeptides remains an obvious obstacle. For nonprimary amine initiators, catalysts such as *N*,*N*′-bis[3,5-bis(trifluoromethyl)phenyl]thiourea,^43^ fluorinated alcohol,^44^ and trithiourea catalyst^45^ have been investigated, where hydrogen-bonding interactions play a major role. However, inter- and intramolecular hydrogen bonds may exist between hydrogen-bonding catalysts, therefore possibly affecting their catalytic performance.^46,47^ Given the above challenges, there is an urgent need to explore a new catalytic strategy to simultaneously achieve fast and controlled NCA polymerization for the preparation of polypeptides using primary amines as the initiator, ideally without using hydrogen-bonding interactions.

Cation–dipole interactions constitute a crucial mode of interaction utilized in catalysis.^48,49^ Catalysts involved in this interaction mode are positively charged and repulsive to each other, minimizing the concern about inter- or intramolecular interferences. Notably, the structures of NCA monomers, carbamate intermediates, and primary amine reaction centers all possess polar groups with lone-pair electrons, which are carbonyl, carboxyl, and amino groups, respectively. Inspired by the cation–dipole interaction between cations and polar groups, we hypothesize that cations can interact with these three types of polar groups through a cation–dipole interaction, acting as an ideal trifunctional catalyst for NCA polymerization.

**Figure 1:**
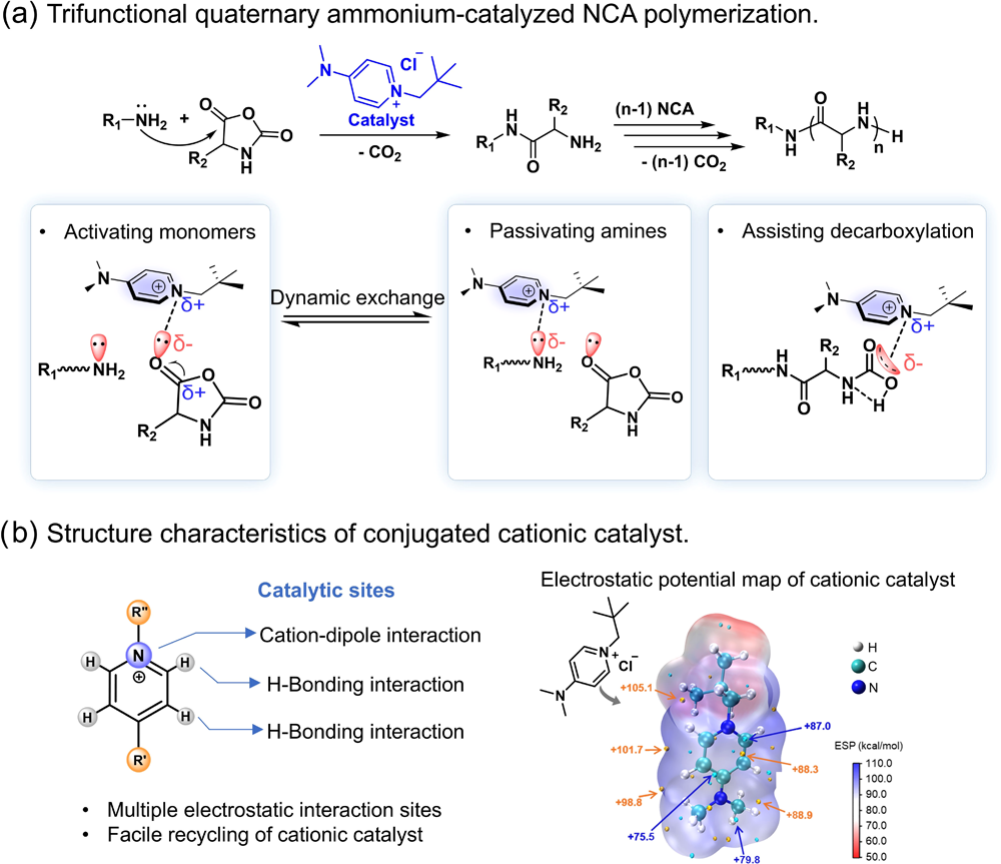
(a) Trifunctional quaternary ammonium-catalyzed ring opening polymerization (ROP) of NCAs using a primary amine as the initiator. (b) Structure characteristics of conjugated cationic catalyst. Electrostatic potential (ESP) map of the optimal quaternary ammonium salt catalyst. Significant surface local minima and maxima of ESP are represented as orange and cyan spheres and labeled by blue and orange texts, respectively.

In this study, we explore a new class of catalysts using an unprecedented mode, cation–dipole interaction, that simultaneously achieves fast and controlled NCA polymerization. The optimal cationic catalyst manifests a single center with triple functions: (1) activating C5-carbonyl on NCAs to enhance the electrophilic activity of the monomer and accelerate the polymerization, (2) activating carbamate intermediates to facilitate decarboxylation, and (3) moderately passivating the primary amine to diminish side reactions and improve controllability (Figure 1a). The unique design of cationic catalysts involves the conjugated structure that exhibits a delocalized positive charge throughout the structure. This feature enables cationic catalysts with multiple catalytic sites to interact with NCAs, carbamates, and primary amines through cation–dipole interaction. Moreover, the optimal cationic catalyst demonstrates good recyclability, retaining a high catalytic efficiency even after five recycling cycles (Figure 1b). In short, trifunctional cationic catalysts simultaneously increase the reaction rate and controllability of NCA polymerization to prepare polypeptides in a short time with predictable molecular weight (DP = 20–500) and narrow dispersity, which represents a new avenue of catalyst design for polymerization.

## Results and Discussion

To explore the function of the catalyst, we utilized primary amine-initiated γ-benzyl-l-glutamate *N*-carboxyanhydride (BLG) NCA polymerization (at a [M]:[I] molar ratio of 100:1) without catalyst as the control for comparison, which is slow, taking over 3 days to complete (Table 1, entry 1). We examined catalysts containing a variety of conjugated cations, including 5-butyl-7-methyl-1,5,7-triazabicyclo[4.4.0]dec-5-enium chloride (MTBDCl), 8-butyl-1,8-diazabicyclo[5.4.0]undec-7-enium chloride (DBUCl), 4-dimethylamino-1-neopentylpyridinium chloride (DMAPPCl), 1,3-bis(2,4,6-trimethylphenyl)imidazolium chloride (B(TMP)MCl), and 1,3-dimethylimidazolium chloride (DMMCl), for their efficacy in catalyzing the polymerization of BLG NCA. The result showed that DMAPPCl substantially increased the BLG NCA polymerization rate to complete within 2 h, superior to MTBDCl (6 h), DBUCl (5 h), B(TMP)MCl (3 h), and DMMCl (2.5 h) as the catalyst (Table 1, entries 2–6, GPC traces in Figure S1). The performance of the best catalyst, DMAPPCl, may be attributed to its larger conjugate structure compared to MTBDCl, DBUCl, and DMMCl, which enhances its interaction with the NCAs and the active centers for polymerization, whereas the excessively conjugated structure of B(TMP)MCl reduced the catalyst’s charge density and resulted in a poor catalytic performance (Figure S2). By exploring the molar ratio of DMAPPCl catalyst at 0.5%, 1%, 3%, 4%, 6%, and 8% relative to BLG NCA (Table 1, entries 6–11, GPC traces in Figure S1), we found ideal ring-opening polymerization (ROP) of BLG NCA using 4% of the catalyst to obtain poly(γ-benzyl-l-glutamate) (PBLG) in 70 min with the expected number-average molecular weight (*M*_n_) and a narrow dispersity (*M*_n_ = 22.2 kg/mol, *Đ* = 1.06, Table 1, entry 9, GPC trace in Figure S1). Notably, DMAPPCl does not initiate NCA polymerization (Table S1, entry 1). In addition, 4-dimethylamino-1-butylpyridinium chloride (DMAPBCl) could also catalyze controlled polymerization to complete the reaction in 1.5 h, slightly slower than DMAPPCl catalyzed polymerization, whereas DMAP hydrochloride was unable to catalyze the polymerization on NCA, indicating that alkylation on *N*-substituted quaternary ammonium salt was necessary (Table 1, entries 12 and 13, GPC traces in Figure S1).

**Table 1:**
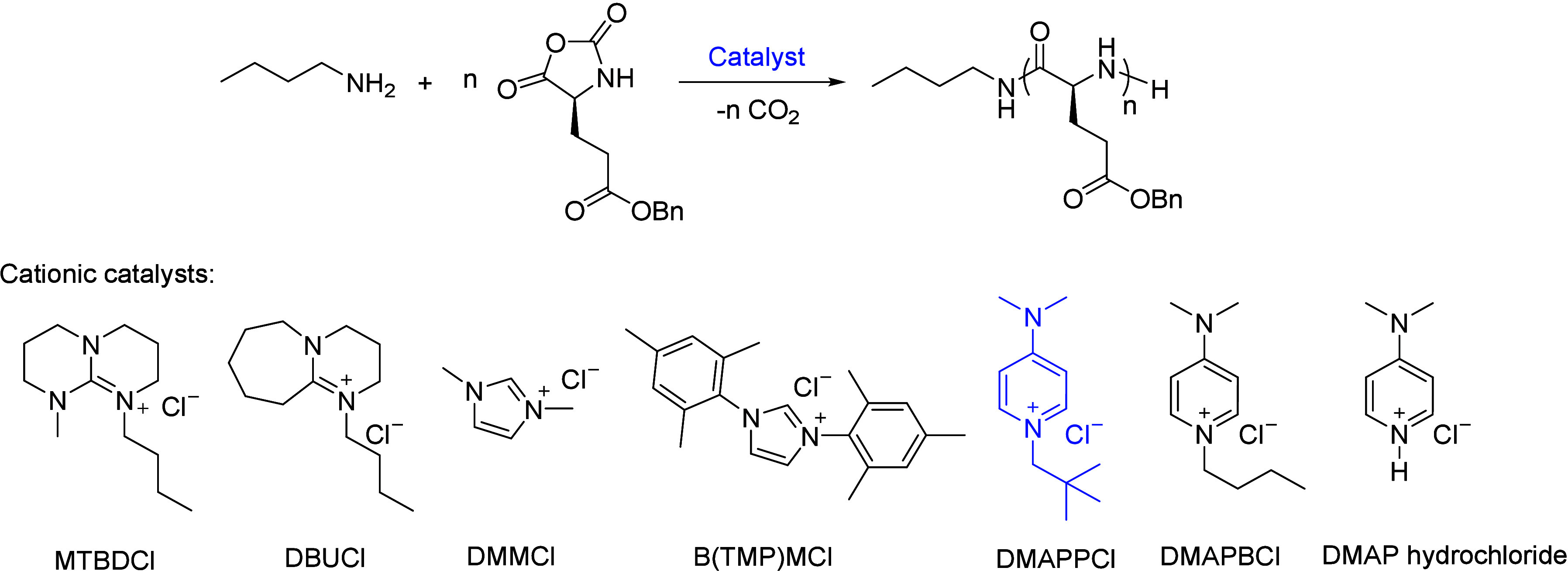
*N*-Butylamine-Initiated ROP of BLG NCA in the Presence of Different Catalysts^a^

entry	catalyst	[M]/[I]/[Cat.]	time	*M*_n,calcd_^b^ (kg/mol)	*M*_n,SEC_ (kg/mol)	*Đ*
1	none	100/1/0	3 d	22.0	28.0	1.16
2	MTBDCl	100/1/1	6 h	22.0	33.6	1.29
3	DBUCl	100/1/1	5 h	22.0	32.1	1.27
4	DMMCl	100/1/1	2.5 h	22.0	30.0	1.08
5	B(TMP)MCl	100/1/1	3 h	22.0	31.4	1.14
6	DMAPPCl	100/1/1	2 h	22.0	37.2	1.15
7	DMAPPCl	100/1/0.5	3.5 h	22.0	51.1	1.18
8	DMAPPCl	100/1/3	1.5 h	22.0	26.0	1.13
9	DMAPPCl	100/1/4	70 min	22.0	22.2	1.06
10	DMAPPCl	100/1/6	1 h	22.0	21.6	1.17
11	DMAPPCl	100/1/8	1 h	22.0	20.8	1.24
12	DMAPBCl	100/1/4	1.5 h	22.0	22.5	1.10
13	DMAP hydrochloride	100/1/4	>3 d	22.0	N/A^c^	N/A^c^
14	DMAPPCl	20/1/4	15 min	4.4	4.5	1.08
15	DMAPPCl	50/1/4	40 min	11.0	11.8	1.14
16	DMAPPCl	150/1/4	4 h	33.0	33.5	1.18
17	DMAPPCl	200/1/4	5 h	44.0	47.4	1.04
18	DMAPPCl	500/1/4	12 h	109.5	109.3	1.08

a All polypeptides were characterized by gel permeation chromatography (GPC) using 0.01 M LiBr in DMF as the mobile phase at a flow rate of 1 mL/min at 50 °C. *M*_n,SEC_ is the number-average molecular weight. *Đ* is the dispersity index. d*n*/d*c* (PBLG) = 0.104 mL/g; the value is consistent with the report in the literature.^21,36,44^

b *M*_n,calcd_ is the theoretical number-average molecular weight.

c N/A denotes that data were not collected because little reaction happened after 3 days according to TLC analysis.

**Figure 2:**
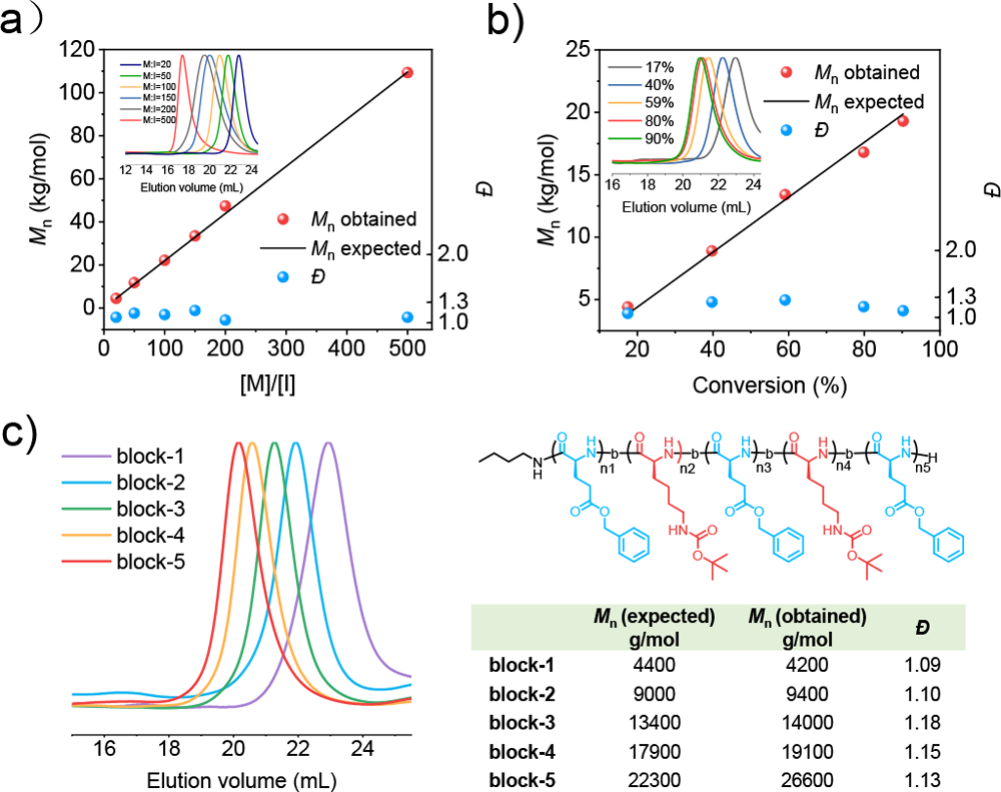
DMAPPCl-catalyzed controlled and living polymerization on NCA using *n*-butylamine as the initiator. (a) Plots of *M*_n_ and *Đ* against [M]:[I] ratio for the *n*-butylamine-initiated ROP of BLG NCA catalyzed by DMAPPCl. Inset: overlay of GPC traces at different [M]:[I]. (b) Plots of *M*_n_ and *Đ* against conversions of BLG NCA at a ratio of [M]:[I]:[Cat] = 100:1:4. Inset: overlay of GPC traces at different monomer conversions. c) GPC traces and corresponding *M*_n_ of obtained 5 block copolymers via alternate addition of BLG NCA and BLL NCA at 20 mol equiv to the initiator.

Among the aforementioned cationic catalysts, DMAPPCl demonstrated optimal catalytic performance and was therefore selected as the representative cationic catalyst for subsequent study. By varying the [M]:[I] feed ratio from 20:1 to 500:1, a series of polypeptides were successfully obtained with predicted *M*_n_ (incrementally increased from 4.5 to 109.3 kg/mol), monomodal GPC traces, and narrow dispersities (Table 1, entries 14–18, GPC traces in Figure 2a). Moreover, a linear relationship was obtained for *M*_n_ against monomer conversion in the DMAPPCl-catalyzed BLG NCA polymerization ([M]:[I]:[Cat.] = 100:1:4), demonstrating high polymerization controllability (Figure 2b).

In addition to the excellent polymerization controllability, DMAPPCl-catalyzed NCA polymerization also afforded polypeptides with defined secondary structures using BLG NCA as the proof-of-concept demonstration. PBLG obtained using this method at variable chain lengths (DP = 21, 54, 101) all showed characteristic circular dichroism (CD) signals of α-helix structures with a positive band at 195 nm and two negative bands at 208 and 222 nm (Figure S4). Furthermore, DMAPPCl-catalyzed NCA polymerization can generate polypeptides as block copolymers containing five blocks of alternating BLG and *N*ε-*tert*-butyloxycarbonyl-l-lysine (BLL) units through the stepwise feeding of BLG NCA and BLL NCA, respectively. *M*_n_ of the obtained block copolymer gradually increased with each step of feeding and was always consistent with the theoretical value, accompanied by narrow dispersity after adding each block (Figure 2c). Taken together, these results demonstrated the living characteristics of this DMAPPCl-catalyzed NCA polymerization that possesses fast polymerization rate, high controllability, and a narrow dispersity.

**Figure 3:**
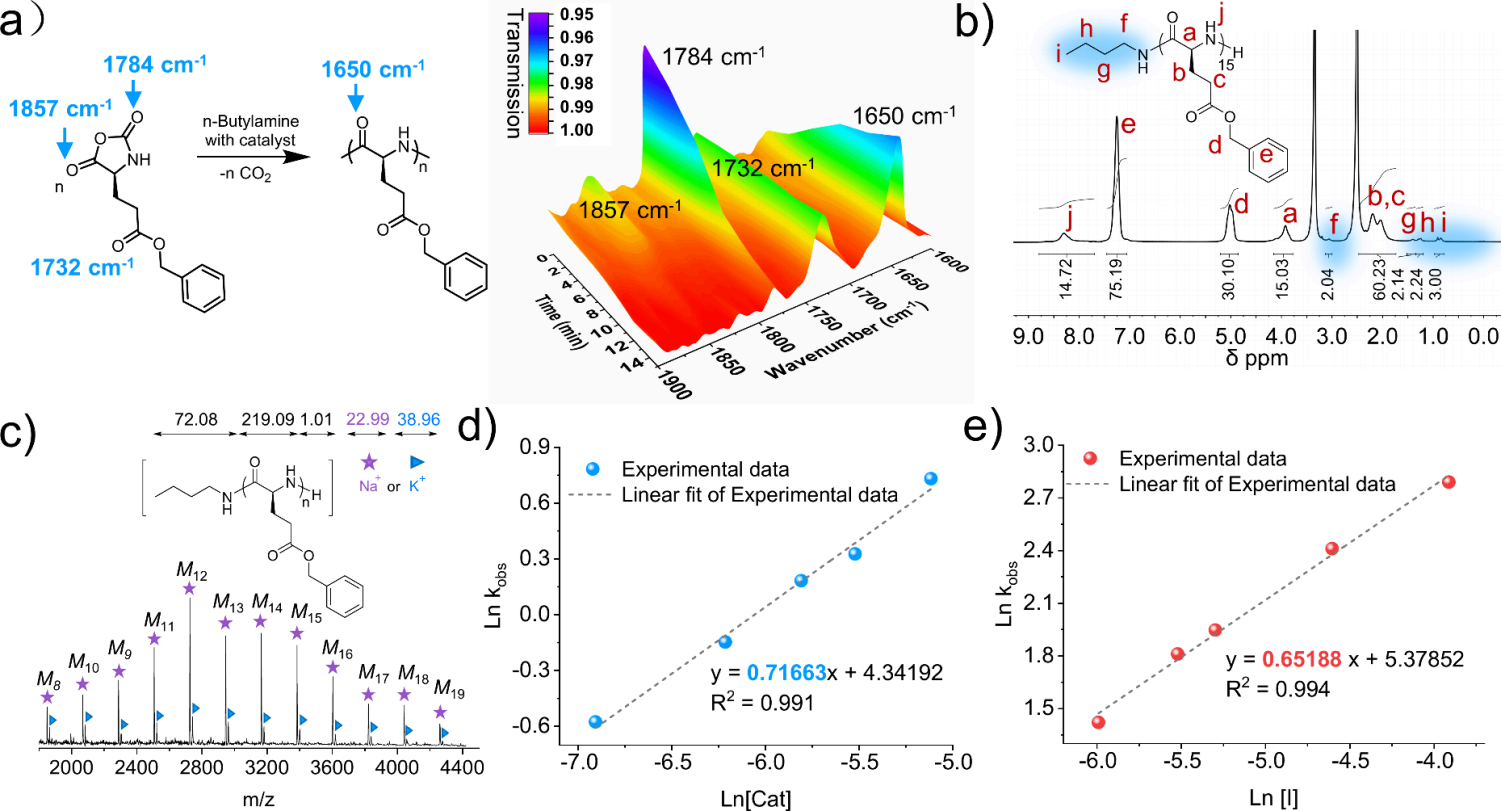
Characterization and kinetics study of polypeptides synthesized from *n*-butylamine-initiated ROP of BLG NCA using DMAPPCl as the catalyst. (a) FTIR characterization on the changes of BLG-NCA and PBLG concentration over reaction time of polymerization ([M]/[I]/[Cat] = 20/1/4). (b) ^1^H NMR spectrum of PBLG (400 MHz, DMSO-*d*_6_). (c) MALDI-TOF MS spectrum of PBLG. (d) Plot of Ln *k*_obs_ vs Ln [Cat.] at different catalyst concentrations. (e) Plot of Ln *k*_obs_ vs Ln [I] at different initiator concentrations.

Using Fourier transform infrared spectroscopy (FTIR) to monitor the *n*-butylamine-initiated polymerization of BLG NCA with DMAPPCl as the catalyst ([M]:[I]:[Cat] = 20:1:4) showed a rapid decrease of the C5 carbonyl peak (1857 cm^–1^) and the C2 carbonyl peak (1784 cm^–1^) of BLG within a few minutes, and a gradual increase of the amide carbonyl absorption peak (1650 cm^–1^), indicating the consumption of NCAs and formation of an amide backbone for the resulting polypeptide, respectively (Figure 3a). ^1^H NMR characterization obtained an integration ratio of 3:15 for Hi (C-terminal CH_3_):Ha (backbone CH), which indicates the incorporation of the C-terminal *n*-butylamine residue in obtained PBLG via initiation (Figure 3b and Figure S5). The *M*_n_ obtained from ^1^H NMR analysis was close to that from GPC characterization, and both were consistent with the theoretic value, indicating controlled polymerization (Table S1, entry 2, GPC traces in Figure S3). Notably, both ^1^H NMR and HPLC characterization indicated complete removal of the DMAPPCl catalyst from the resulting polypeptides after purification (Figure 3b and Figure S6). Matrix-assisted laser desorption ionization-time-of-flight (MALDI-TOF) mass spectroscopy analysis echoed the conclusion from ^1^H NMR analysis that the obtained PBLG was composed of BLG residues bearing a C-terminal *n*-butylamine group (Figure 3c).

A polymerization kinetics study showed that addition of 4% DMAPPCl catalyst substantially accelerated *n*-butylamine-initiated polymerization on BLG NCA, with the *k*_obs_ value increasing from 0.08 h^–1^ (without catalyst^31^) to 2.90 h^–1^ (with catalyst) (Figure S7). Notably, this catalyzed NCA polymerization exhibits a classical two-stage propagation, which may come from the transition of the polypeptide chain from nonhelical to α-helical conformation during chain growth.^50,51^ To further study the catalytic performance of the DMAPPCl catalyst, we investigated the polymerization kinetics at various catalyst concentrations and chain lengths. Increasing the amount of DMAPPCl incrementally from 0.5% to 3% relative to the BLG NCA gradually increased the polymerization rate, with the *k*_obs_ value increasing from 0.56 to 2.08 h^–1^ (Figure S8a). At fixed monomer and DMAPPCl catalyst concentrations ([M]_0_ = 0.2 M, [Cat.]_0_ = 6 × 10^–3^ M), the *k*_obs_ values of BLG NCA polymerization were 4.14, 6.12, 7.01, 11.15, and 16.29 h^–1^, respectively, increasing incrementally with the decrease of chain lengths ([M]_0_/[I]_0_ = 80, 50, 40, 20, and 10) (Figure S8b). The plots of ln *k*_obs_ versus Ln [Cat.] and Ln [I] yielded linear relationships with slopes of 0.72 and 0.65, respectively (Figure 3d,e). As a result, the kinetic equation of the DMAPPCl-catalyzed and *n*-butylamine-initiated ROP of BLG NCA was determined to be *R*_p_ = −d[M]/d*t* = *k*_p_[Cat]^0.72^[I]^0.65^[NCA].

To verify the versatility of DMAPPCl-catalyzed NCA polymerization, various primary amines were used to initiate BLG NCA polymerization ([M]_0_/[I]_0_ = 50:1), including sterically hindered amines (e.g., amantadine, cyclohexylamine, etc.) and functional amines (e.g., 3-aminopropyne, 3-azido-1-propanamine, etc.). It was found that all polymerization could be completed within 40 min, producing PBLGs with controllable molecular weight (*M*_n_ = 11.2–13.2 kg/mol) and a narrow dispersity (*Đ* = 1.04–1.17) (Table 2 entries 1–6, GPC traces in Figure S9). Additionally, using 4-arm-PEG-NH_2_ as the initiator, BLG NCA was completely converted to 4-arm-PBLG within 1.5 h with the expected molecular weight (*M*_n_ = 47.0 kg/mol) and a narrow dispersity (*Đ* = 1.19) (Table 2 entry 7, GPC traces in Figure S9). For NCAs bearing diverse substituent groups, such as *N*ε-*tert*-butyloxycarbonyl-l-lysine (BLL) NCA, *N*ε-benzyloxycarbonyl-l-lysine (cbz-l-Lys) NCA, γ-*tert*-butylester-l-glutamate (tbu-l-Glu) NCA, and *N*δ-*tert*-butyloxycarbonyl-l-ornithine (Boc-l-Orn) NCA, the DMAPPCl catalyst can also facilitate accelerated and controlled polymerization (Table 2 entries 8–11, GPC traces in Figure S9; Table S2, GPC traces in Figure S10). Moreover, random copolymerization on NCA mixtures was also successfully achieved using DMAPPCl catalyst to obtain amphiphilic polypeptides for potential biological applications (Table 2 entries 12–14, GPC traces in Figure S9). ^1^H NMR results showed that the composition of obtained copolymers were consistent with the feed ratio (Figures S11–S13). In addition to THF, DMAPPCl-catalyzed NCA polymerization is also compatible with normal organic solvents such as toluene, CH_2_Cl_2_, EtOAc, dioxane, and DMF to afford polymers with controlled polymer length, monomodal GPC traces, and narrow dispersities (Table 2 entries 15–19, Figure S9). DMAPPCl-catalyzed NCA polymerization exhibits superior performance in weak polar solvents (e.g., THF and CH_2_Cl_2_) compared to strong polar solvents (e.g., DMF), as strong polar solvents may interfere with the interaction between the catalyst and monomers. Notably, even in undried solvents, DMAPPCl-catalyzed NCA polymerization proceeded with control of polymer length and a narrow dispersity (Table 2 entry 20, GPC trace in Figure S9).

**Table 2:**
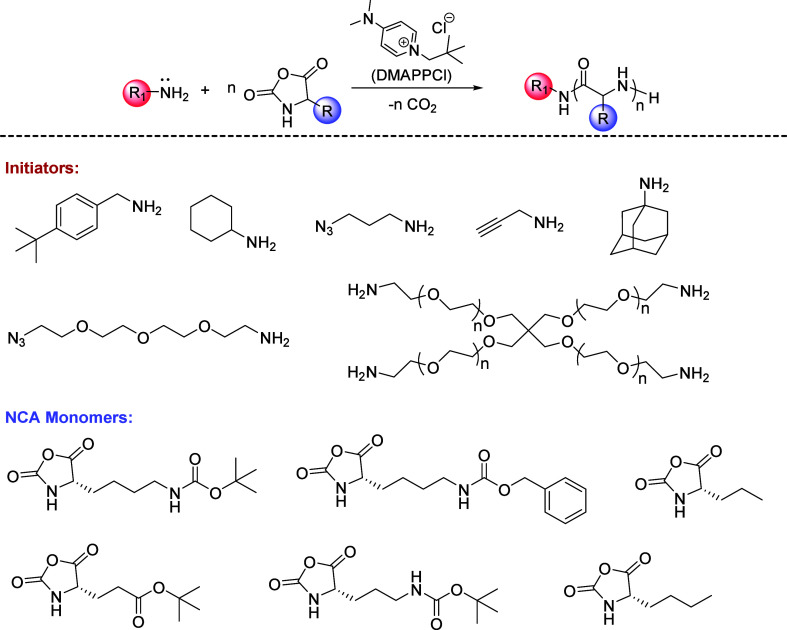
Synthesis and GPC Characterization of Polypeptides via DMAPPCl-Catalyzed NCA Polymerization Using Variable Primary Amine Initiators in Different Solvents^a^

entry	solvent	monomer	initiator	[M]:[I]:[Cat]	time	*M*_n,calcd_^b^ (kg/mol)	*M*_n,SEC_ (kg/mol)	*Đ*
1	THF	BLG	*tert*-butylbenzylamine	50/1/4	30 min	11.1	11.9	1.08
2	THF	BLG	cyclohexanamine	50/1/4	30 min	11.1	12.5	1.14
3	THF	BLG	3-azido-1-propanamine	50/1/4	30 min	11.0	11.2	1.17
4	THF	BLG	3-aminopropyne	50/1/4	30 min	11.1	13.2	1.10
5	THF	BLG	amantadine	50/1/4	30 min	11.1	12.3	1.04
6	THF	BLG	11-azido-3,6,9-trioxaundecan-1-amine	50/1/4	40 min	11.2	12.8	1.11
7	THF	BLG	4-arm-PEG-NH_2_	200/1/4	1.5 h	48.8	47.0	1.19
8	THF	BLL	*n*-butylamine	50/1/4	35 min	11.5	12.8	1.22
9	THF	cbz-l-Lys	*n*-butylamine	50/1/4	4 h	13.2	12.4	1.02
10	THF	tbu-l-Glu	*n*-butylamine	50/1/4	1.5 h	9.3	8.7	1.08
11	THF	boc-l-Orn	*n*-butylamine	50/1/4	2 h	10.8	11.3	1.21
12	THF	BLG+l-Nle	*n*-butylamine	(50 + 50)/1/4	3 h	16.6	16.8	1.06
13	THF	BLL+l-Nle	*n*-butylamine	(50 + 50)/1/4	3 h	17.0	16.7	1.04
14	THF	BLL+l-Nva	*n*-butylamine	(50 + 50)/1/4	4 h	17.0	17.0	1.15
15	toluene	BLG	*n*-butylamine	100/1/4	50 min	22.0	22.0	1.08
16	CH_2_Cl_2_	BLG	*n*-butylamine	100/1/4	1.5 h	22.0	22.0	1.14
17	EtOAc	BLG	*n*-butylamine	100/1/4	1.2 h	22.0	21.6	1.07
18	dioxane	BLG	*n*-butylamine	100/1/4	1.5 h	22.0	23.6	1.10
19	DMF	BLG	*n*-butylamine	100/1/4	4 h	22.0	18.1	1.18
20^c^	THF	BLG	*n*-butylamine	100/1/4	1.5 h	22.0	21.0	1.05

a All NCA polymerizations were performed at room temperature with [M]_0_ = 0.2 M. All polypeptides were characterized by GPC using 0.01 M LiBr in DMF as the mobile phase at a flow rate of 1 mL/min at 50 °C. *M*_n,SEC_ is the number-average molecular weight. *Đ* is the dispersity index, d*n*/d*c* (PBLG) = 0.104 mL/g.

b *M*_n,calcd_ is the theoretical number-average molecular weight.

c Undried THF was used as the solvent in the polymerization reaction.

**Figure 4:**
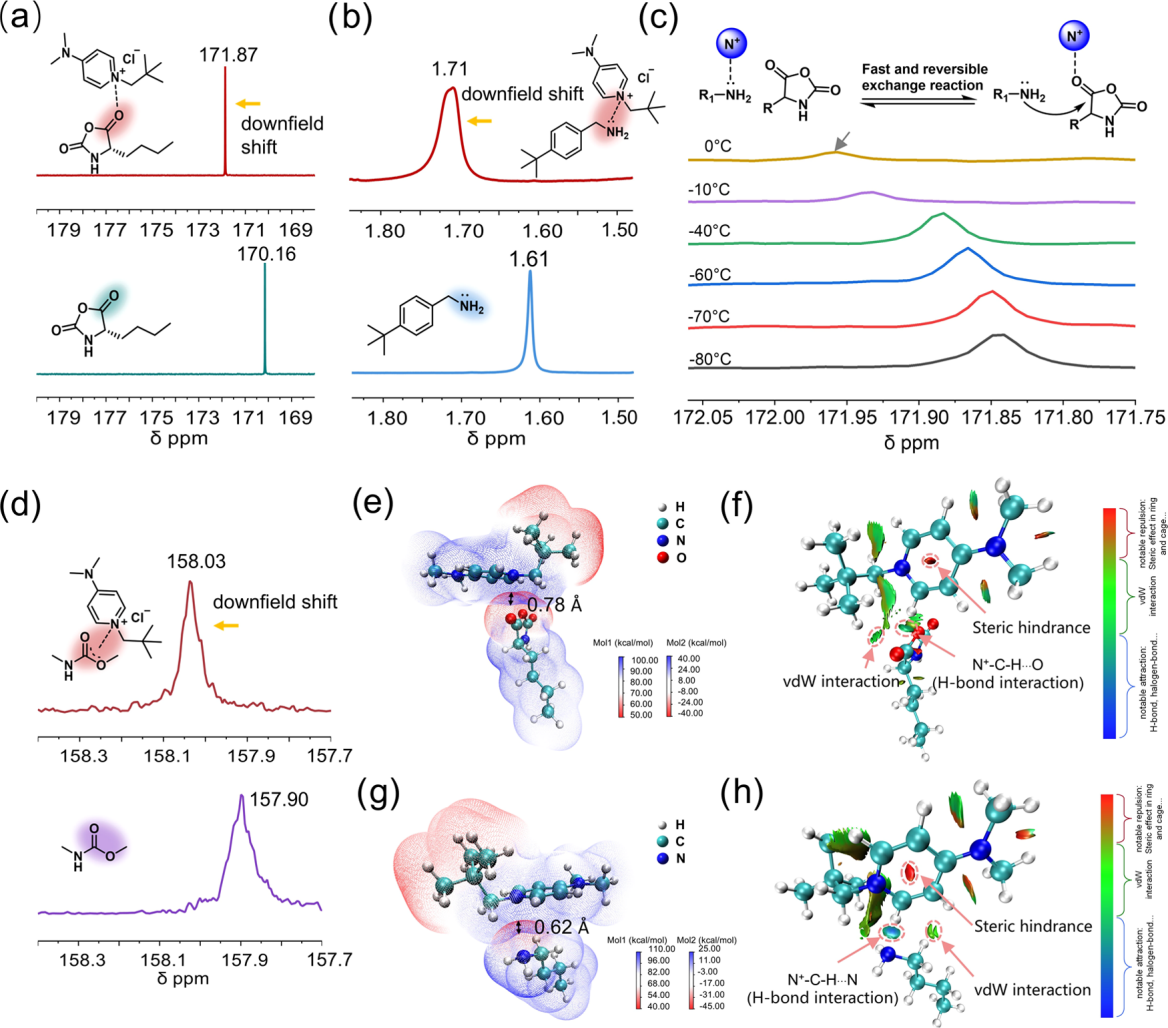
Mechanism of DMAPPCl catalyzed ROP of NCAs with an accelerated reaction rate and controlled polymerization. (a) Comparison of ^13^C NMR spectra for Nle NCA only and Nle NCA mixing with DMAPPCl (1:1, molar ratio) in CD_2_Cl_2_. b) Comparison of ^1^H NMR spectra for *tert*-butylbenzylamine only and *tert*-butylbenzylamine mixing with DMAPPCl (1:1, molar ratio) in CDCl_3_/CD_2_Cl_2_ (CDCl_3_ :  CD_2_Cl_2_ = 1 : 1, v/v). c) ^13^C NMR spectra for Nle NCA, *n*-butylamine and DMAPPCl mixture at the molar ratio of 1:1:1 in CD_2_Cl_2_ at variable temperatures (−80 to 0 °C). (d) Comparison of ^13^C NMR spectra for methyl *N*-methylcarbamate only and methyl *N*-methylcarbamate mixed with DMAPPCl catalyst (1:1, molar ratio) at room temperature in CD_2_Cl_2_. (e) Electrostatic potential map of electrostatic interaction between *n*-butylamine and DMAPPCl. (f) Local NCI analysis of hydrogen-bond interaction of *n*-butylamine and DMAPPCl. (g) Electrostatic potential map of electrostatic interaction between Nle NCA and DMAPPCl. (h) Local NCI analysis of hydrogen-bond interaction of Nle NCA and DMAPPCl.

The superior performance of DMAPPCl-catalyzed NCA polymerization in preparing polypeptides encouraged us to explore the catalytic mechanism. To facilitate the ^13^C NMR spectra analysis, we used Nle NCA bearing a simple structure as a model monomer for the mechanistic study. A close comparison of the ^13^C NMR spectra of an Nle NCA and DMAPPCl/Nle NCA mixture (molar ratio 1:1) revealed that the C5 carbonyl on Nle NCA shifts downfield from 170.16 to 171.87 ppm (Figure 4a and Figure S14), which is proposed to come from the activation (increasing the electrophilicity) of the C5 carbonyl group on Nle NCA by the N^+^ in the pyridine ring of DMAPPCl catalyst. The ^1^H NMR characterization showed that the signal of the primary amine group on *tert*-butylbenzylamine shifted downfield from 1.61 to 1.71 ppm in the presence of DMAPPCl, indicating that the catalyst will deactivate primary amines and inhibit side reactions due to the basicity of the primary amine (Figure 4b and Figure S15). FTIR spectroscopy also confirmed that the catalyst could both activate NCAs and passivate primary amines (Figure S16).

Using low-temperature ^13^C NMR to characterize the interaction of DMAPPCl to NCAs and primary amines at the molar ratio of 1:1:1, we found that with the increase of temperature from −80 to 0 °C, the C5 carbonyl on Nle NCA gradually shifted downfield from 171.84 to 171.96 ppm, indicating an incrementally increased interaction between DMAPPCl and Nle NCA (Figure 4c and Figure S17). This implies that the interaction of DMAPPCls with primary amines and NCAs is dynamic, resulting in the transfer of partial DMAPPCls from passivating primary amines to activating NCAs when the temperature increases. This result is consistent with the experiment that increasing the equivalent of catalyst relative to NCA can achieve not only improved polymerization controllability but also an accelerated polymerization speed. All of the above studies indicate that DMAPPCl can both activate NCAs and passivate primary amines, with rapid dynamic transfer between these two states.

We continued to study how DMAPPCl works in the decarboxylation step during chain propagation of NCA polymerization. Due to the instability of carbamate intermediates at room temperature, we used methyl *N*-methylcarbamate as a model for related mechanistic study. ^13^C NMR analysis revealed that the carbonyl of methyl *N*-methylcarbamate shifted downfield from 157.90 to 158.03 ppm in the presence of DMAPPCl, indicating activation of carbamate by the cationic DMAPPCl (Figure 4d and Figure S18), which in turn assists the intramolecular transfer of hydrogen atoms and the detachment of CO_2_, thereby accelerating decarboxylation, which is similar to the Lewis acid assisted decarboxylation.^52^

Density functional theory (DFT) calculations on the electrostatic potential map of DMAPPCl showed that the positive charge of DMAPPCl is delocalized on the whole structure, allowing multiple catalytic sites to interact with NCAs and primary amines (Figure S2). The delocalized positive charge on the DMAPPCl conjugated ring can form cation–dipole interactions to the C5 carbonyl of NCA and the primary amine, with the mutual penetration distances of electrostatic potential energy surfaces measured as 0.78 and 0.62 Å, respectively (Figure 4e and 4g). The binding energies of DMAPPCl to NCA and the primary amine are 5.27 and 6.63 kcal/mol, respectively, indicating that DMAPPCl prefers to passivate primary amines rather than activate NCAs (Figure S19). This result is consistent with the conclusion of the aforementioned low-temperature NMR study that the enhanced mobility of DMAPPCl facilitates its transfer from primary amines to NCAs when the temperature rises. Moreover, the noncovalent interaction (NCI) analysis results show that the proton of C–H at the α position of the positively charged N atom can activate NCA and passivate the primary amine through C–H–O and C–H–N hydrogen bonding interactions, respectively (Figure 4f and 4h). The binding energy of DMAPPCl with NCA through C–H–O hydrogen bonding interaction is 5.47 kcal/mol, which is comparable to that of the cation–dipole interaction (Figure S20).

**Figure 5:**
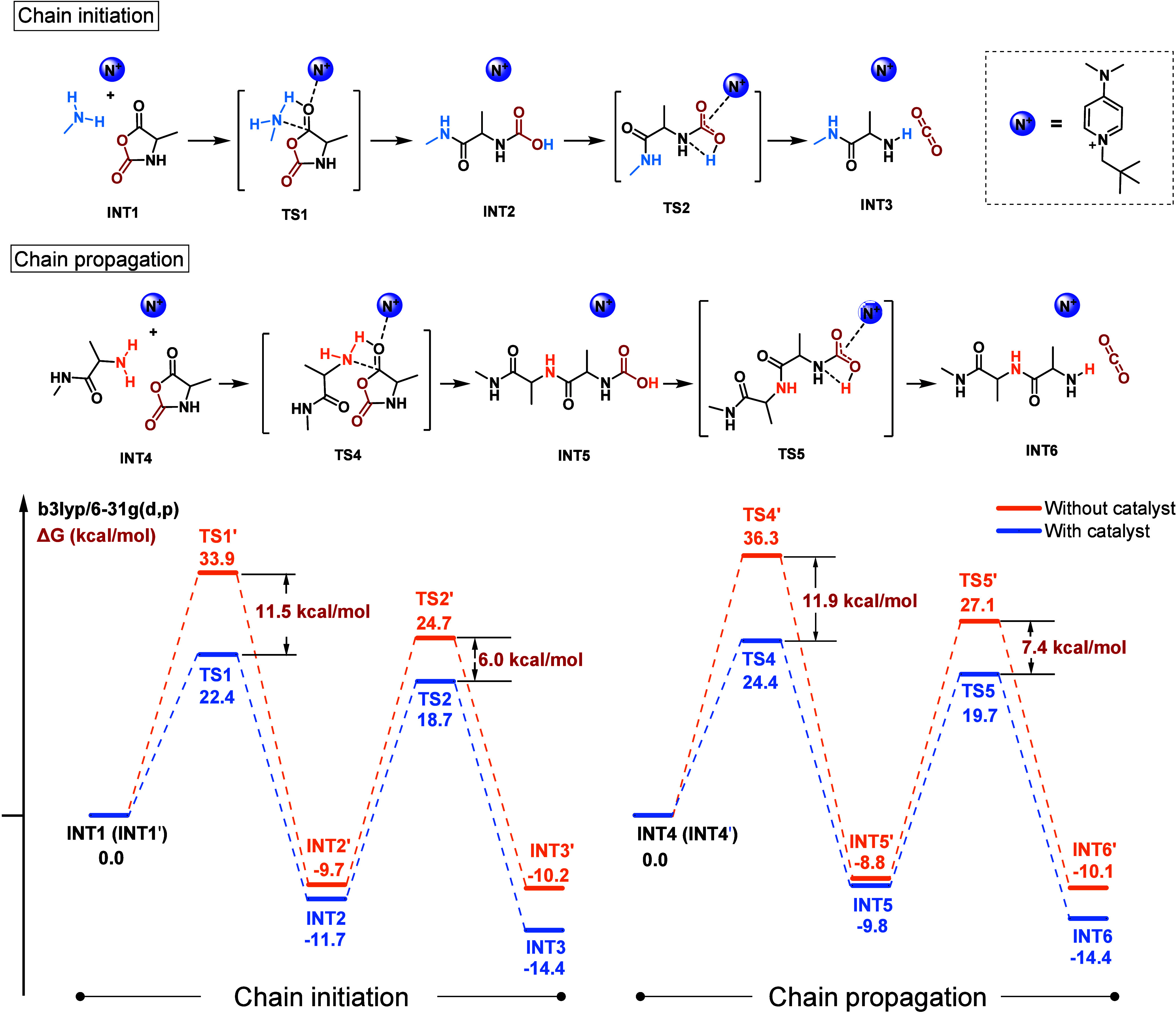
DFT calculation on Gibbs free energy profiles of methylamine-initiated ROP of Ala NCA using DMAPPCl as the catalyst.

To get a deeper mechanistic understanding of DMAPPCl-catalyzed NCA polymerization, DFT calculation was applied to simulate the chain initiation and propagation processes using methylamine-initiated alanine NCA polymerization as a model. In the chain initiation stage, DMAPPCl can activate the C5 carbonyl of NCA through a cation–dipole interaction (state TS1), resulting in an 11.5 kcal/mol reduction of the energy barrier for nucleophilic attack of primary amine on NCA compared to the transition state TS1′ without DMAPPCl catalyst (Figure 5). In addition, DMAPPCl can assist in the release of CO_2_, reducing the energy barrier of decarboxylation by 6.0 kcal/mol. In the chain propagation stage, DMAPPCl lowered the energy barriers of nucleophilic addition to NCAs and intramolecular decarboxylation by 11.9 and 7.4 kcal/mol, respectively, substantially accelerating NCA polymerization (Figure 5). The above results altogether demonstrated that the quaternary ammonium catalyst exhibits three essential functions: (1) activating NCAs to accelerate the polymerization, (2) assisting the decarboxylation of carbamate to accelerate the polymerization, and (3) passivating the amino active centers moderately to inhibit the occurrence of side reactions and realize controlled polymerization (Figure S21).

**Figure 6:**
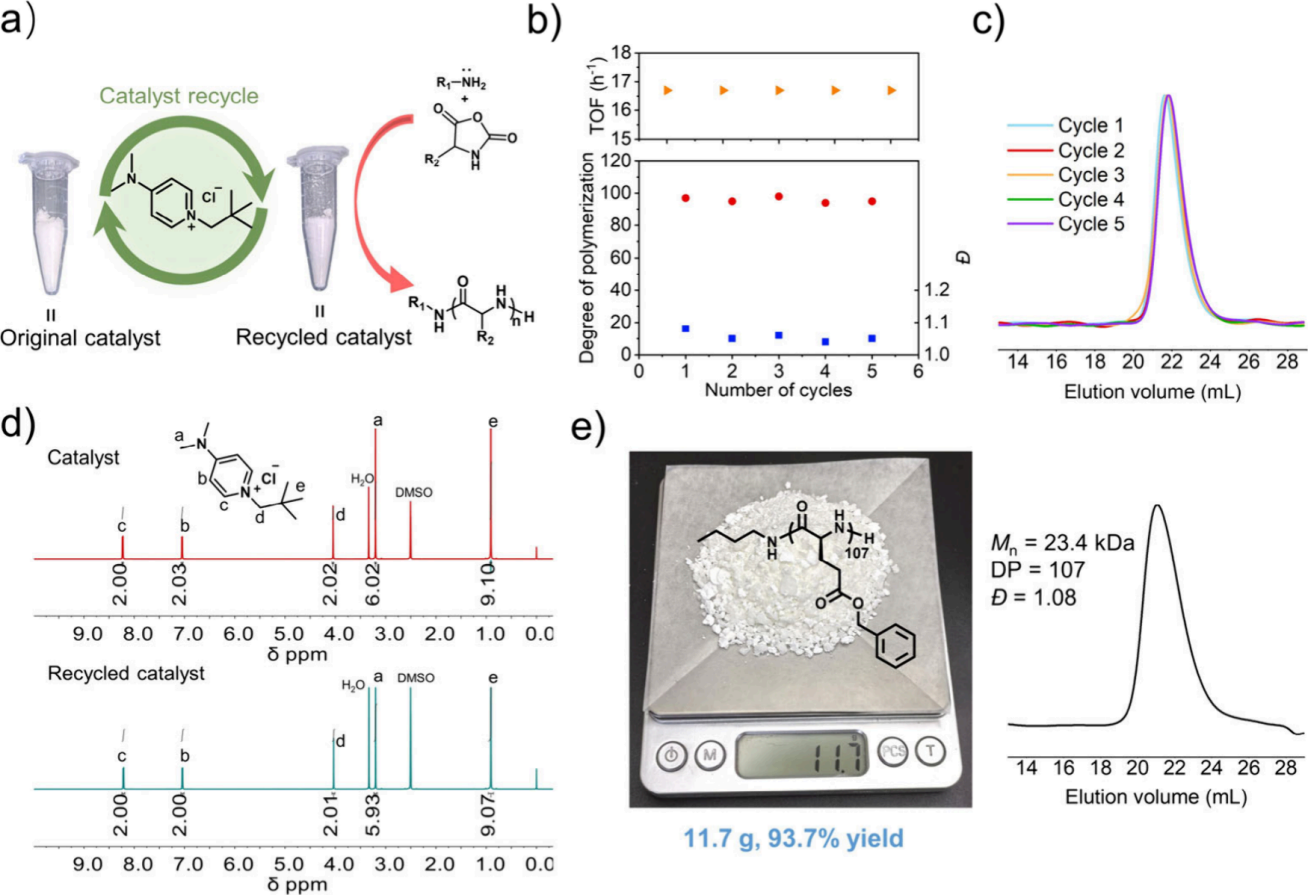
Catalyst recovery and gram-scale synthesis of polypeptide. (a) Schematic diagram of catalyst recycling in NCA polymerization. (b) Scatter plots of TOF, DP, and *Đ* versus the number of catalyst cycles (starting from cycle 1, each recycle of the catalyst was *n* + 1 cycle) for the *n*-butylamine-initiated ROP of BLG NCA catalyzed by DMAPPCl. TOF denotes turnover frequency. Calculated by ([M]/[Cat.]) × conversion/time. (c) Overlay of GPC traces from different cycles. (d) Comparison of ^1^H NMR spectra for DMAPPCl (red) and recycled DMAPPCl (cyan). 9e) *n*-Butylamine-initiated ROP of BLG NCA catalyzed by recycled DMAPPCl at a decagram scale using undried THF as the solvent. *M*_n_, DP, and *Đ* were determined by GPC.

The DMAPPCl catalyst was well recyclable, with a recovery ratio ranging from 85% to 95% (Figure 6a). The recycled DMAPPCl catalyst could be easily obtained by centrifuging the reaction mixture after NCA polymerization was completed. Even after recycling five times, the catalytic activity of DMAPPCl remains robust, maintaining a turnover frequency (TOF) value of approximately 16.7 h^–1^ (Figure 6b). The molecular weight of the obtained polypeptides catalyzed by the recovered DMAPPCl closely matched the theoretical value, featuring narrow dispersity and monomodal GPC traces (Figure 6c). ^1^H NMR analysis confirmed that the recycled catalyst remains unchanged, revealing the feasibility of repeated recycling (Figure 6d). Using recycled DMAPPCl catalyst, decagram-scale polymerization of BLG NCA was conducted in undried THF to obtain long chain PBLG in 93.7% yield with an expected *M*_n_ of 23.4 kg/mol (DP = 107) and a narrow dispersity at 1.08 (Figure 6e).

In summary, we develop a highly efficient single-center trifunctional cationic-catalyst strategy to achieve fast and controlled polymerization on NCAs using commercially available quaternary ammonium salts as the catalysts. This quaternary ammonium salt catalyzed and primary amine-initiated NCA polymerization enables quick synthesis of polypeptides and amphiphilic copolypeptides with predictable molecular weight and narrow dispersities, compatible with different primary amine initiators, diversified NCA structures, and a wide range of solvents. The optimal trifunctional cationic catalyst can dynamically transfer between activating NCAs and passivating primary amines as well as activate carbamate intermediates to accelerate decarboxylation, thereby remarkably accelerating the polymerization rate and enhancing the polymerization controllability simultaneously. Computational studies showed that conjugated cationic catalysts manifest a delocalized positive charge on the whole structure, allowing multiple catalytic sites to interact with NCAs and primary amines through cation–dipole interaction and nonclassical hydrogen binding (C–H–O and/or C–H–N) interaction. All of these advantages and the good recyclability of trifunctional quaternary ammonium catalysts imply a wide application of this cationic-catalyst strategy in polymerization chemistry not limited to NCA polymerization.

## Supporting Information Available

Materials, experimental procedures, tables, supplemental figures, NMR data, and DFT coordinates (PDF)
