# Supplementary material for: Single-Center Trifunctional Organocatalyst Enables Fast and Controlled Polymerization on N-Carboxyanhydride
Source: ACS Cent Sci. 2024 Oct 29;11(3):382–92. doi: 10.1021/acscentsci.4c01346 (PMC11950855; doi:10.1021/acscentsci.4c01346)
Supplement: Supplementary file 1 — oc4c01346_si_001.pdf [file oc4c01346_si_001.pdf]

Supporting Information

Single-Center Trifunctional Organocatalyst Enables Fast and Controlled Polymerization on *N*-Carboxyanhydride

Kang Chen[a,b,c], Yueming Wu*[a,b,d], Minzhang Chen[b,d], Jiangzhou Wang[b,d], Min Zhou[b,d], Xin Chen[d], Runhui Liu*[a,b,c,d]

[a] State Key Laboratory of Bioreactor Engineering, East China University of Science and Technology, Shanghai 200237, China

[b] Shanghai Frontiers Science Center of Optogenetic Techniques for Cell Metabolism, Frontiers Science Center for Materiobiology and Dynamic Chemistry, Engineering Research Center for Biomedical Materials of Ministry of Education, Key Laboratory of Specially Functional Polymeric Materials and Related Technology (Ministry of Education), School of Materials Science and Engineering, East China University of Science and Technology, Shanghai 200237, China

[c] Department of Biomaterials and Stem Cells, Suzhou Institute of Biomedical Engineering and Technology, Chinese Academy of Sciences, Suzhou, 215163, China

[d] Key Laboratory for Ultrafine Materials of Ministry of Education, School of Materials Science and Engineering, East China University of Science and Technology, Shanghai 200237, China

*The Corresponding Author: Runhui Liu (rliu@ecust.edu.cn), Yueming Wu (yuemingwu@ecust.edu.cn)

Materials

Anhydrous tetrahydrofuran (THF), dioxane, dichloromethane (CH_2Cl_2), *N*, *N*-dimethylformamide (DMF), toluene, dioxane and ethyl acetate (EtOAc) were purchased from Sigma-Aldrich and used without purification as received. 4-dimethylamino-1-neopentylpyridinium chloride (DMAPPCl) were purchased from RHAWN®, 1,3-dimethylimidazolium chloride (DMMCl), 5-butyl-7-methyl-1,5,7-triazabicyclo[4.4.0]dec-5-enium (MTBD) were purchased from Adamas-beta®, 8-butyl-1,8-diazabicyclo[5.4.0]undec-7-enium (DBU) were purchased from Tokyo Chemical Industry Co., Ltd. L-glutamic acid γ-benzyl ester, *N*ε-*tert*-butyloxycarbonyl-L-lysine, *N*ε-benzyloxycarbonyl-L-Lysine, *N*-*tert*-butyloxycarbonyl-L-ornithine, and other reagents were purchased from Adamas-beta®. Cyclohexanamine was purchased from Shanghai Macklin Biochemical Technology Co., Ltd. 4-arm-PEG-NH2 was purchased from Shanghai Macklin Biochemical Technology Co., Ltd. Undried solvents including dichloromethane (CH_2Cl_2), ethyl acetate (EtOAc), hexane, acetonitrile (MeCN) were purchased from Adamas-beta®. Solvents used in synthesizing α-amino acid *N*-carboxyanhydride (NCA) were freshly distilled or dried over $MgSO_4$ before use.

Instrumentation

Gel permeation chromatography (GPC) was performed on a system equipped with a Waters 1515 isocratic HPLC pump, a Brookhaven BI-MwA multi-angle light scattering detector and a Waters 2414 refractive index detector. Separations were performed using a Tosoh TSKgel Alpha-2500 column (particle size 7 μm, 300 × 7.8 mm), a Tosoh TSKgel Alpha-3000 column (particle size 7 μm, 300 × 7.8 mm) and a Tosoh TSKgel Alpha-4000 column (particle size 10 μm, 300 × 7.8 mm) linked in series using DMF, supplemented with 0.01 M LiBr, as the mobile phase at a flow rate of 1 mL/min at 50°C. The BI-MwA detector was calibrated using narrow polystyrene standards. Samples were filtered through a 0.22 μm PTFE filter before analysis. The absolute molecular weights of polymers were determined using BIC ParSEC Software. dn/dc(PBLG) = 0.104 mL/g, dn/dc(PBLL) = 0.123 mL/g. High-performance liquid chromatography (HPLC) analysis was carried out on a Shimadzu LC-20AR HPLC System equipped with a Gemini 5 μm NX-C18 column. Synthesized intermediates were purified using

a SepaBean machine equipped with Sepaflash columns produced by Santai Technologies Inc. in China. Nuclear magnetic resonance (NMR) spectra were recorded on a Bruker Avance III 400 MHz or an Ascend 600 MHz spectrometer. Low-temperature NMR study was recorded on a Bruker Avance III HD 500 MHz spectrometer. ^{1}H NMR chemical shifts were referenced to the resonance for residual protonated solvent (*δ* 5.32 for CD_2Cl_2, *δ* 7.26 for $CDCl_3$, *δ* 4.79 for D_2O, *δ* 2.50 for DMSO-d_6, *δ* 3.31 for Methanol-d_4). ^{13}C NMR chemical shifts were referenced to the resonance for residual solvent (*δ* 53.84 for CD_2Cl_2, *δ* 77.16 for $CDCl_3$, *δ* 39.52 for DMSO-d_6, *δ* 49.00 for Methanol-d_4, *δ* 1.32 and 118.26 for MeCN-d_3). In situ Fourier transform infrared (FTIR) spectra were recorded on Perkin-Elmer Spectrum 100 spectrometer with an attenuated total reflectance (ATR) accessory. High-resolution ESI Mass Spectrum (HRESI-MS) was recorded using a Waters Xevo G2 TOF mass spectrometer. Matrix-assisted laser desorption ionization-time of flight (MALDI-TOF) mass spectra were collected using an AB SCIEX 4800plus MALDI-TOF analyzer in reflection mode equipped with a nitrogen laser emitting at 337 nm using 2,5-dihydroxybenzoic acid (DHB) as the matrix.

Synthesis of γ-benzyl-L-glutamate (BLG) NCA monomer

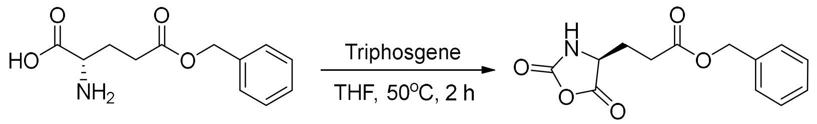

γ-benzyl-L-glutamate (BLG) NCA monomer was synthesized by following the precedent literature with modifications[1]. L-glutamic acid γ-benzyl ester (5.0 g, 21.1 mmol) was suspended in 50 mL anhydrous THF in a dry round-bottom flask charged with a magnetic stirring bar. Then a solution of triphosgene (2.8 g, 9.3 mmol) in anhydrous THF (20 mL) was added dropwise into the reaction flask in an ice bath under nitrogen protection. The reaction was heated to 50°C and stirred under nitrogen for 2 hours. After cooling to room temperature, the mixture was concentrated under reduced pressure and then the residue was re-dissolved in EtOAc (50 mL). The mixture was washed with cold deionization water (50 mL) and cold brine (50 mL) rapidly, and then was dried over anhydrous $MgSO_4$. The crude NCA was recrystallized thrice using dried EtOAc/hexane to afford a white crystal (3.4 g, 61% yield). ^{1}H NMR (400 MHz, $CDCl_3$) *δ* 7.32 – 7.43 (m, 5H), 6.39 (s, 1H), 4.33 – 4.41 (m, 1H), 2.56 – 2.64 (m, 2H), 2.23 – 2.35 (m, 1H), 2.04 – 2.19 (m, 1H). The ^{1}H NMR spectrum of this product was consistent

to that in precedent literature[1].

Synthesis of *N*ε-*tert*-butyloxycarbonyl-L-lysine (BLL) NCA monomer

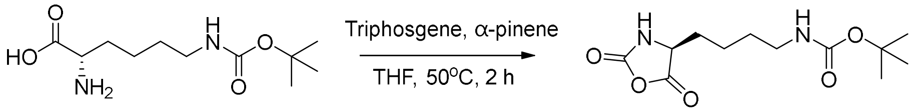

*N*ε-*tert*-butyloxycarbonyl-L-lysine (BLL) monomer was synthesized by following the precedent literature with modifications[1]. *N*ε-*tert*-butyloxycarbonyl-L-lysine (5.0 g, 20.3 mmol) and α-pinene (7.3 g, 53.6 mmol) were suspended in 80 mL anhydrous THF in a dry round-bottom flask charged with a magnetic stirring bar. Then a solution of triphosgene (2.7 g, 8.9 mmol) in anhydrous THF (20 mL) was added dropwise into the reaction flask in an ice bath under nitrogen protection. The reaction was heated to 50°C and stirred under nitrogen for 2 hours. After cooling to room temperature, the mixture was concentrated under reduced pressure and then the residue was re-dissolved in EtOAc (50 mL). The mixture was washed with cold deionization water (50 mL) and cold brine (50 mL) rapidly, and then was dried over anhydrous $MgSO_4$. The crude NCA was recrystallized thrice using dried EtOAc/hexane to afford a white crystal (3.2 g, 57% yield). ^{1}H NMR (400 MHz, $CDCl_3$) δ 6.96 (s, 1H), 4.29 – 4.36 (m, 1H), 3.06 – 3.20 (m, 2H), 1.94 – 2.07 (m, 1H), 1.79 – 1.91 (m, 1H), 1.34 – 1.63 (m, 13H). The ^{1}H NMR spectrum of this product was consistent to that in precedent literature[1].

Synthesis of *N*ε-Benzyloxycarbonyl-L-Lysine (cbz-L-Lys) NCA monomer

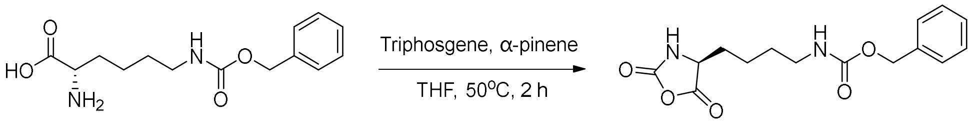

*N*ε-Benzyloxycarbonyl-L-Lysine (cbz-L-Lys) NCA monomer was synthesized by following the precedent literature with modifications[1]. *N*ε-Benzyloxycarbonyl-L-Lysine (5.0 g, 17.8 mmol) and α-pinene (6.4 g, 46.8 mmol) were suspended in 40 mL anhydrous THF in a dry round-bottom flask charged with a magnetic stirring bar. Then a solution of triphosgene (2.3 g, 7.8 mmol) in anhydrous THF (20 mL) was added dropwise into the reaction flask in an ice bath under nitrogen protection. The reaction was heated to 50°C and stirred under nitrogen for 2 hours. After cooling to room temperature, the mixture was concentrated under reduced pressure

and then the residue was re-dissolved in EtOAc (50 mL). The mixture was washed with cold deionization water (50 mL) and cold brine (50 mL) rapidly, and then dried over anhydrous $MgSO_4$. The crude NCA was recrystallized thrice using dried EtOAc/hexane to afford a white crystal (2.8 g, 51% yield). ^{1}H NMR (400 MHz, $CDCl_3$) δ 7.29 – 7.43 (m, 5H), 7.00 (s, 1H), 5.04 – 5.18 (m, 2H), 4.95 (s, 1H), 4.26 (m, 1H), 3.09 – 3.29 (m, 2H), 1.70 – 2.03 (m, 2H), 1.35 – 1.59 (m, 4H). The ^{1}H NMR spectrum of this product was consistent to that in precedent literature[2].

Synthesis of *N*δ-*tert*-butyloxycarbonyl-L-Ornithine (Boc-L-Orn) NCA monomer

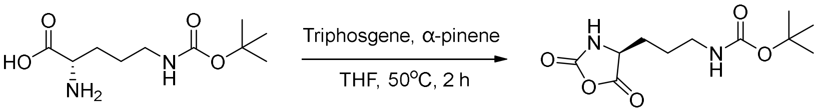

*N*δ-*tert*-butyloxycarbonyl-L-Ornithine (Boc-L-Orn) NCA monomer was synthesized by following the precedent literature with modifications[3]. *N*δ-*tert*-butyloxycarbonyl-L-Ornithine (5.0 g, 21.5 mmol) and α-pinene (7.7 g, 56.8 mmol) were suspended in 50 mL anhydrous THF in a dry round-bottom flask charged with a magnetic stirring bar. Then a solution of triphosgene (2.8 g, 9.5 mmol) in anhydrous THF (20 mL) was added dropwise into the reaction flask in an ice bath under nitrogen protection. The reaction was heated to 50°C and stirred under nitrogen for 2 hours. After cooling to room temperature, the mixture was concentrated under reduced pressure and then the residue was re-dissolved in EtOAc (50 mL). The mixture was washed with cold deionization water (50 mL) and cold brine (50 mL) rapidly, and then was dried over anhydrous $MgSO_4$. The crude NCA was recrystallized thrice using dried EtOAc/hexane to afford a white crystal (3.6 g, 64% yield). ^{1}H NMR (400 MHz, $CDCl_3$) δ 7.00 (s, 1H), 4.71 (s, 1H), 4.32 – 4.39 (m, 1H), 3.19 (t, J = 6.7 Hz, 2H), 1.96 – 2.08 (m, 1H), 1.79 – 1.90 (m, 1H), 1.59 – 1.75 (m, 4H), 1.44 (s, 9H). The ^{1}H NMR spectrum of this product was consistent to that in precedent literature[3].

Synthesis of γ-*tert*-butylester-L-glutamate (tbu-L-Glu) NCA monomer

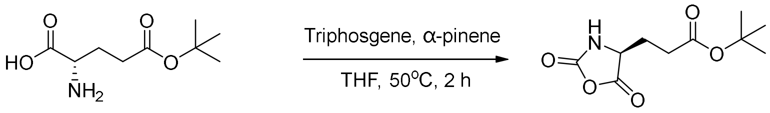

γ-*tert*-butyl ester-L-glutamate (tbu-L-Glu) NCA monomer was synthesized by following the precedent literature with modifications[1]. γ-*tert*-butyl ester-L-glutamate (5.0 g, 24.6 mmol) and α-pinene (8.8 g, 64.9 mmol) were suspended in 60 mL anhydrous THF in a dry round-bottom flask charged with a magnetic stirring bar. Then a solution of triphosgene (3.2 g, 10.8 mmol) in anhydrous THF (20 mL) was added dropwise into the reaction flask in an ice bath under nitrogen protection. The reaction was heated to 50°C and stirred under nitrogen for 2 hours. After cooling to room temperature, the mixture was concentrated under reduced pressure and then the residue was re-dissolved in EtOAc (50 mL). The mixture was washed with cold deionization water (50 mL) and cold brine (50 mL) rapidly, and then was dried over anhydrous $MgSO_4$. The crude NCA was recrystallized thrice using dried EtOAc/hexane to afford a white crystal (3.2 g, 57% yield). ^1H NMR (400 MHz, $CDCl_3$) δ 6.62 (s, 1H), 4.34 – 4.41 (m, 1H), 2.41 – 2.50 (m, 2H), 2.18 – 2.30 (m, 1H), 1.97 – 2.13 (m, 1H), 1.45 (s, 9H). The ^1H NMR spectrum of this product was consistent to that in precedent literature[4].

Synthesis of L-Norleucine (L-Nle) NCA monomer

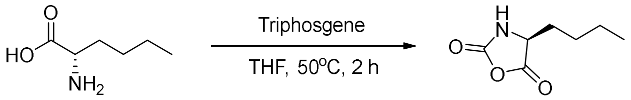

L-Norleucine (L-Nle) NCA monomer was synthesized by following the precedent literature with modification[1]. L-Norleucine (5.0 g, 38.1 mmol) was suspended in 50 mL anhydrous THF in a dry round-bottom flask charged with a magnetic stirring bar. Then a solution of triphosgene (5.0 g, 16.8 mmol) in anhydrous THF (20 mL) was added dropwise into the reaction flask in an ice bath under nitrogen protection. The reaction was heated to 50°C and stirred under nitrogen for 2 hours. After cooling to room temperature, the mixture was concentrated under reduced pressure and then the residue was re-dissolved in EtOAc (50 mL). The mixture was washed with cold deionization water (50 mL) and cold brine (50 mL) rapidly,

and then was dried over anhydrous $MgSO_4$. The crude NCA was recrystallized thrice using dried EtOAc/hexane to afford a white crystal (2.9 g, 49% yield). ^{1}H NMR (400 MHz, $CDCl_3$) δ 6.70 (s, 1H), 4.29 – 4.38 (m, 1H), 1.88 – 1.99 (m, 1H), 1.75 – 1.86 (m, 1H), 1.31 – 1.52 (m, 4H), 0.85 – 0.98 (m, 3H). The ^{1}H NMR spectrum of this product was consistent to that in precedent literature[5].

Synthesis of L-Norvaline (L-Nva) NCA monomer

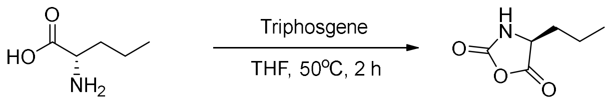

L-Norvaline (L-Nva) NCA monomer was synthesized by following the precedent literature with modifications[1]. L-Norvaline (5.0 g, 42.7 mmol) was suspended in 60 mL anhydrous THF in a dry round-bottom flask charged with a magnetic stirring bar. Then a solution of triphosgene (5.6 g, 18.8 mmol) in anhydrous THF (20 mL) was added dropwise into the reaction flask in an ice bath under nitrogen protection. The reaction was heated to 50°C and stirred under nitrogen for 2 hours. After cooling to room temperature, the mixture was concentrated under reduced pressure and then the residue was re-dissolved in EtOAc (50 mL). The mixture was washed with cold deionization water (50 mL) and cold brine (50 mL) rapidly, and then was dried over anhydrous $MgSO_4$. The crude NCA was recrystallized thrice using dried EtOAc/hexane to afford a white crystal (3.4 g, 56% yield). ^{1}H NMR (400 MHz, $CDCl_3$) δ 6.84 (s, 1H), 4.29 – 4.39 (m, 1H), 1.73 – 1.93 (m, 2H), 1.37 – 1.59 (m, 2H), 0.99 (t, J = 7.3 Hz, 3H). The ^{1}H NMR spectrum of this product was consistent to that in precedent literature[6].

Synthesis of 8-butyl-1,8-diazabicyclo[5.4.0]undec-7-enium chloride (DBUCl).

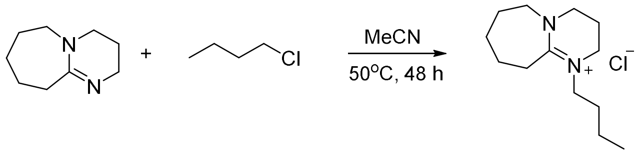

The chloride salt 8-butyl-1,8-diazabicyclo[5.4.0]undec-7-enium chloride (DBUCl) was prepared according to a the procedure in literature with modifications[7]. 1,8-

Diazabicyclo[5.4.0]undecane-7-ene (5.0 g, 32.8 mmol) was dissolved in 50 mL MeCN in a dry round-bottom flask charged with a magnetic stirring bar. Then a solution of 1-chlorobutane (6.0 g, 65.7 mmol) in MeCN (10 mL) was added dropwise into the reaction flask. The reaction was heated to 50°C and stirred in dark for 24 h to give the crude product, which was purified through silica gel column chromatography to afford DBUCl as a yellow oil (3.4g, 43% yield). ^{1}H NMR (400 MHz, $CDCl_3$) δ 3.74 – 3.81 (m, 2H), 3.67 – 3.74 (m, 2H), 3.61 – 3.66 (m, 2H), 3.49 – 3.56 (m, 2H), 2.91 – 2.97 (m, 2H), 2.11 – 2.19 (m, 2H), 1.73 – 1.84 (m, 6H), 1.55 – 1.64 (m, 2H), 1.28 – 1.40 (m, 2H), 0.93 (t, J = 7.3 Hz, 3H). ^{13}C NMR (150MHz, Methanol-d_4) δ 167.90, 55.89, 54.73, 50.16, 48.21, 31.77, 29.55, 28.96, 27.06, 24.24, 21.11, 20.69, 14.08. HREI-MS: m/z calculated for $C_{13}H_{25}N_2Cl$ $[M]^+$: 209.2012; Found: 209.2019.

Synthesis of catalyst 5-butyl-7-methyl-1,5,7-triazabicyclo[4.4.0]dec-5-enium chloride (MTBDCl).

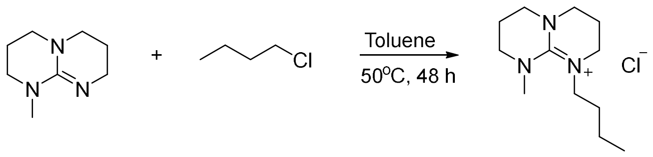

The chloride salt MTBDCl was prepared according to the procedure in literature with modifications[7]. 1-methyl-1,3,4,6,7,8-hexahydro-2H-pyrimido[1,2-a]pyrimidine (1.0 g, 6.5 mmol) was dissolved in 5 mL toluene in a dry round-bottom flask charged with a magnetic stirring bar. Then a solution of 1-chlorobutane (3.0 g, 32.6 mmol) in toluene (5 mL) was added dropwise into the reaction flask. The reaction was heated to 50°C and stirred in dark for 48 h to give the crude product, which was washed with ether and CH_2Cl_2 to afford a pale yellow oil (0.6g, 38% yield). ^{1}H NMR (400 MHz, D_2O) δ 3.34 – 3.42 (m, 4H), 3.16 – 3.28 (m, 6H), 2.98 (s, 3H), 1.95 – 2.06 (m, 4H), 1.61 – 1.71 (m, 2H), 1.28 – 1.36 (m, 2H), 0.89 – 0.95 (m, 3H). ^{13}C NMR (150 MHz, MeCN-d_3) δ 160.45, 52.82, 49.10, 48.97, 48.64, 45.20, 41.40, 30.16, 21.87, 21.74, 20.51, 13.93, HREI-MS: m/z calculated for $C_{15}H_9F_9NCl$ $[M]^+$: 210.1965; Found: 210.1972.

Synthesis of catalyst 4-dimethylamino-1-butylpyridinium chloride chloride (DMAPBCl).

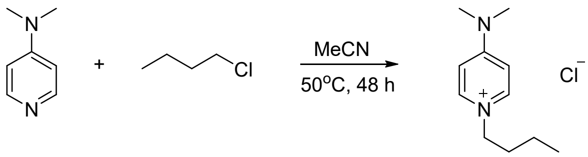

The chloride salt DMAPBCl was prepared according to the procedure in literature with modifications[7]. 4-dimethylaminopyridine (1.0 g, 8.2 mmol) was dissolved in 5 mL MeCN in a dry round-bottom flask charged with a magnetic stirring bar. Then a solution of 1-chlorobutane (1.5 g, 16.4 mmol) in MeCN (5 mL) was added dropwise into the reaction flask. The reaction was heated to 50°C and stirred in dark for 48 h to give the crude product, which was purified through silica gel column chromatography to afford DMAPBCl as a white solid (1.1g, 62% yield). ^{1}H NMR (400 MHz, $CDCl_3$) δ 8.50 – 8.62 (m, 2H), 6.97 – 7.08 (m, 2H), 4.35 (t, J = 7.3 Hz, 2H), 3.25 (s, 6H), 1.79 – 1.90 (m, 2H), 1.27 – 1.40 (m, 2H), 0.91 (t, J = 7.3 Hz, 3H). ^{13}C NMR (150 MHz, Methanol-d_4) δ 157.89, 143.08, 108.93, 58.69, 40.30, 33.94, 20.37, 13.83. HREI-MS: m/z calculated for $C_{15}H_9F_9NCl$ $[M]^+$: 179.1543; Found: 179.1547.

General procedure for primary amine initiated BLG NCA polymerization.

The procedure of typical BLG NCA polymerization was performed as follows. The BLG NCA (26.3 mg, 0.1 mmol) was dissolved in anhydrous THF (410 μL), and then 4% equivalent of 4-dimethylamino-1-neopentylpyridinium chloride (DMAPPCl) in anhydrous CH_2Cl_2 (80 μL, 0.05 M) was added to the mixture. A THF solution of n-butylamine (10 μL, 0.1 M) was added to the mixture quickly and stirred at room temperature inside a glove box. After the polymerization was completed (monitored by TLC), the resulting polypeptide was precipitated out by adding hexane (45 mL) to the reaction solution. After centrifugated and dried under air flow, the collected solid was dissolved in THF (0.2 mL) and then precipitated out again by adding hexane (45 mL) into the solution. This dissolution-precipitation procedure was repeated two more times. The collected precipitate was washed with MeOH or MeCN to remove DMAPPCl completely, then residue solid was collected and dried under vacuum to provide polypeptides (78−93% yield). For the synthesis of high molecular weight polypeptides (M/I = 200 and 500), polymerization was conducted by following the above procedure with $[M]_0$ = 1

M. It is worthy to mention that CH_2Cl_2 solvent added into DMAPPCl in above operation does not influence the rate and controllability of NCA polymerization (Table S3).

Homopolymerization of BLG NCA, BLL NCA, cbz-L-Lys, etc. was operated by following the above operation. As for random copolymerization, the overall monomer concentration was fixed at 0.2 M. As for the block copolymerization of BLL NCA with BLG NCA, the polymerization was carried out by stepwise feeding of BLG NCA and BLL NCA. When a full conversion of each block was achieved, another NCA monomer was quickly added into the reaction system until the 5 block polypeptide was synthesized.

General procedure for DMAPPCl catalyst recycle

After the primary amine-initiated NCA polymerization was completed, the reaction mixture was centrifuged and the precipitate was collected as partial of the recycled DMAPPCl catalyst. The supernatant was then precipitated using THF/hexane to obtain the polypeptide, then collected precipitate was washed with MeOH or milli-Q water. The residual solution was collected and dried under vacuum to provide the other partial of crude DMAPPCl catalyst. The two parts of recycled DMAPPCl were combined and washed with THF three times, then dried under vacuum obtain a white solid.

General procedure for the deprotection of polypeptides with *N*-Boc protecting group

To remove the side-chain *N*-Boc protecting group of PBLL or other polypeptides, the obtained polypeptide was treated with 2 mL neat trifluoroacetic acid (TFA) at room temperature for 3 hours under gentle shaking. After TFA was removed, the obtained viscous liquid was dissolved in MeOH (0.5 mL) followed by adding cold ether (40 mL) to precipitate out a white solid. The precipitate was collected by centrifugation and dried under an airflow. This dissolution-precipitation process was repeated two more times. The dried solid was dissolved in milli-Q water and lyophilized to give the deprotected polypeptide in the form of TFA salt.

To remove the side-chain O-benzyl protecting group of PBLG, PBLG was dissolved in TFA (2 mL) followed by addition of 33 wt% solution of HBr in AcOH (1 mL). After gentle

shaking overnight at room temperature, TFA was removed under an airflow. The obtained viscous liquid was dissolved in MeOH (0.5 mL) followed by adding cold ether (40 mL) to precipitate out a white solid. The precipitate was collected by centrifugation and dried under an airflow. This dissolution-precipitation process was repeated two more times. The dried solid was dissolved in milli-Q water and adjust the pH of the solution to 8 with sodium bicarbonate, then mixture was lyophilized to give the deprotected polypeptide in the form of Na salt.

Kinetic study of the polymerization on BLG NCA

The procedure of typical kinetic study of the polymerization on BLG NCA was performed as follows. BLG NCA (26.3 mg, 0.1 mmol) and internal standard triphenylmethane (1.3 mg, 0.005 mmol) were dissolved in anhydrous THF, followed by addition of a solution of 4-dimethylamino-1-neopentylpyridinium chloride (DMAPPCl) in anhydrous CH_2Cl_2 (0.02 mmol, 0.05 M). Then a solution of n-butylamine in THF ([M]:[I]:[Cat] = 100:1:4, $[M]_0$ = 0.2 M) was added to the reaction and the reaction mixture was stirred at room temperature. The unreacted BLG NCA was quantified using HPLC in real time by calculating the relative peak area ratio between the BLG NCA and the internal standard. Reverse phase HPLC analysis used the combination of water (eluent A) and acetonitrile (eluent B) as the mobile phase. The percentage of eluent B increases from 45% to 100% within 5 minutes, followed by maintaining at 100% for 5 minutes and then decreasing to 45% within 5 minutes at a flow rate of 1 mL/min. BLG NCA was analyzed by integration of the absorbance peak at 210 nm or 254 nm. Then the rate constant for chain propagation was calculated by the equation below:

$$-\frac{\mathrm{d[M]}}{\mathrm{dt}} = k_{\mathrm{p}}[\mathrm{I}][\mathrm{M}]$$

$$\mathrm{Ln}\frac{[\mathrm{M}]_0}{[\mathrm{M}]} = \mathrm{Ln}\frac{(\mathrm{A_{BLG}}/\mathrm{A_{TPM}})_0}{(\mathrm{A_{BLG}}/\mathrm{A_{TPM}})_\mathrm{t}} = k_{\mathrm{p}}[\mathrm{I}]\mathrm{t}$$

$[M]_0$ is the initial concentration of the monomer, [M] is the residual concentration of the monomer, $(\mathrm{A_{BLG}}/\mathrm{A_{TPM}})_\mathrm{t}$ is the real-time integrated areas ratio of HPLC peaks for BLG NCA and TPM, and $(\mathrm{A_{BLG}}/\mathrm{A_{TPM}})_0$ is the initial integrated areas ratio of HPLC peaks for BLG NCA and TPM. [I] is the concentration of the initiator, k_{p} is the rate constant for chain propagation.

Circular Dichroism (CD) spectroscopy analysis

Poly-BLG at variable chain length were dissolved in hexafluoroisopropanol (HFIP) to a concentration of 0.25 mg/mL and the polymer solutions were measured repeatedly at 20 ºC in a quartz cuvette with optical path length of 0.1 cm. Mean residue ellipticity [θ] was calculated using the equation:

$$[\theta]= \frac{\theta\times M_{\text{repeating unit}}}{10\times C\times L}\ \left[\text{deg}\cdot\text{cm}^2\cdot\text{dmol}^{-1}\right]$$

with $M_{\text{repeating unit}}$ = 219.1 g/mol, C = 0.25 g/L, L = 0.1 cm.

Mechanism studies

For the mechanic study, the ^{1}H NMR spectra of *tert*-butylbenzylamine and the ^{1}H NMR spectra of a 1:1 molar ratio mixture of the DMAPPCl cationic organocatalyst and *tert*-butylbenzylamine were collected separately using CD_2Cl_2 as the solvent. The ^{13}C NMR spectra of BLG NCA and the ^{13}C NMR spectra of a 1:1 molar ratio mixture of the DMAPPCl cationic organocatalyst and BLG NCA were collected separately using CD_2Cl_2 as the solvent.

The dynamic interaction between BLG NCA, primary amine and DMAPPCl was characterized by the low temperature NMR. BLG NCA, *tert*-butylbenzylamine and DMAPPCl were dissolved in CD_2Cl_2 on dry ice and then mixed together at a molar ratio of 1:1:1. The ^{13}C NMR spectrum was measured from − 80°C to 0°C. In the ^{13}C NMR experiment, each temperature needs to be kept stable for at least 10 minutes before the data collection.

For MALDI-TOF-MS characterization, polypeptide was prepared by n-butylamine-initiated polymerization on BLG NCA catalyzed by DMAPPCl in THF at room temperature ([M]/[I]/[Cat] = 15:1:4, $[M]_0$ = 0.2 M). Then, the resulting polypeptide was precipitated out by adding hexane to the reaction. After centrifugation, the polymer was obtained as a pellet at the bottom of the tube. The obtained crude polypeptide was then subjected to another two cycles of dissolution/precipitation using THF/hexane. Then DMAPPCl was completely removed after washing with MeOH thrice. Afterward, the polypeptide was characterized by MALDI-TOF-MS using 2,5-dihydroxybenzoic acid (DHB) as a matrix and using THF to dissolve the

polypeptide.

Computational details

Density functional theory (DFT) calculations reported in this work were carried out in Gaussian 09. Revision E.01 package10[8] using the exchange-correlation functional of B3LYP with different basis sets in the presence of SMD (Solvation Model Based on Density) describing THF at 298.15 K and 1.013×10^5 Pa[9]. Grimme's D3 dispersion correction was employed to improve the van der Waals interactions[10]. Specifically, 6-31G (d, p) basis set was used for structure optimization, and the vibrational frequencies of each structure have been calculated to verify the presence of zero imaginary frequency. 6-311G (d, p) basis set was used to calculate the electronic energy of each structure and the Basis Set Superposition Error (BSSE) using the full counterpoise (CP) method. Vibrational frequencies of each structure have been calculated to verify the presence of zero and single imaginary frequency for the intermediates and transition states, respectively. The reaction pathways of all TSs were checked by intrinsic reaction coordinate (IRC) calculations to confirm that these TSs were connected to the desired intermediates. Electrostatic potential map of electrostatic interaction and Local NCI analysis[11-12] were conducted with the assistance of multiwfn[13] and VMD[14].

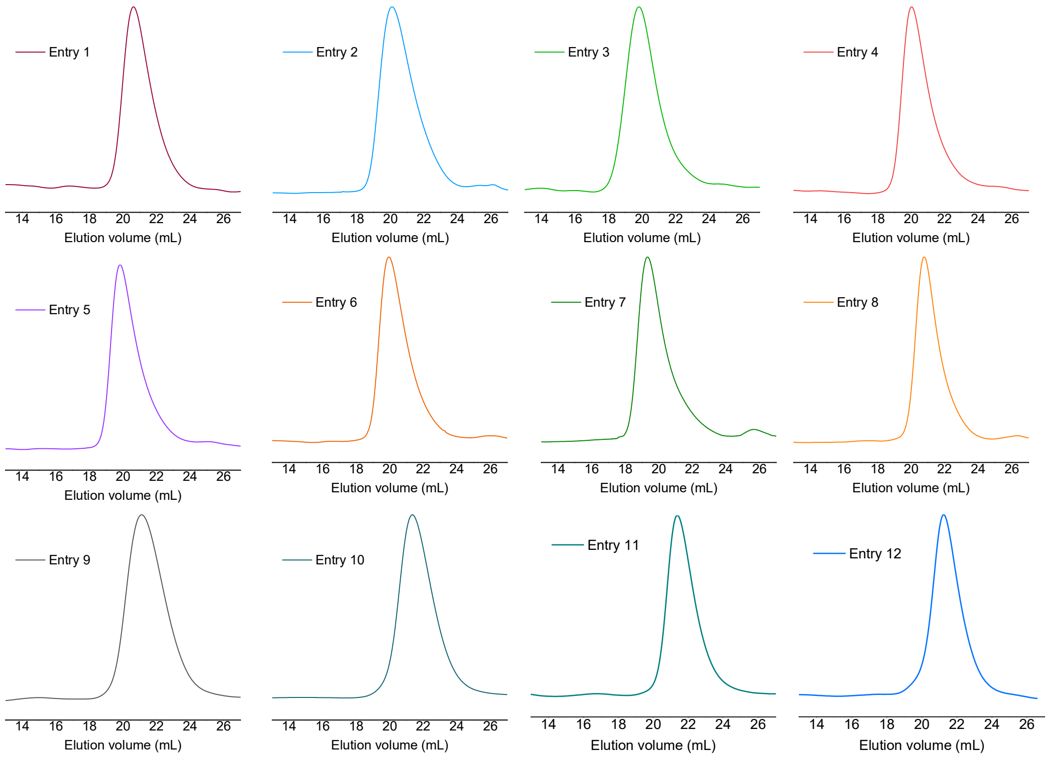

Figure S1. GPC traces of PBLG synthesized from n-butylamine-initiated ROP on BLG NCA using variable cationic catalyst, corresponding to Entry 1-10 of Table 1 in the main text.

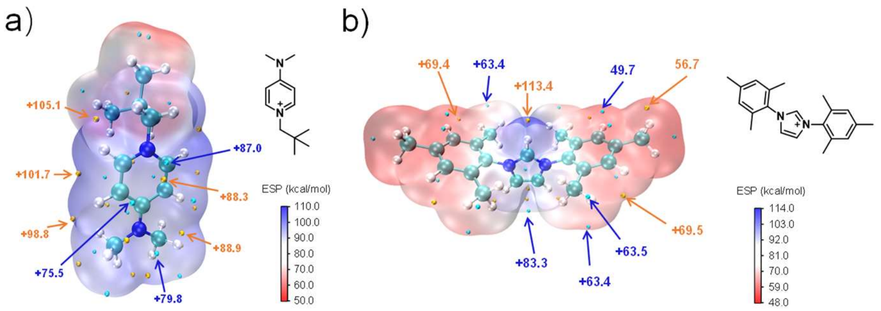

Figure S2. Electrostatic potential map of a) DMAPPCl cationic organocatalyst and b) B(TMP)MCl cationic organocatalyst. Significant surface local minima and maxima of ESP are represented as orange and cyan spheres, and labelled by blue and orange texts, respectively. The excessively conjugated structure of B(TMP)MCl indeed exhibits a lower charge density than DMAPPCl.

Table S1. The n-butylamine-initiated ROP on BLG NCA catalyzed by DMAPPCl[a].

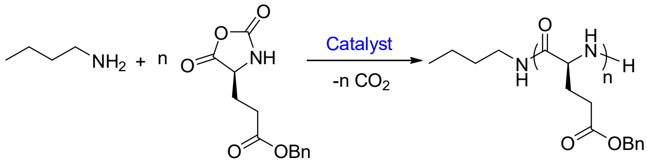

Entry	Monomer	Initiator	Catalyst	[M]:[I]:[Cat]	Time	$M_{n,calcd}$[b] (kg/mol)	$M_{n, SEC}$ (kg/mol)	$Đ$
1	BLG	n-butylamine	DMAPPCl	100/0/4	2 h	22.0	No reaction[c]	
2	BLG	n-butylamine	DMAPPCl	15/1/4	5 min	3.3	3.4	1.14

[a] The obtained polypeptide was characterized by GPC using 0.01 M LiBr in DMF as the mobile phase at a flow rate of 1 mL/min at 50°C. $M_{n, SEC}$ is the number-average molecular weight. $Đ$ is the dispersity index, dn/dc (PBLG) = 0.104 mL/g.

[b] $M_{n, calcd}$ is the theoretical number-average molecular weight.

[c] The monomer remained 99% after 2 h in the absence of primary amine initiator, while the monomer was completely consumed within 1.2 h in the presence of primary amine and DMAPPCl.

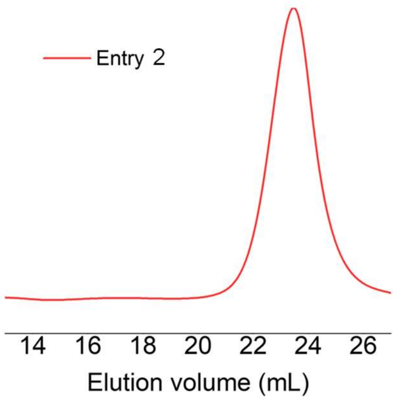

Figure S3. GPC trace of PBLG synthesized from n-butylamine-initiated ROP on BLG NCA catalyzed by DMAPPCl. Corresponding to entry 2 of Table S1.

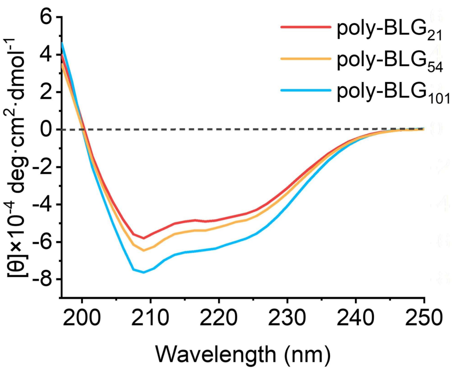

Figure S4. Circular dichroism (CD) spectra of poly-BLG at variable DP, prepared from n-butylamine-initiated ROP on BLG NCA using DMAPPCl as the catalyst

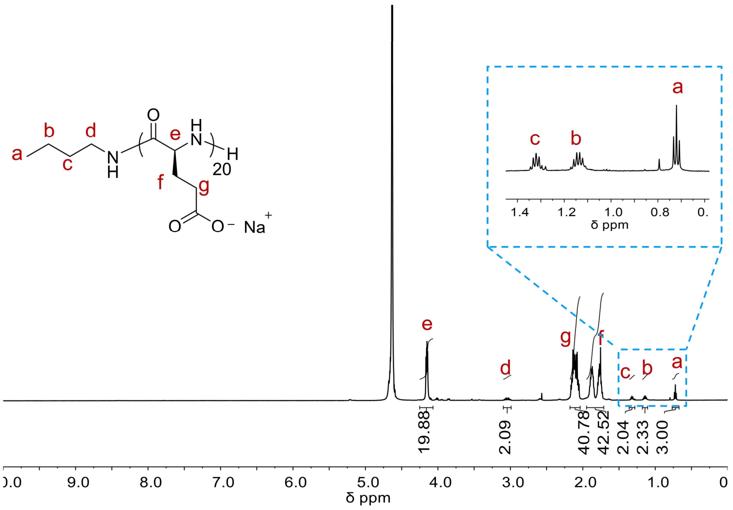

Figure S5. ^{1}H NMR spectrum of fully deprotected $PBLG_{20}$ (M]/[I]/[Cat] = 20/1/4, 600 MHz, D_2O).

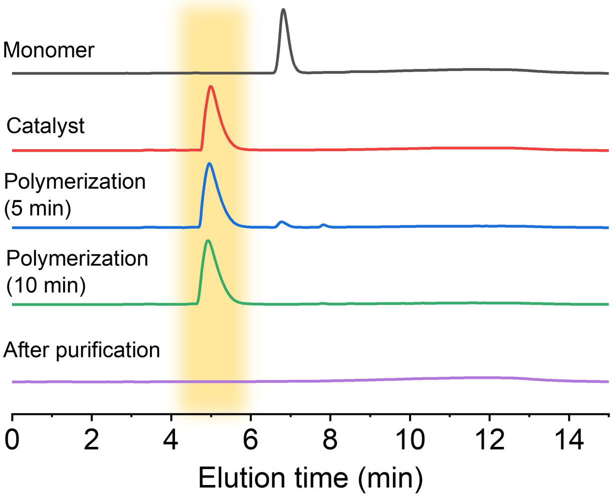

Figure S6. HPLC characterization of DMAPPCl catalyst residue in the polymer before and after purification, indicating a complete removal of DMAPPCl catalyst from the final polymer product.

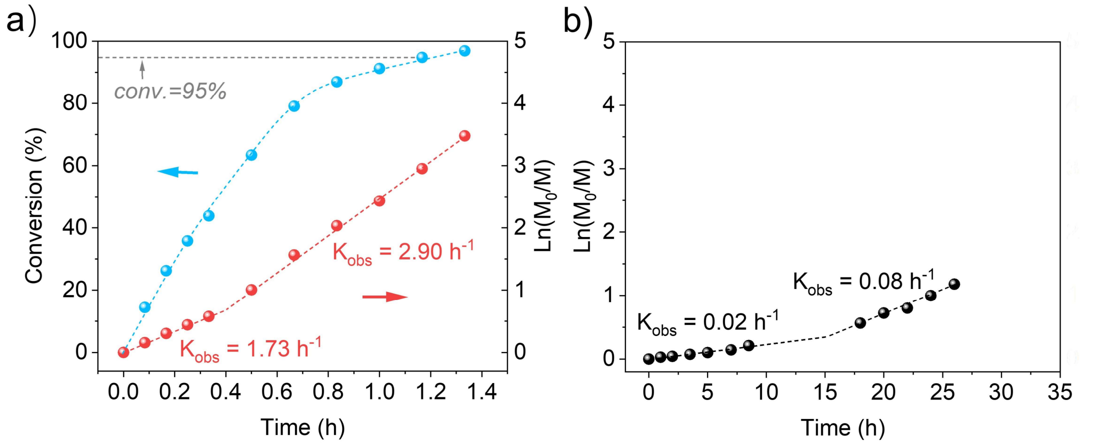

Figure S7. a) Kinetic profiles of conversion and $\ln([M]_0/[M])$ against reaction time of n-butylamine-initiated ROP on BLG NCA using DMAPPCl as the catalyst (M]/[I]/[Cat] = 100/1/4, $[M]_0$ = 0.2 M) in THF at room temperature. b) Kinetic profiles of $\ln([M]_0/[M])$ against reaction time of n-butylamine-initiated ROP on BLG NCA without catalyst (M]/[I]/[Cat] = 100/1/4, $[M]_0$ = 0.2 M) in THF at room temperature.

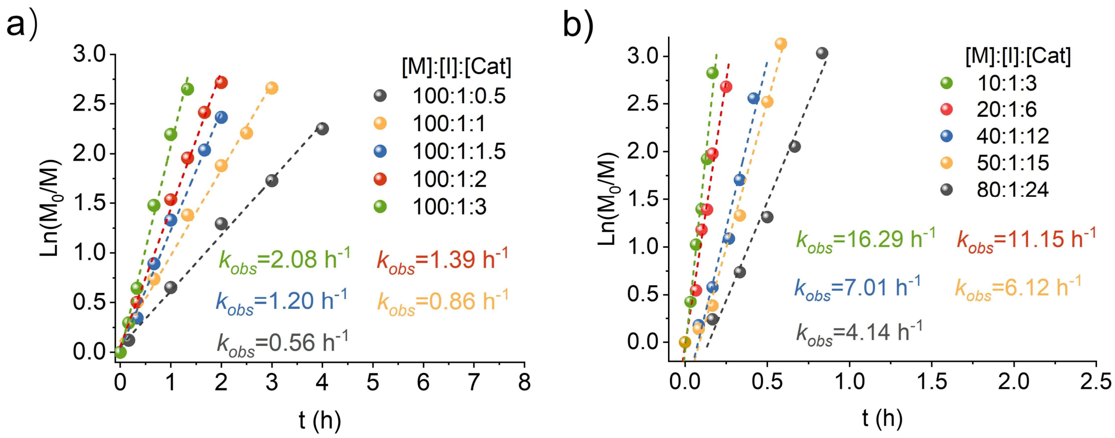

Figure S8. a) Kinetic profiles of $\ln([M]_0/[M])$ against reaction time of n-butylamine-initiated ROP on BLG NCA catalyzed by different amounts of DMAPPCl in THF at room temperature ($[M]_0$ = 0.2 M). b) Kinetic profiles of $\ln([M]_0/[M])$ against reaction time of n-butylamine-initiated ROP on BLG NCA with different [M]/[I] ratio using DMAPPCl as the catalyst in THF at room temperature ($[M]_0$ = 0.2 M).

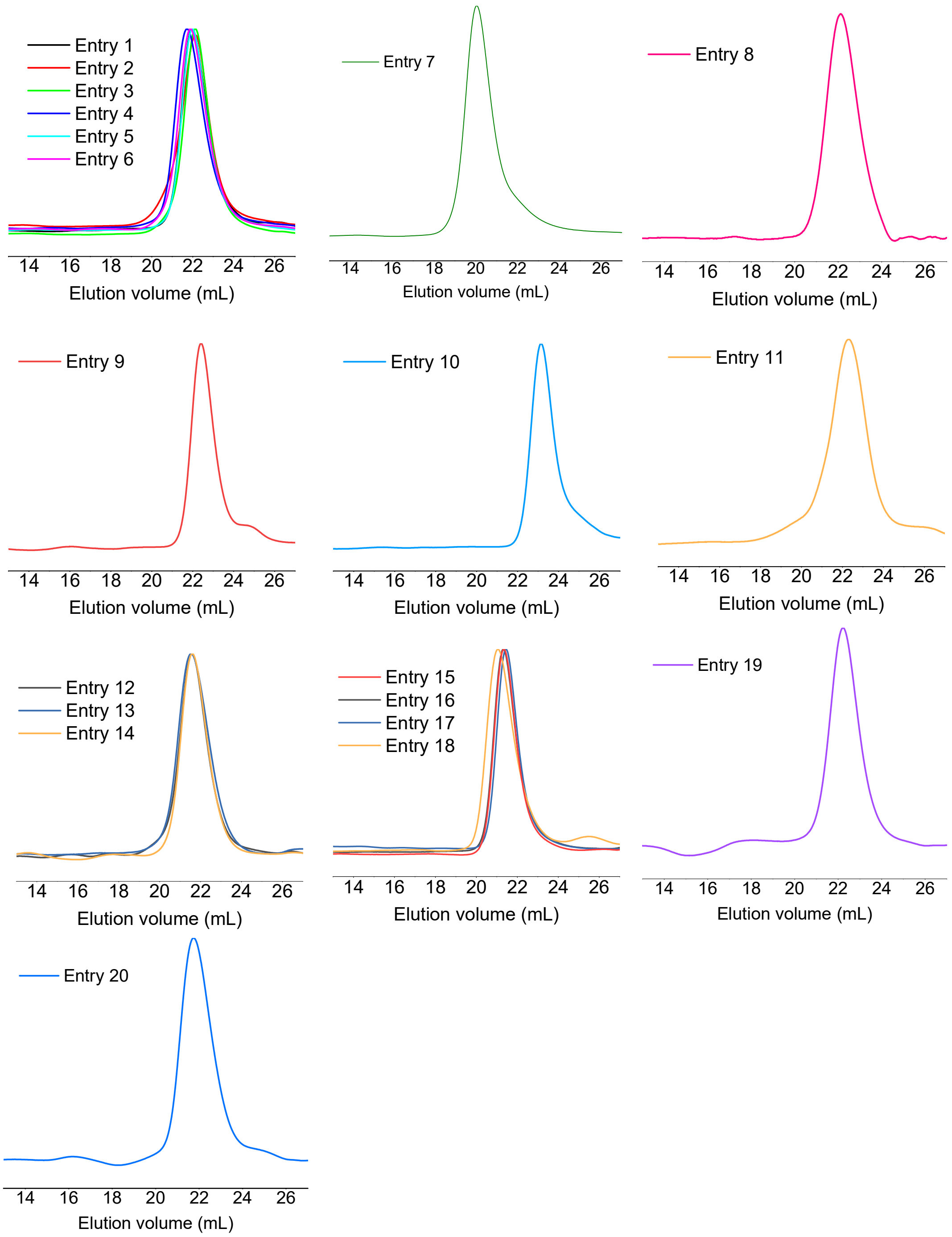

Figure S9 GPC traces of polypeptides synthesized from ROP on NCAs using different primary amine initiators in different solvents. Corresponding to Entry 1-19 of Table 2 in the main text.

Table S2. The n-butylamine-initiated ROP of BLL NCA with different [M]/[I] ratios catalyzed by DMAPPCl[a].

Entry	Solvent	Monomer	Initiator	[M]:[I]:[Cat]	Time	$M_{n,calcd}$[b] (kg/mol)	$M_{n,\ SEC}$[a] (kg/mol)	$Đ$[a]
1	THF	BLL	n-butylamine	20/1/4	15 min	4.6	5.8	1.11
2	THF	BLL	n-butylamine	50/1/4	35 min	11.5	12.8	1.22
3	THF	BLL	n-butylamine	100/1/4	1.5 h	22.9	21.6	1.20
4	THF	BLL	n-butylamine	150/1/4	4 h	34.3	30.8	1.12
5	THF	BLL	n-butylamine	200/1/4	20 h	45.6	43.5	1.10

[a] All polypeptides were characterized by GPC using 0.01 M LiBr in DMF as the mobile phase at a flow rate of 1 mL/min at 50°C. $M_{n,\ SEC}$ is the number-average molecular weight. $Đ$ is the dispersity index, dn/dc (PBLL) = 0.123 mL/g.

[b] $M_{n,\ calcd}$ is the theoretical number-average molecular weight.

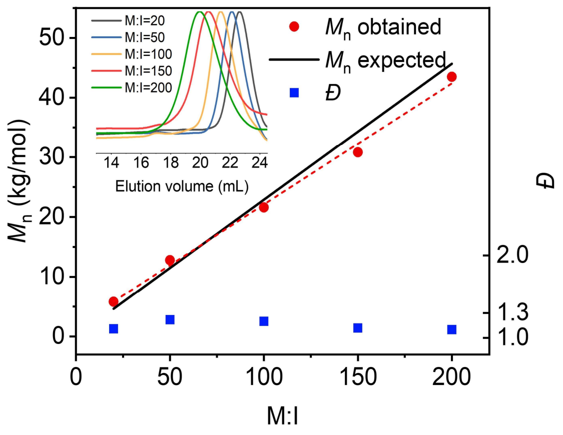

Figure S10. Plots of M_n and $Đ$ versus [M]/[I] ratio for the n-butylamine-initiated ROP of BLL NCA using DMAPPCl as the catalyst (TableS2, entries 1-5). Inset: overlay of GPC traces at different [M]/[I].

Table S3. The n-butylamine-initiated ROP of BLG NCA in mixed solvents with different CH_2Cl_2 contents.[a]

Entry	Solvent	CH_2Cl_2 content (v/v)	[M]:[I]:[Cat]	Time	$M_{n,calcd}$ (kg/mol)	$M_{n,SEC}$[b] (kg/mol)	$Đ$[b]
1	THF	20%	100/1/0	> 2 d	22.0	*N/A*[c]	*N/A*[c]
2	THF	20%	100/1/4	70 min	22.0	22.2	1.06
3	THF	10%	100/1/4	70 min	22.0	22.7	1.18

[a] All polypeptides were characterized by GPC using 0.01 M LiBr in DMF as the mobile phase at a flow rate of 1 mL/min at 50°C. $M_{n,\,SEC}$ is the number-average molecular weight. $Đ$ is the dispersity index, dn/dc (PBLG) = 0.104 mL/g.

[b] $M_{n,calcd}$ is the theoretical number-average molecular weight.

[c] *N/A* denotes that data are not collected because the reaction is not complete after 2 days according to TLC test.

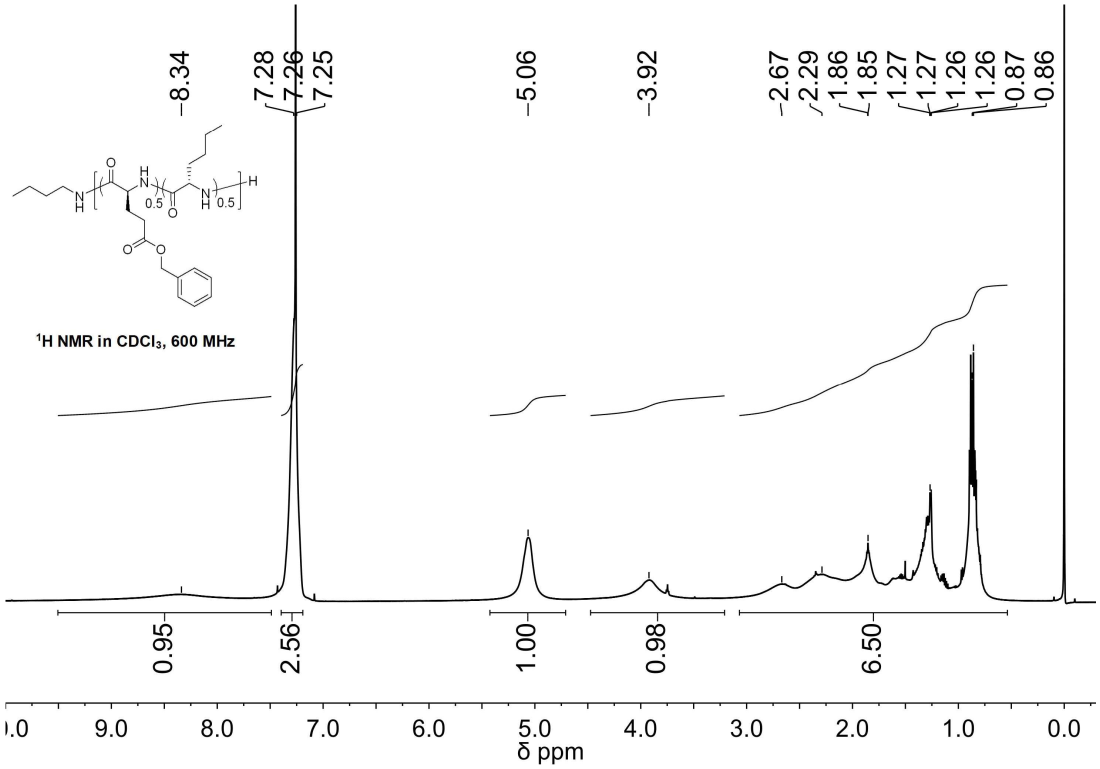

Figure S11. ^{1}H NMR for PBLG-r-PNle, corresponding to Entry 12 of Table 2 in the main text ($CDCl_3$, 600 MHz).

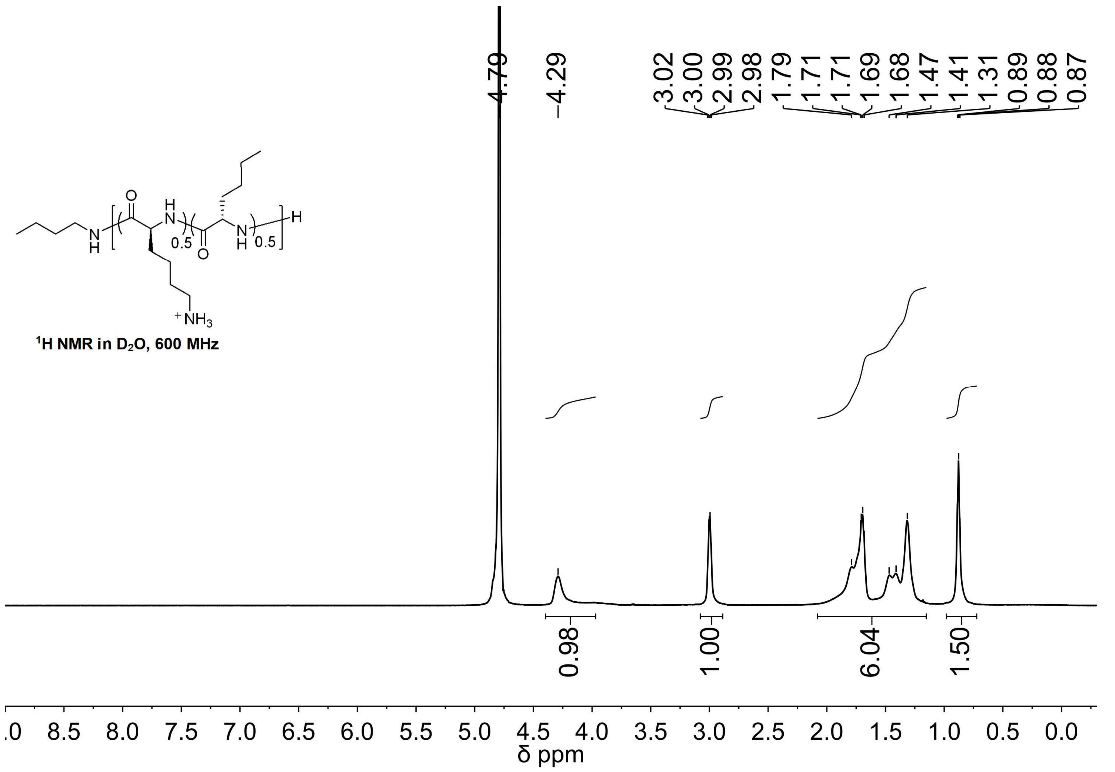

Figure S12. ^{1}H NMR for PLys-r-PNle, corresponding to Entry 13 of Table 2 in the main text (D_2O, 600 MHz).

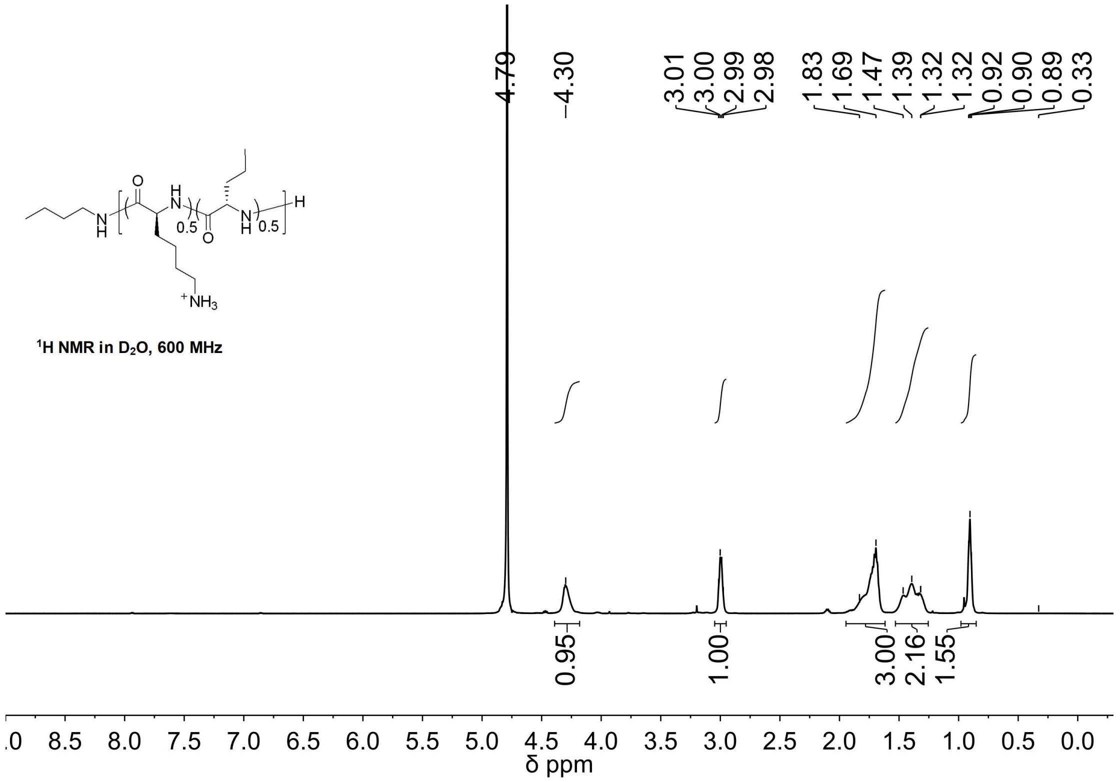

Figure S13. ^{1}H NMR for PLys-r-PNva, corresponding to Entry 14 of Table 2 in the main text (D_2O, 600 MHz).

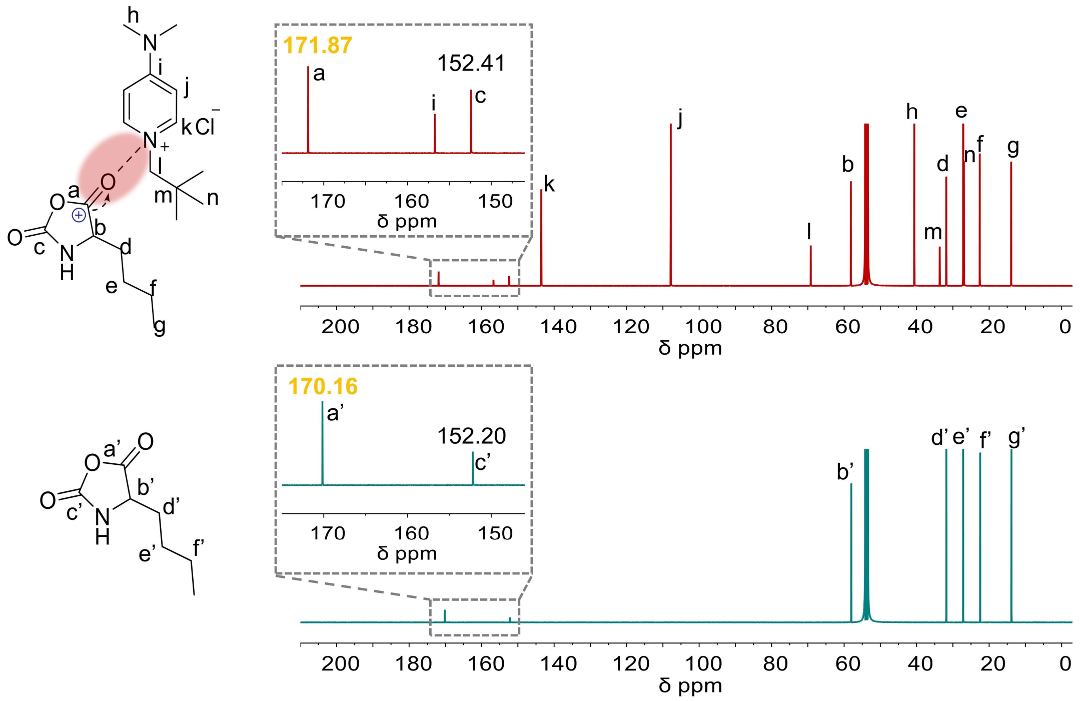

Figure S14. Comparison of full ^{13}C NMR spectra for Nle NCA only and Nle NCA mixing with DMAPPCl at a molar ratio of 1:1 in CD_2Cl_2. Corresponding to Figure 4a in the main text.

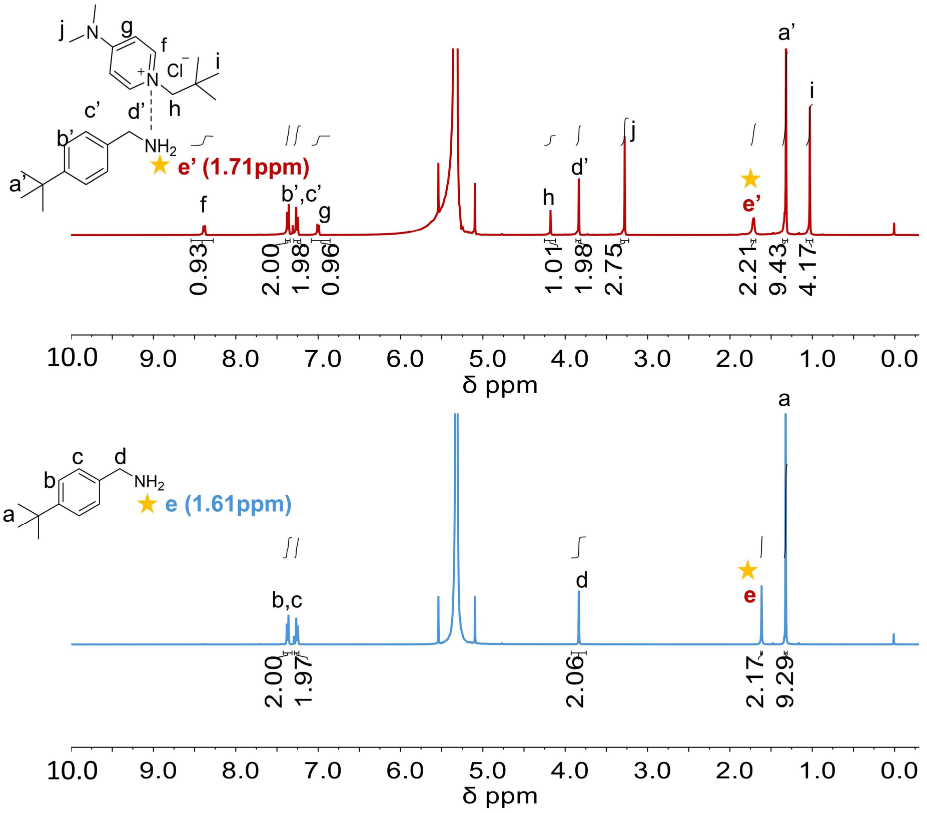

Figure S15. Comparison of full ^{1}H NMR spectra for *tert*-butylbenzylamine only and *tert*-butylbenzylamine mixing with DMAPPCl at a molar ratio of 1:1 in CD_2Cl_2. Corresponding to Figure 4b in the main text.

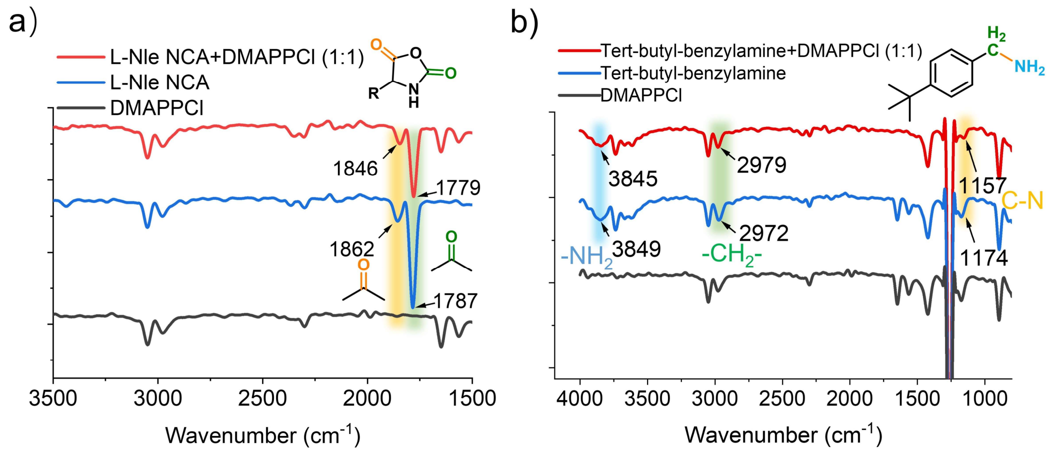

Figure S16. a) FTIR spectra for characterization of the interaction between Nle NCA and DMAPPCl at a molar ratio of 1:1 in CH_2Cl_2. b) FTIR spectra for characterization of the interaction between *tert*-butylbenzylamine and DMAPPCl at the molar ratio of 1:1 in CH_2Cl_2. The absorption peak of 1862 cm^{-1} belongs to the C5 carbonyl of Nle NCA. The absorption peak of 1787 cm^{-1} belongs to the C2 carbonyl of Nle NCA. The activation of Nle NCA by DMAPPCl causes the red shift of both C5 and C2 carbonyl peaks, whereas the shift of the C5 carbonyl peak is more than that of the C2 carbonyl peak, indicating that the catalyst has a stronger activation effect on the C5 carbonyl. The absorption peak of 3849 cm^{-1} belongs to the N-H stretching of NH_2 group. The absorption peak of 1174 cm^{-1} belongs to the C-N stretching vibration. The absorption peak of 2972 cm^{-1} belongs to the CH_2 at the α-position of NH_2 group. DMAPPCl causes a red shift of the N-H and C-N peaks, and a blue shift of the CH_2 peak, indicating that the catalyst can passivate the amino group.

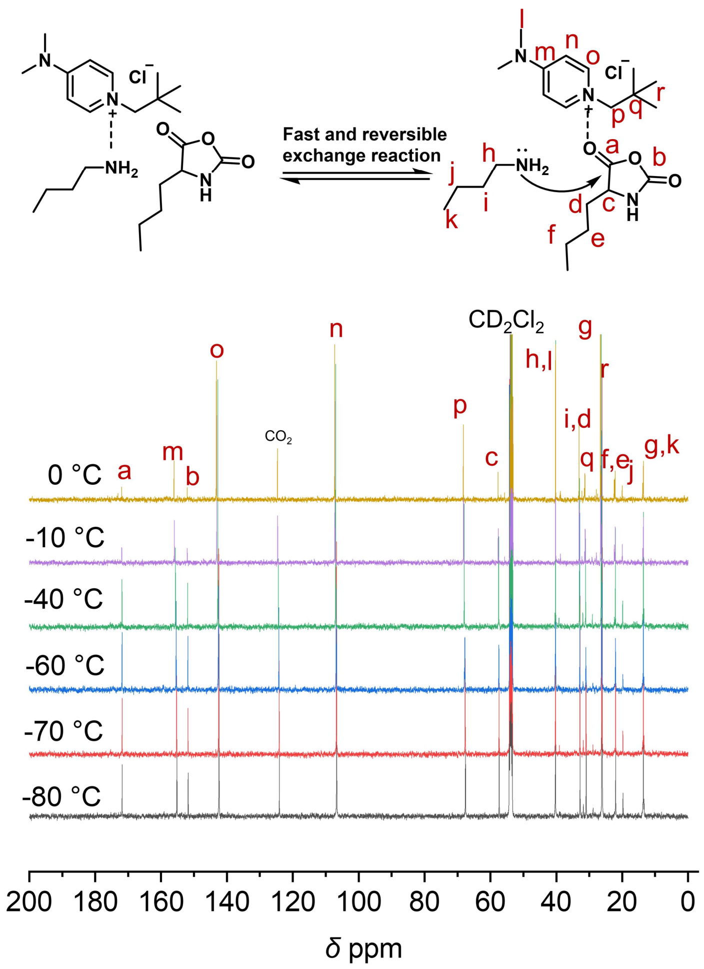

Figure S17. ^{13}C NMR spectra for a mixture of Nle NCA, n-butylamine and DMAPPCl at the molar ratio of 1:1:1 in CD_2Cl_2 at variable temperature (− 80°C ~ 0°C). Corresponding to Figure 4c in the main text.

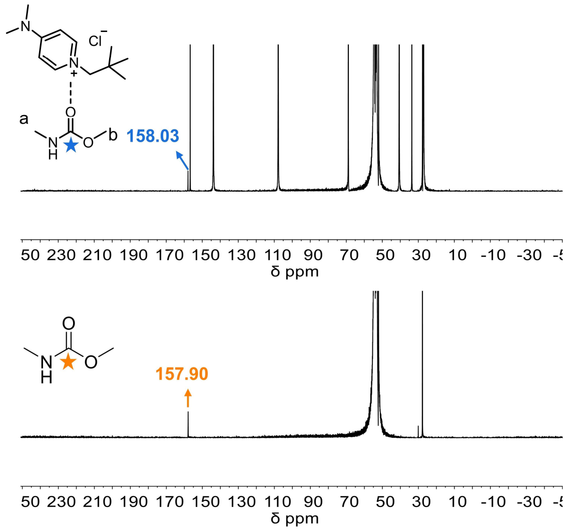

Figure S18. ^{13}C NMR spectra for methyl *N*-methylcarbamate only and methyl *N*-methylcarbamate mixing with DMAPPCl at the molar ratio of 1:1 in CD_2Cl_2. Corresponding to Figure 4d in the main text.

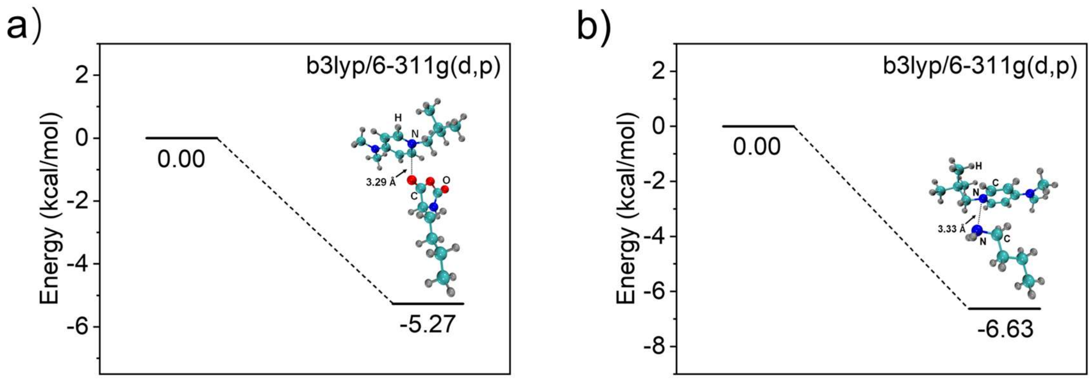

Figure S19. a) DFT calculation on the binding energy of the electrostatic interaction between the Nle NCA and DMAPPCl. b) DFT calculation on the binding energy of the electrostatic interaction between the n-butylamine and DMAPPCl.

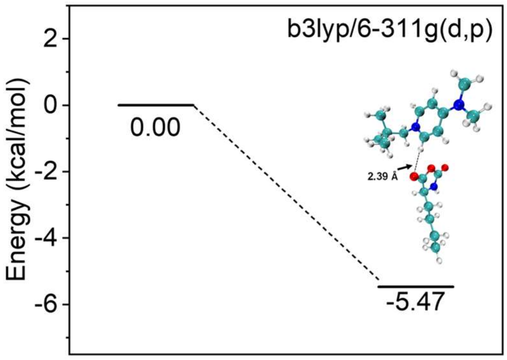

Figure S20. DFT calculation on the binding energy of the hydrogen bonding interaction between the Nle NCA and DMAPPCl.

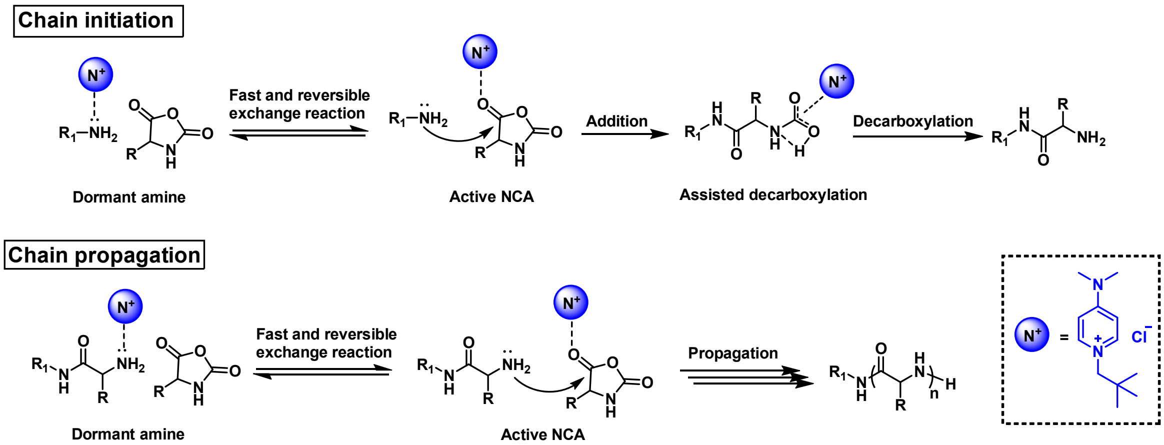

Figure S21. Proposed mechanism of the primary amine-initiated ROP of NCA, catalyzed by DMAPPCl.

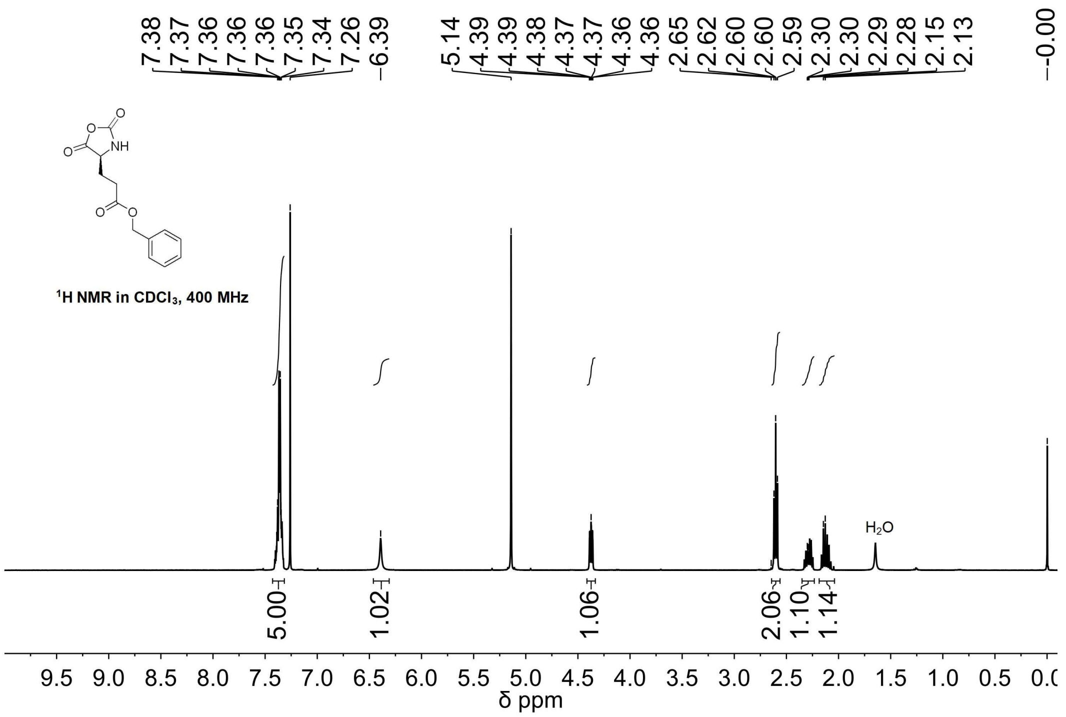

Figure S22. ^1H NMR for BLG NCA ($CDCl_3$, 400 MHz).

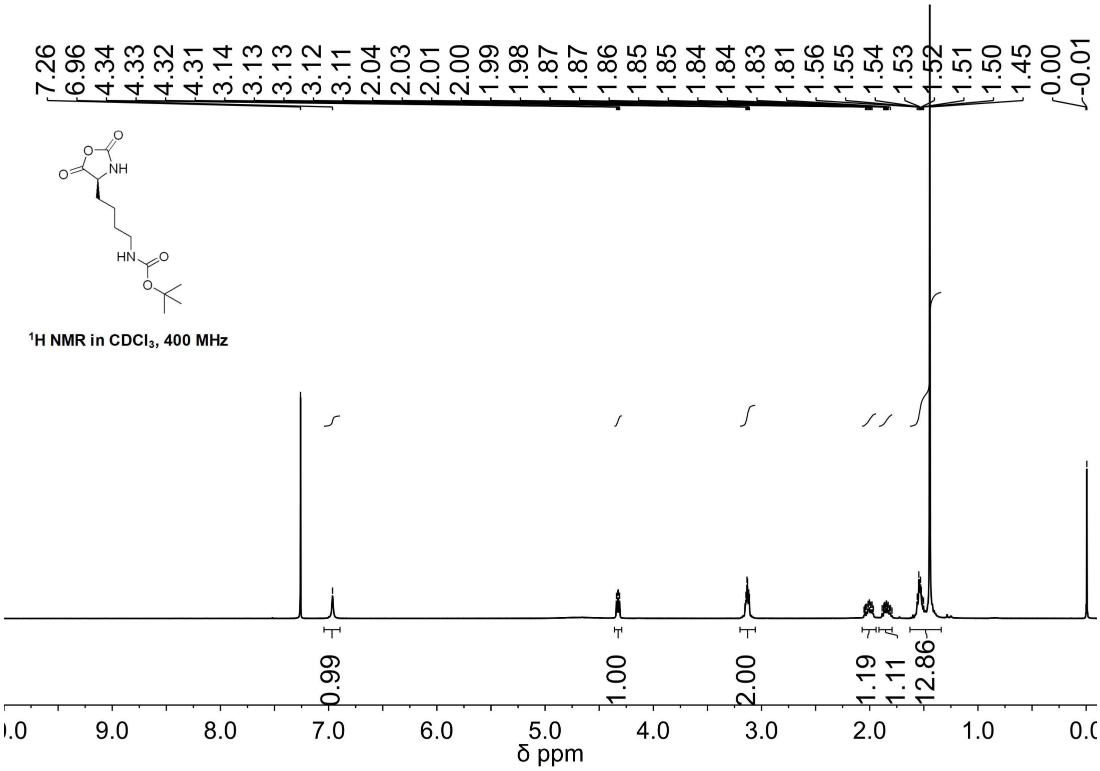

Figure S23. ^1H NMR for BLL NCA ($CDCl_3$, 400 MHz).

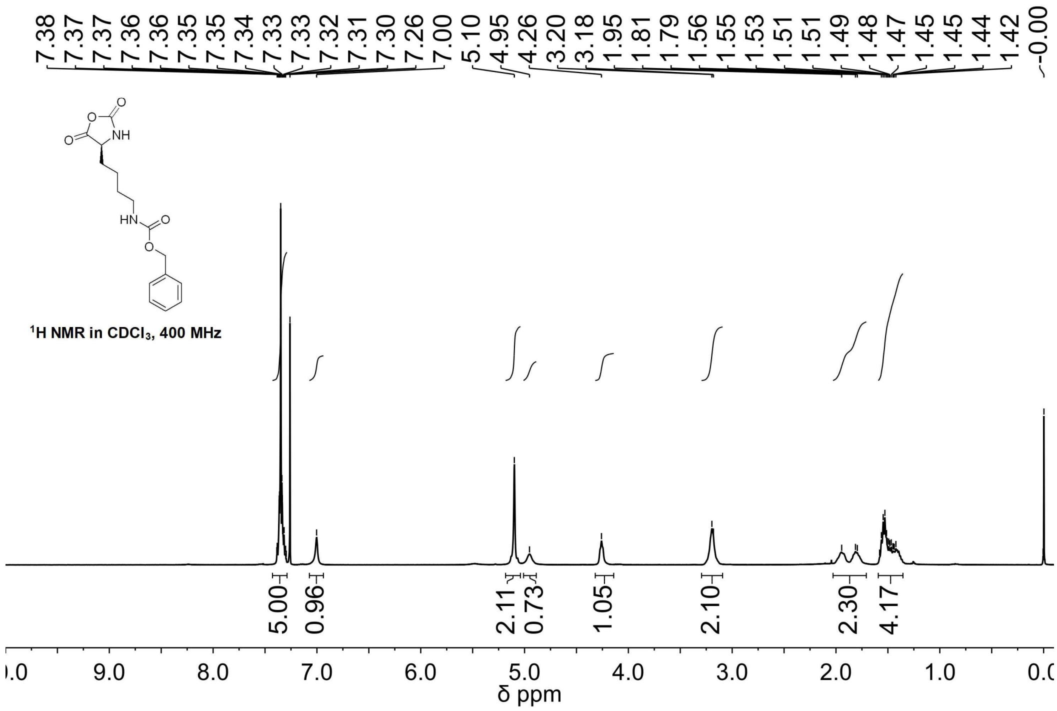

Figure S24. ^{1}H NMR for Cbz-L-Lys-NCA ($CDCl_3$, 400 MHz).

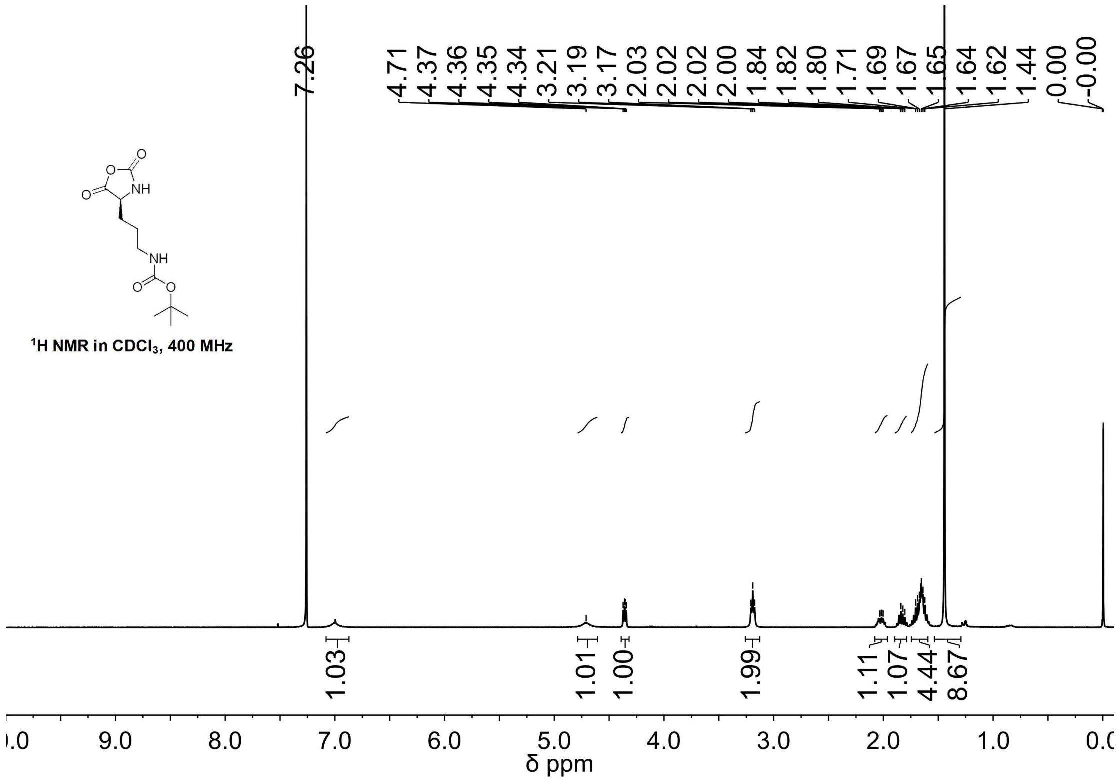

Figure S25. ^{1}H NMR for tbu-L-Orn-NCA ($CDCl_3$, 400 MHz).

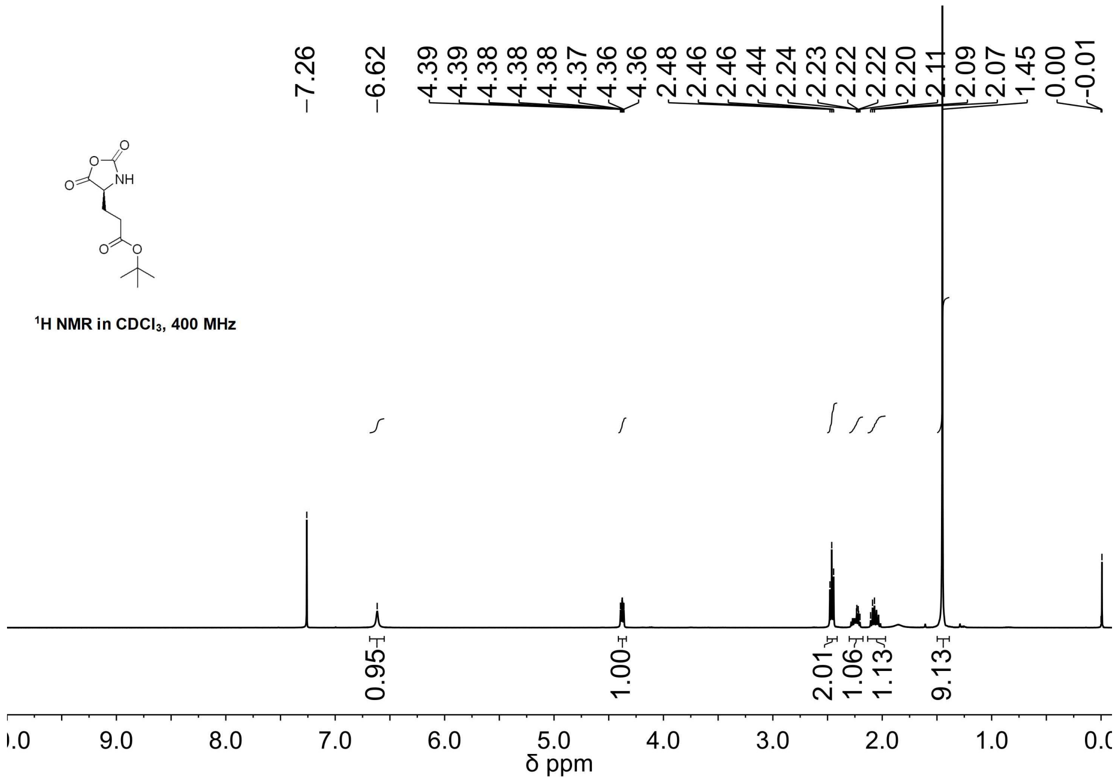

Figure S26. ^{1}H NMR for tbu-L-Glu-NCA ($CDCl_3$, 400 MHz).

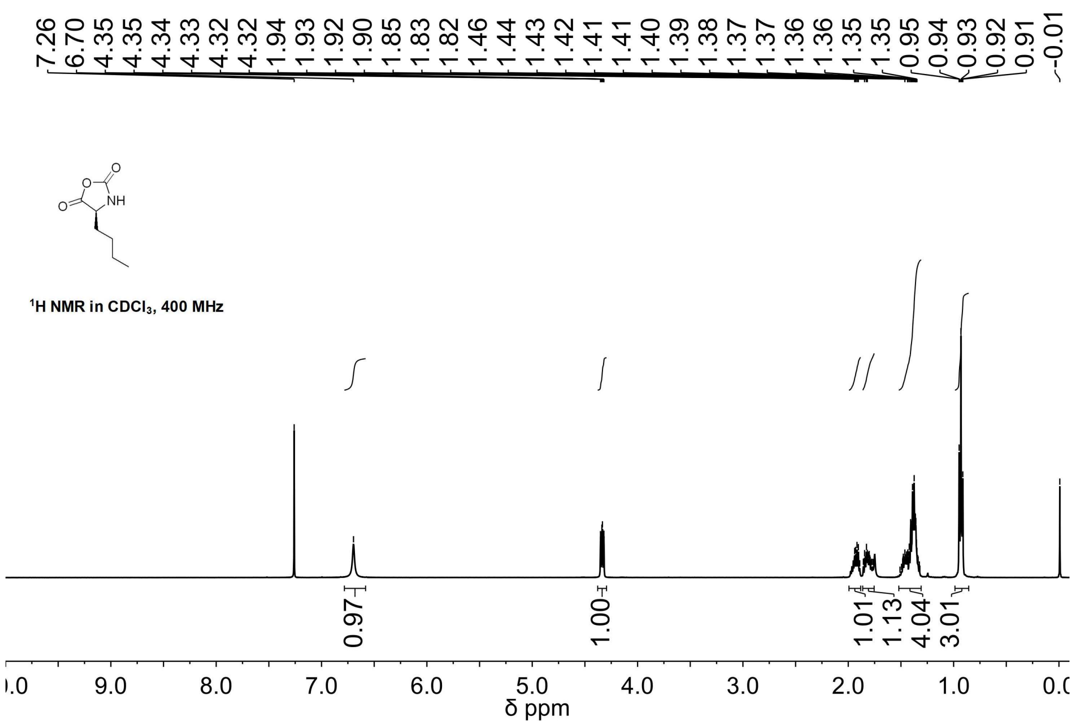

Figure S27. ^{1}H NMR for L-Nle-NCA ($CDCl_3$, 400 MHz).

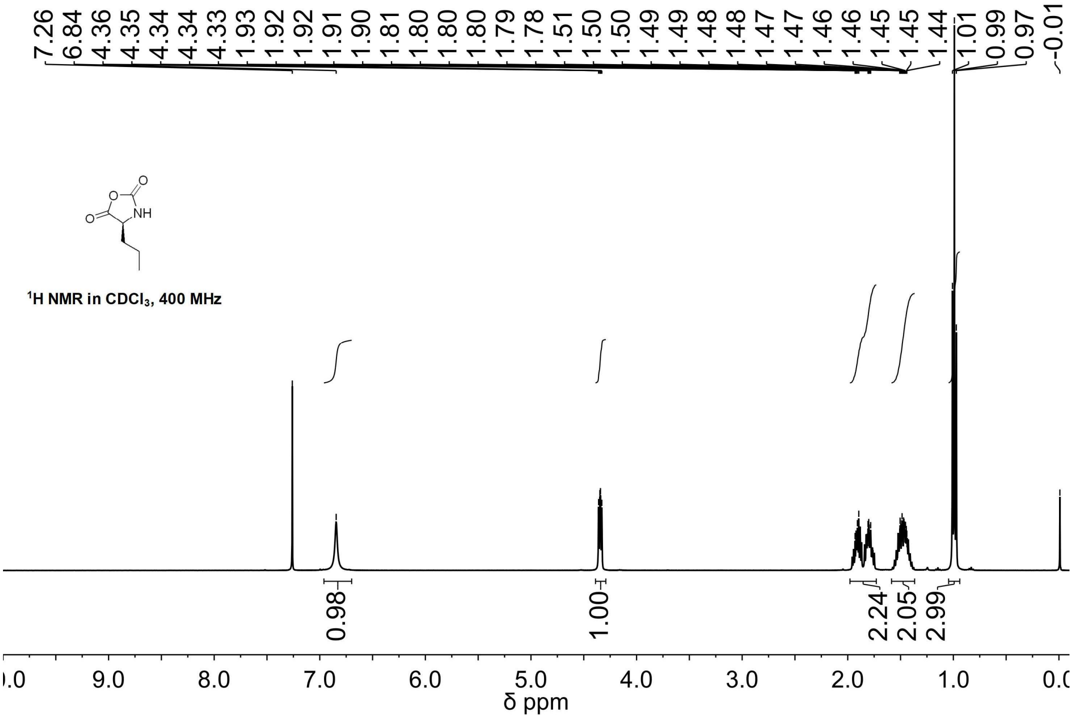

Figure S28. ^{1}H NMR for L-Nva-NCA ($CDCl_3$, 400 MHz).

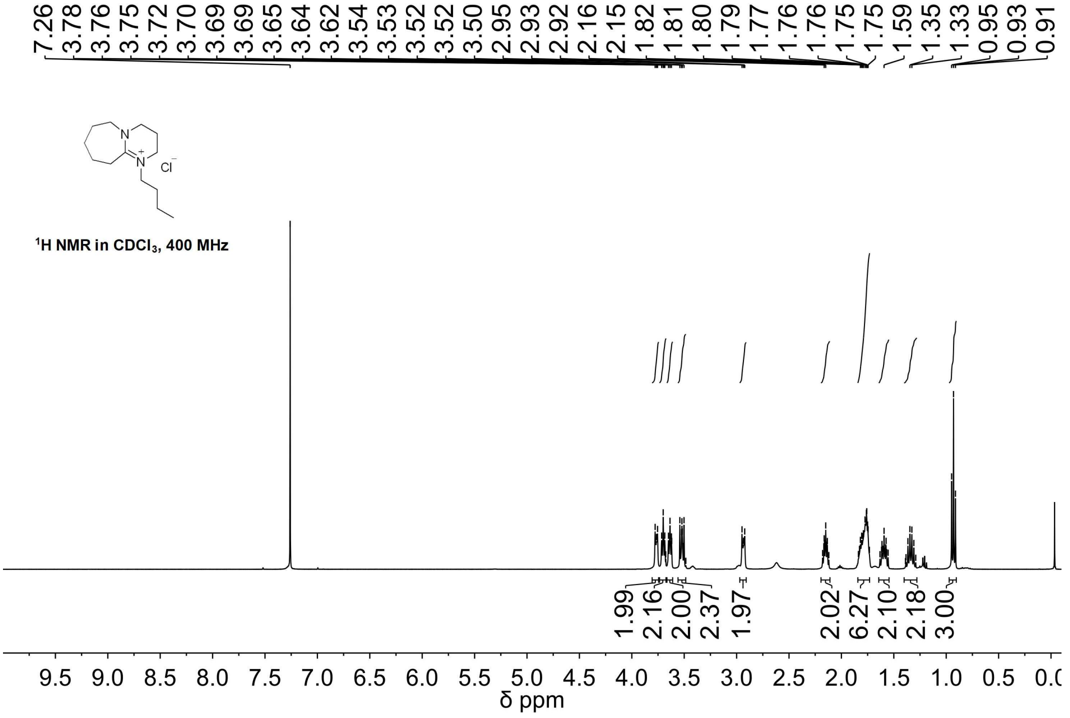

Figure S29. ^{1}H NMR for DBUCl ($CDCl_3$, 400 MHz).

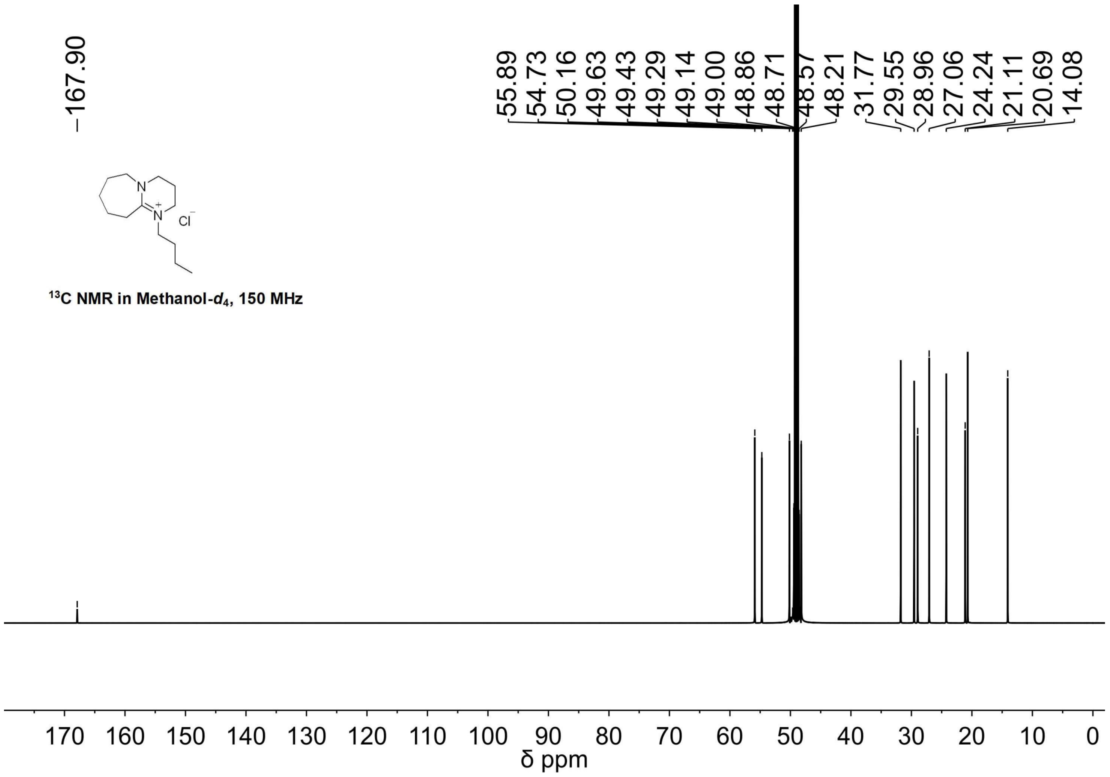

Figure S30. ^{13}C NMR for DBUCl (Methanol-d_4, 150 MHz).

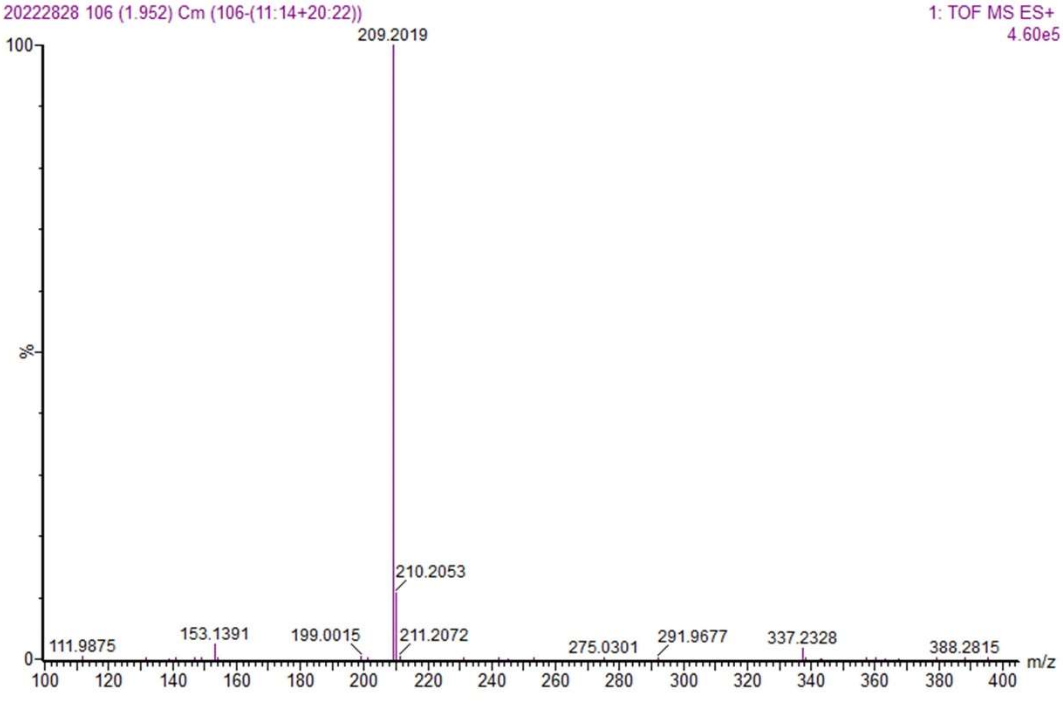

Figure S31 HREI-MS for DBUCl.

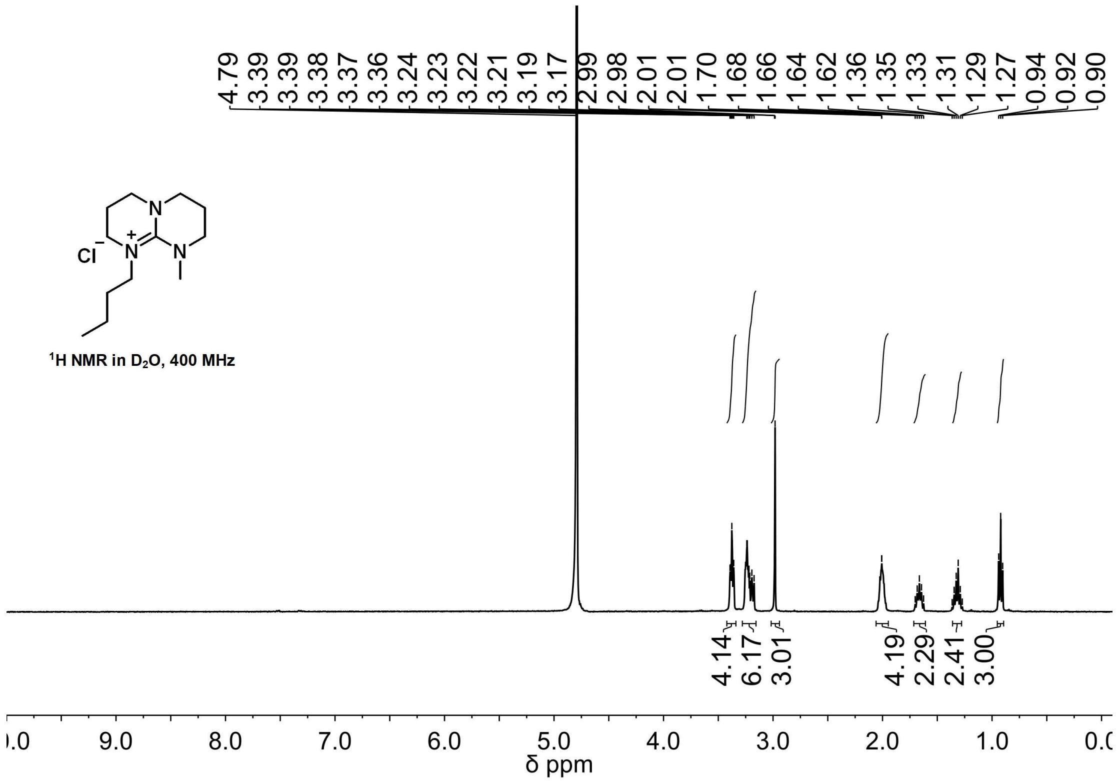

Figure S32. ^{1}H NMR for MTBDCl (D_2O, 400 MHz).

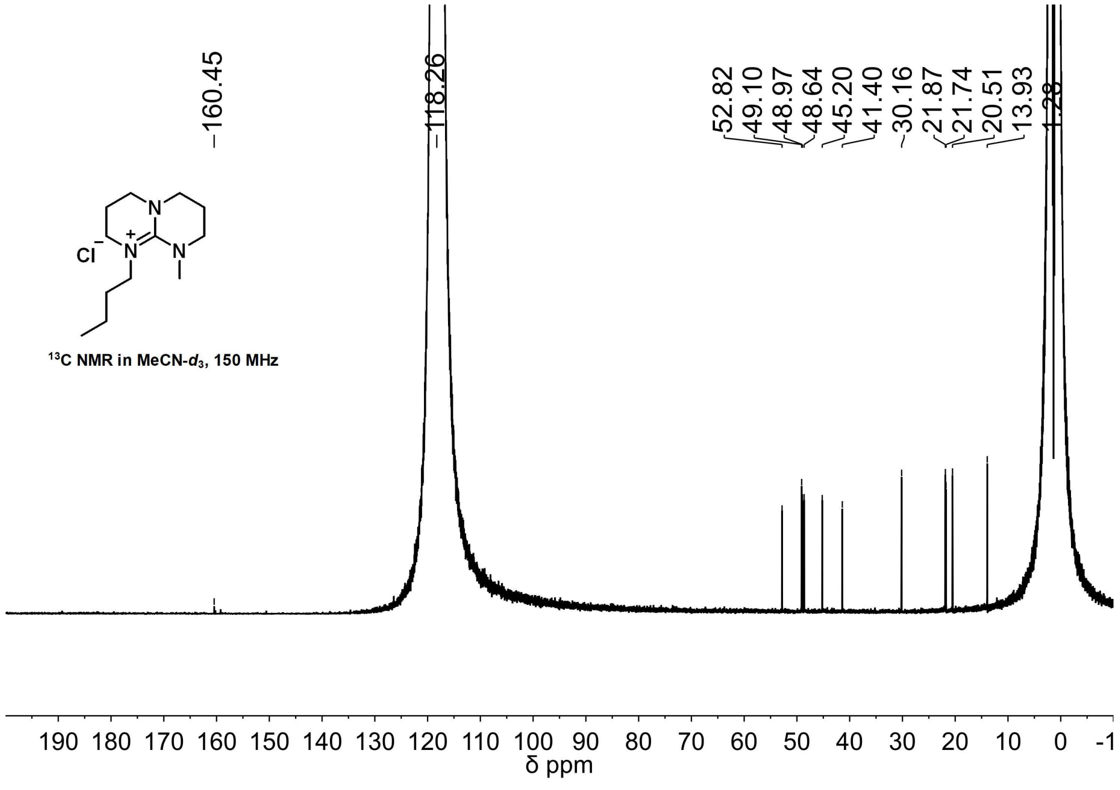

Figure S33. ^{13}C NMR for MTBDCl (MeCN-d_3, 150 MHz).

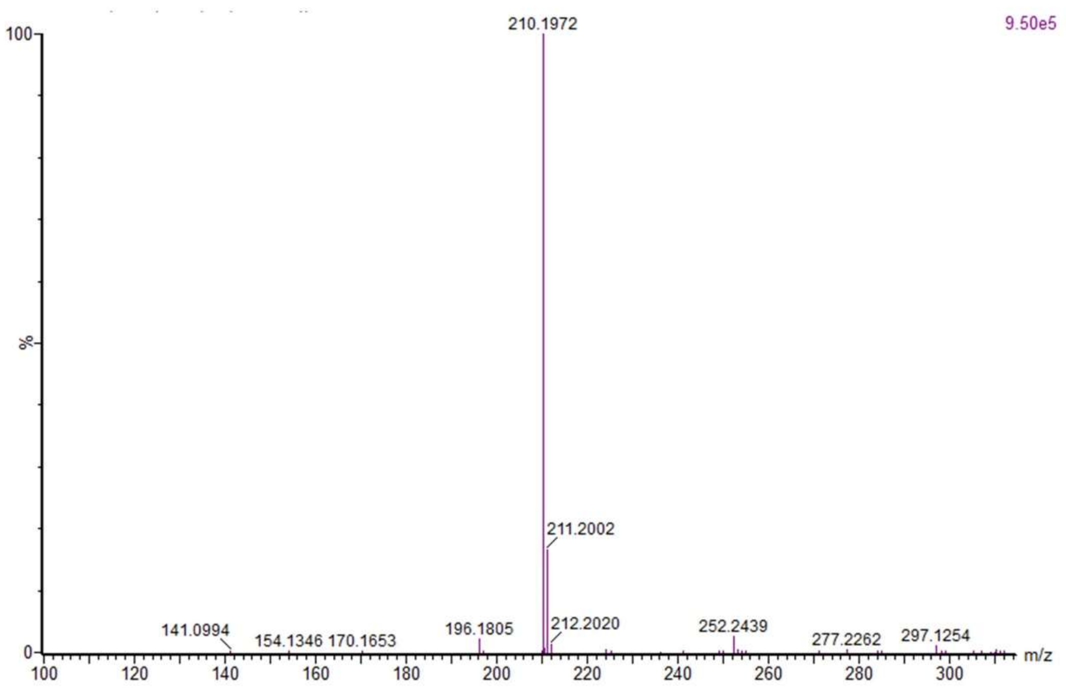

Figure S34. HREI-MS for MTBDCl.

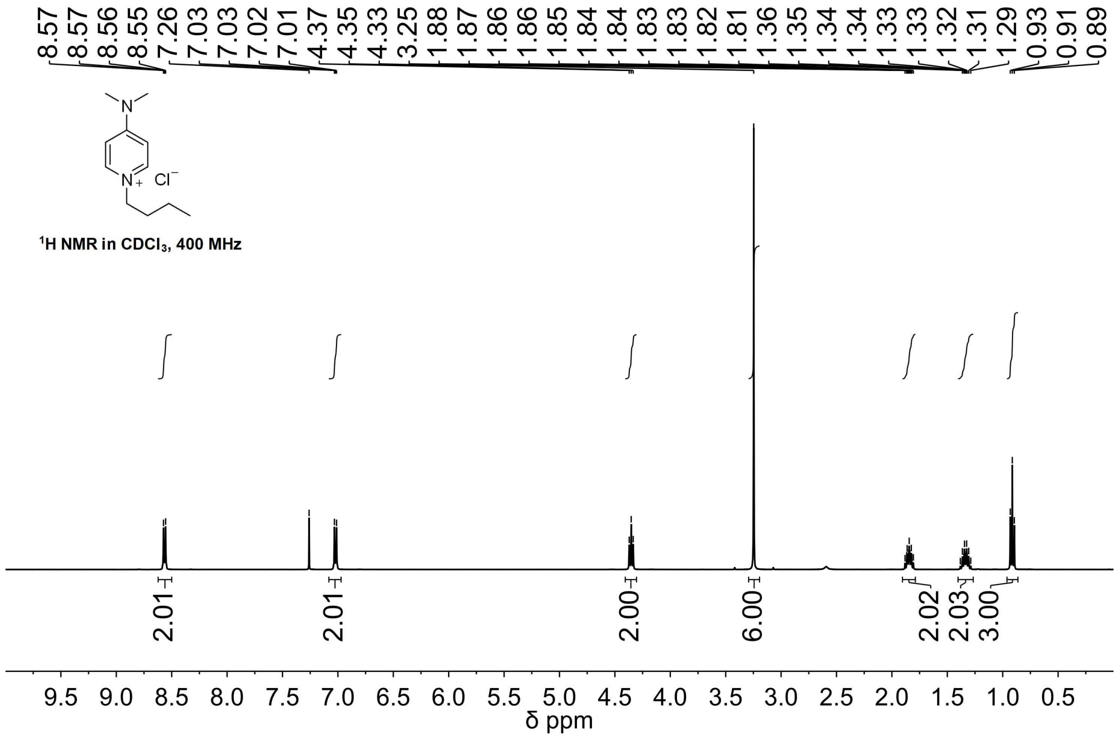

Figure S35. ^{1}H NMR for DMAPBCl ($CDCl_3$, 400 MHz).

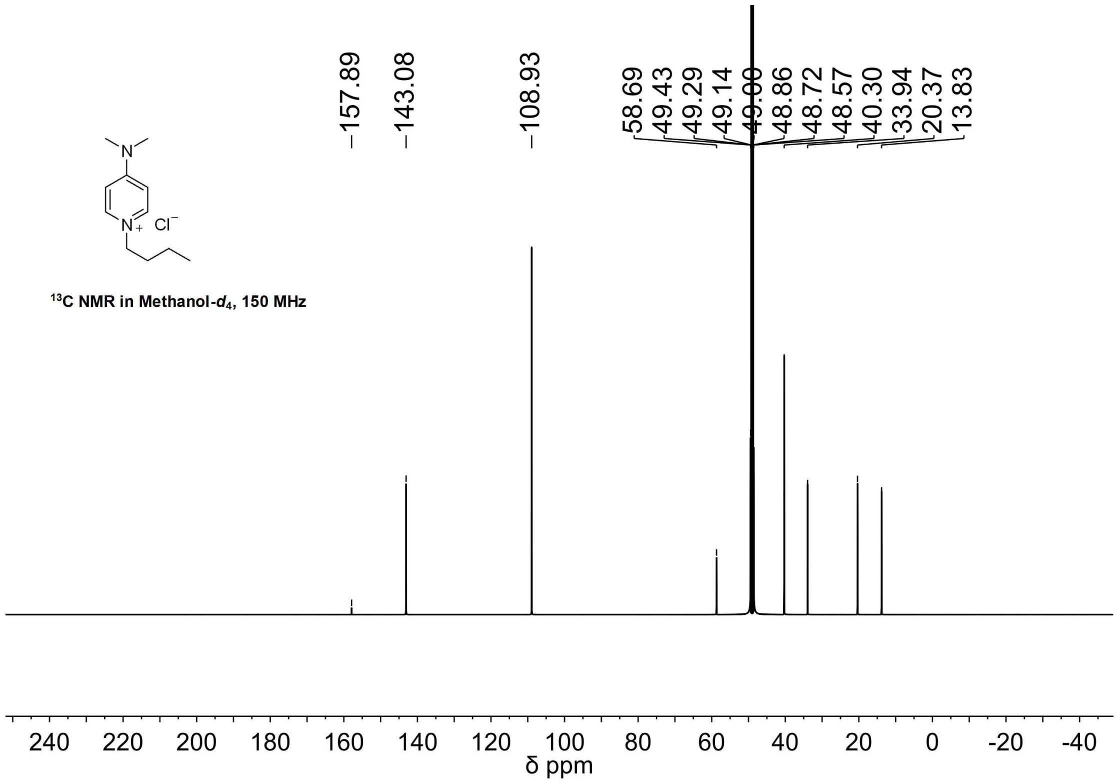

Figure S36. ^{13}C NMR for DMAPBCl (Methanol-d_4, 150 MHz).

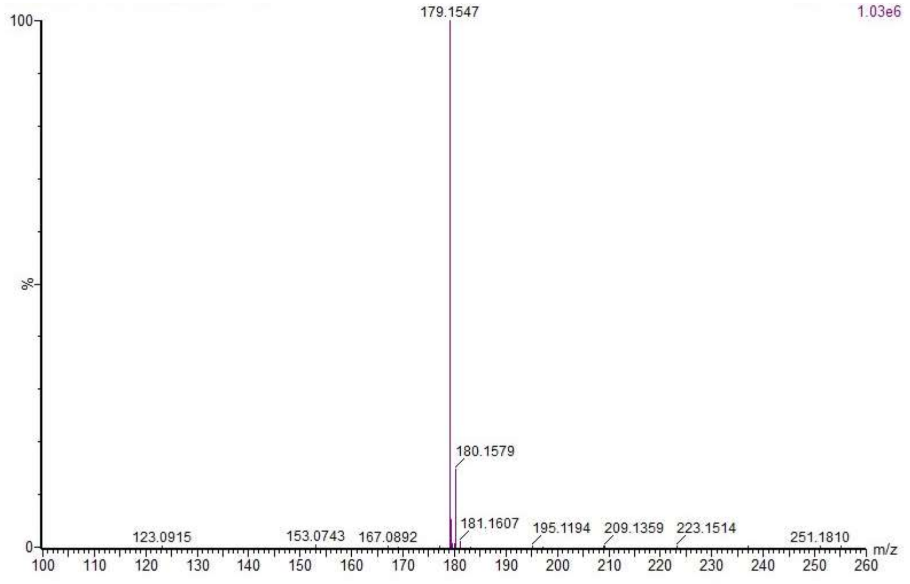

Figure S37. HREI-MS for DMAPBCl.

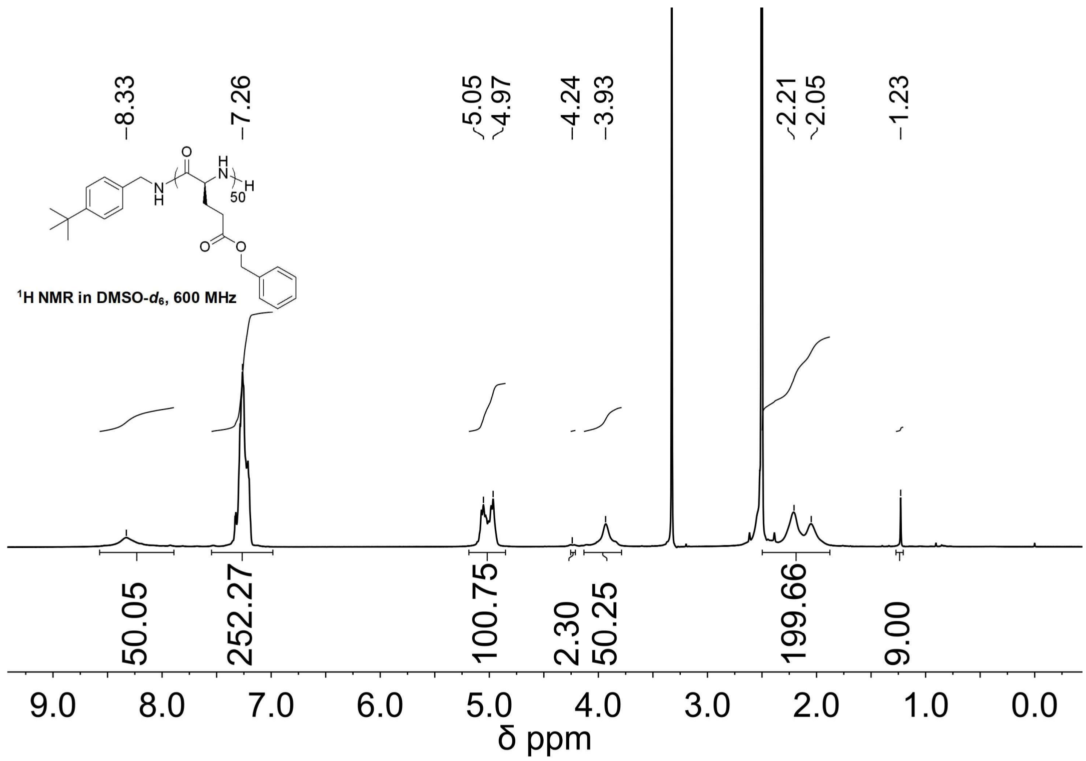

Figure S38. ^{1}H NMR for PBLG, corresponding to Entry 1 of Table 2 in the main text (DMSO-d_6, 600 MHz).

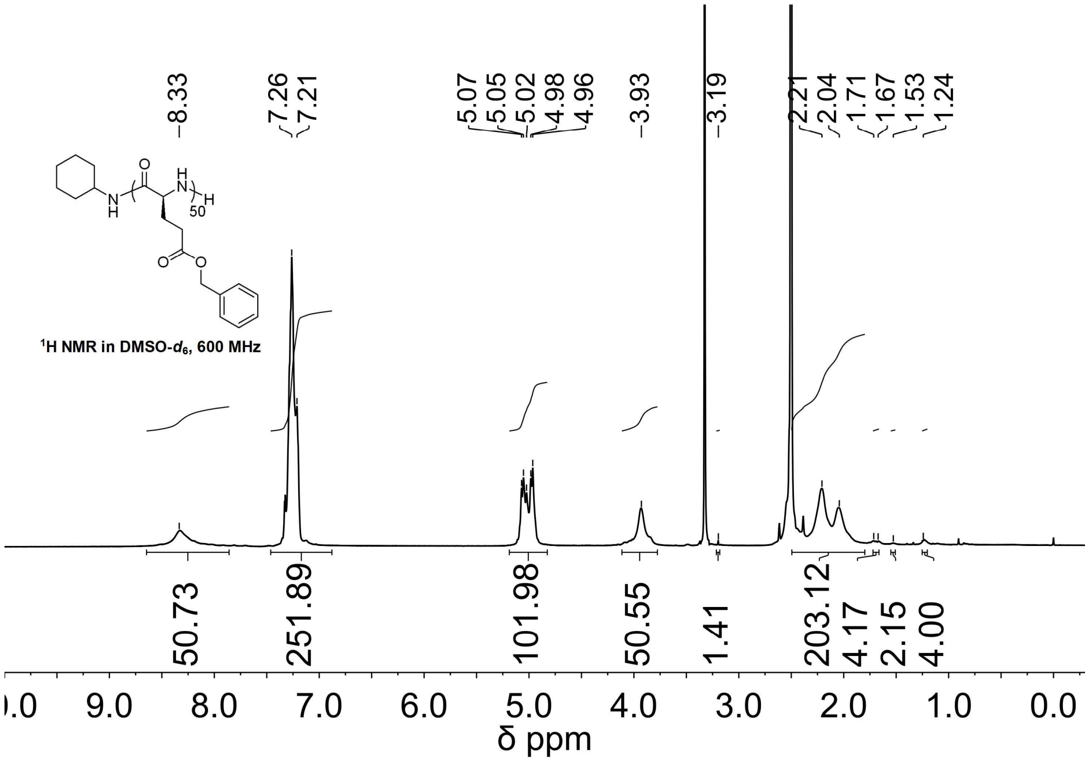

Figure S39. ^{1}H NMR for PBLG, corresponding to Entry 2 of Table 2 in the main text (DMSO-d_6, 600 MHz).

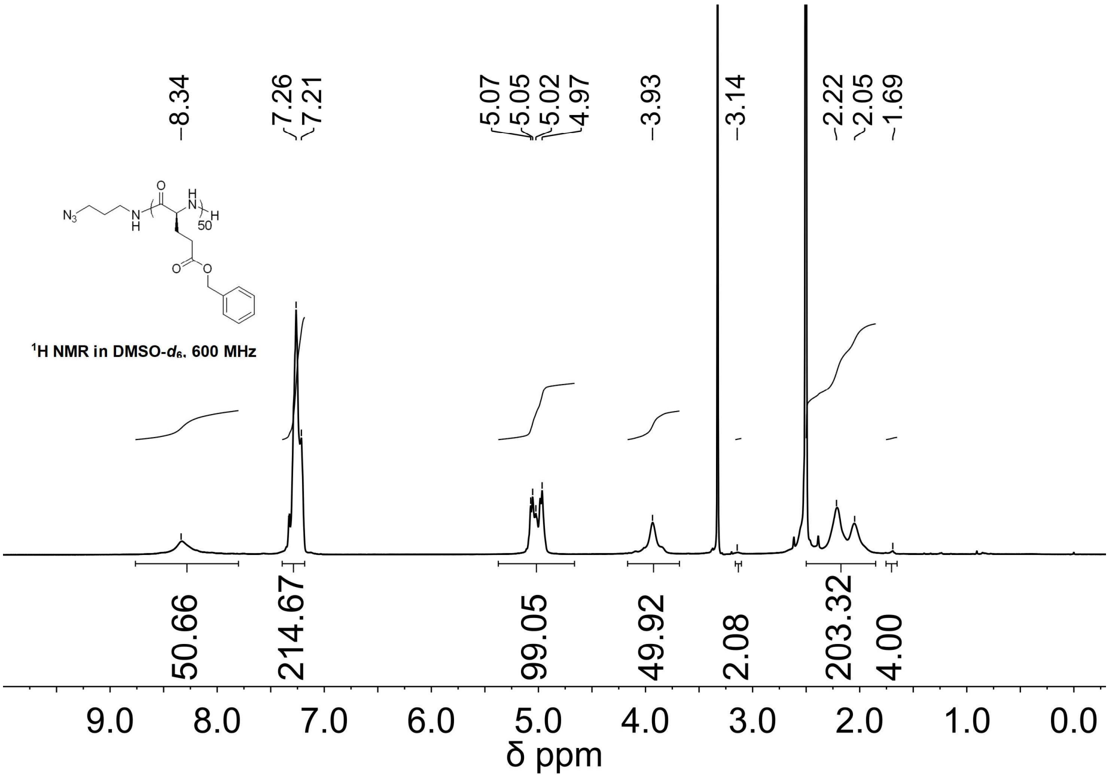

Figure S40. ^{1}H NMR for PBLG, corresponding to Entry 3 of Table 2 in the main text (DMSO-d_6, 600 MHz).

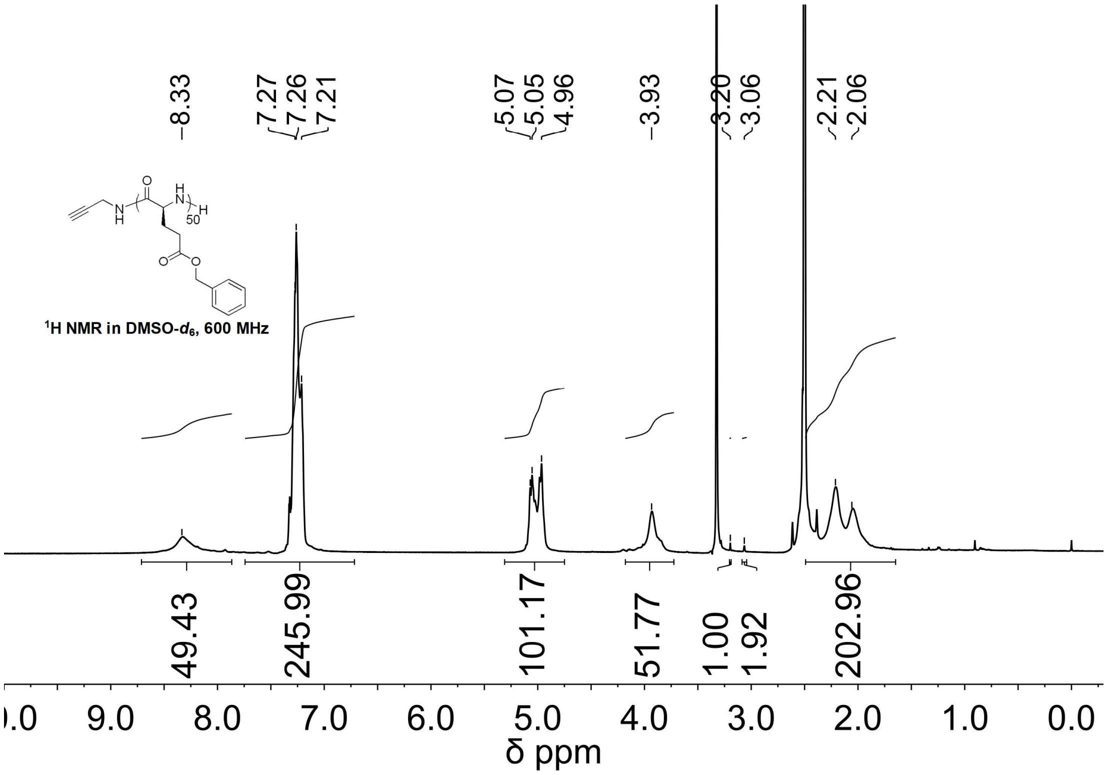

Figure S41. ^{1}H NMR for PBLG, corresponding to Entry 4 of Table 2 in the main text (DMSO-d_6, 600 MHz).

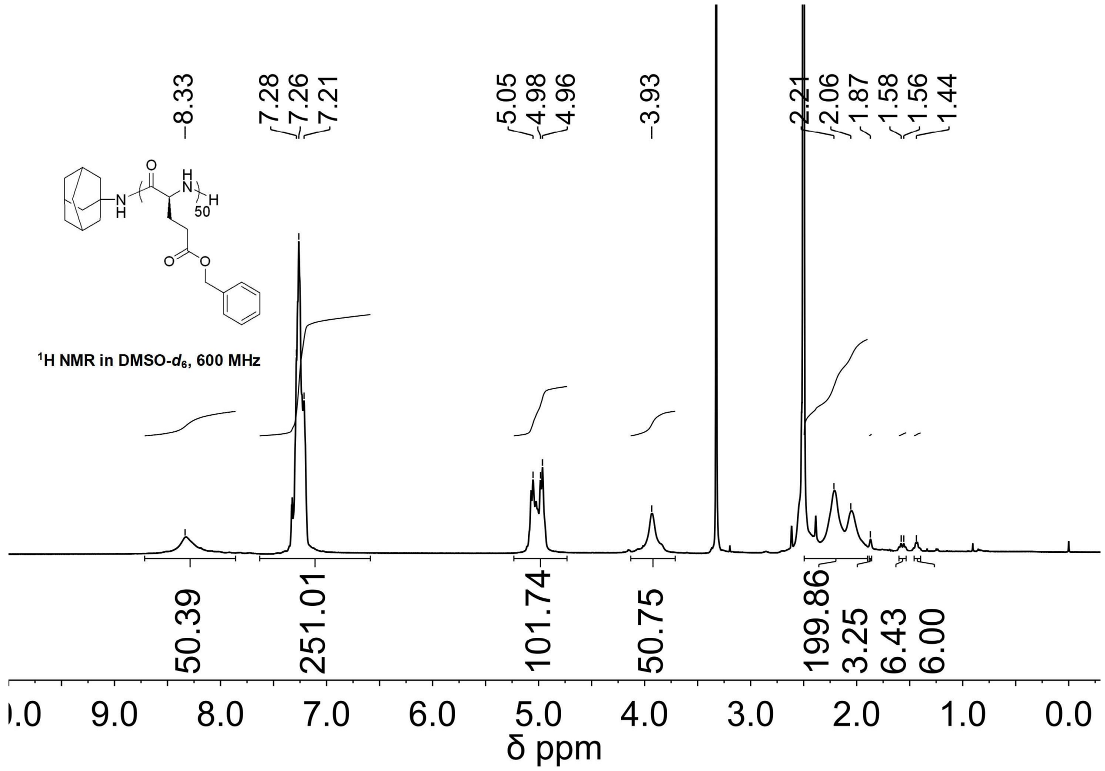

Figure S42. ^{1}H NMR for PBLG, corresponding to Entry 5 of Table 2 in the main text (DMSO-d_6, 600 MHz).

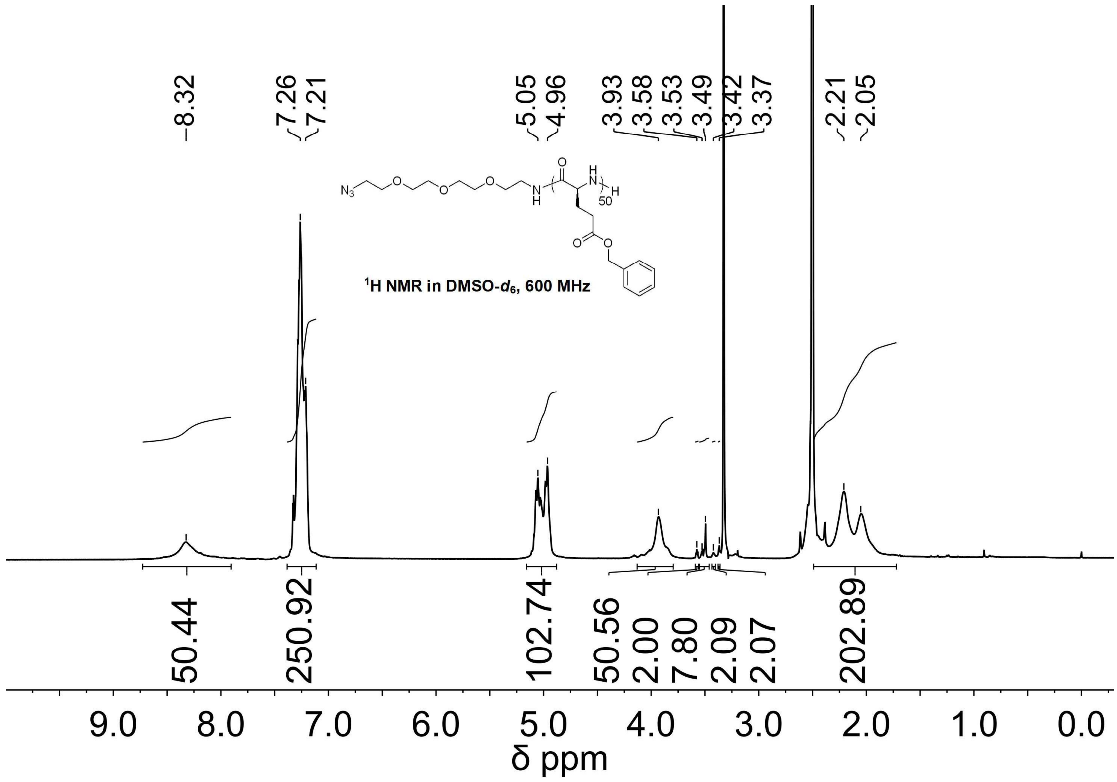

Figure S43. ^{1}H NMR for PBLG, corresponding to Entry 6 of Table 2 in the main text (DMSO-d_6, 600 MHz).

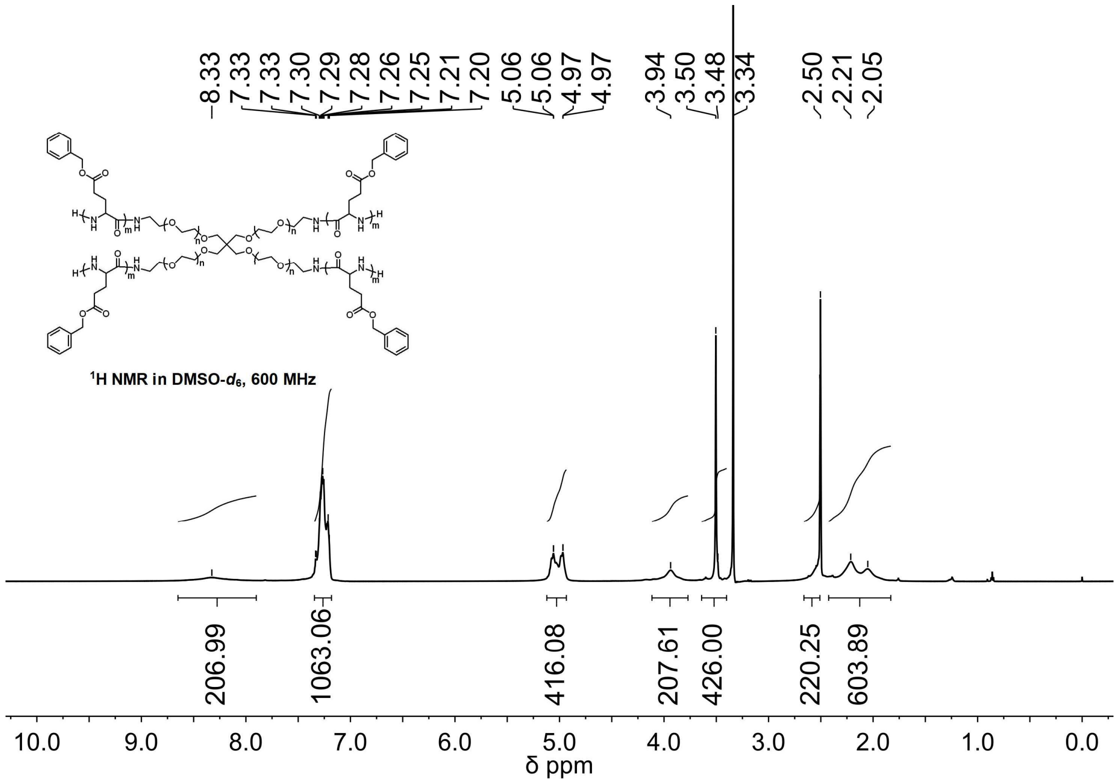

Figure S44. ^{1}H NMR for PBLG, corresponding to Entry 7 of Table 2 in the main text (DMSO-d_6, 600 MHz).

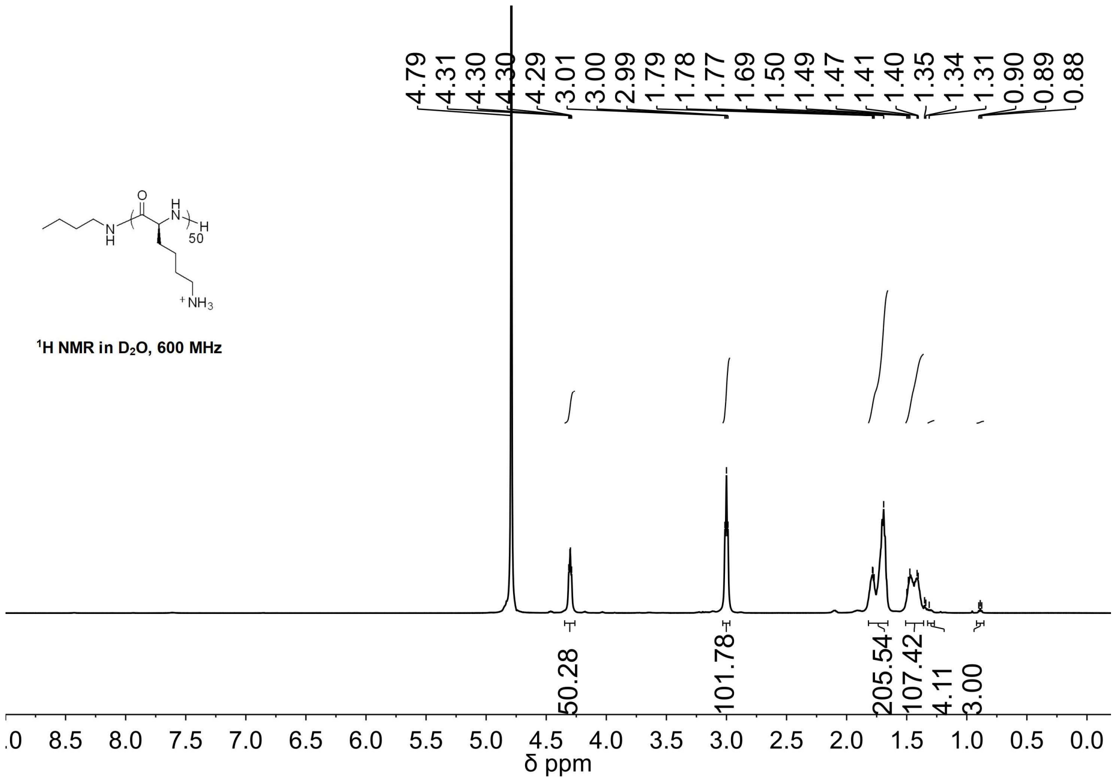

Figure S45. ^{1}H NMR for Poly-L-Lys, corresponding to Entry 8 of Table 2 in the main text (D_2O, 600 MHz).

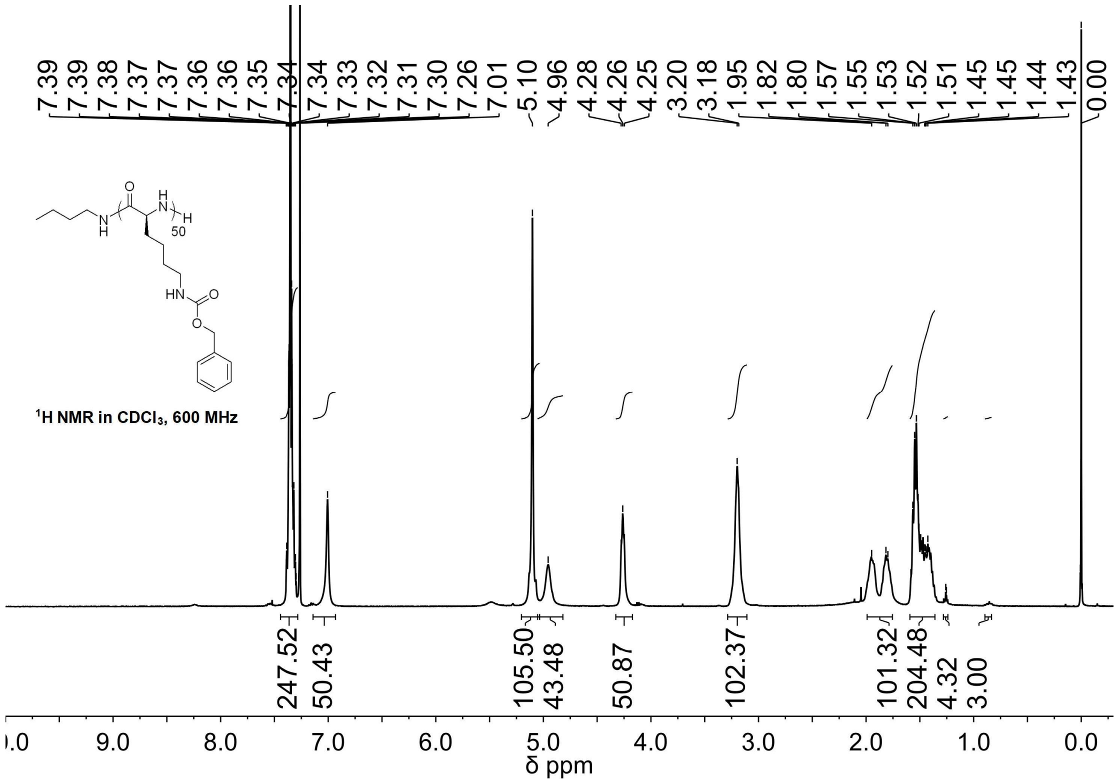

Figure S46. ^{1}H NMR for Poly-cbz-L-Lys, corresponding to Entry 9 of Table 2 in the main text ($CDCl_3$, 600 MHz).

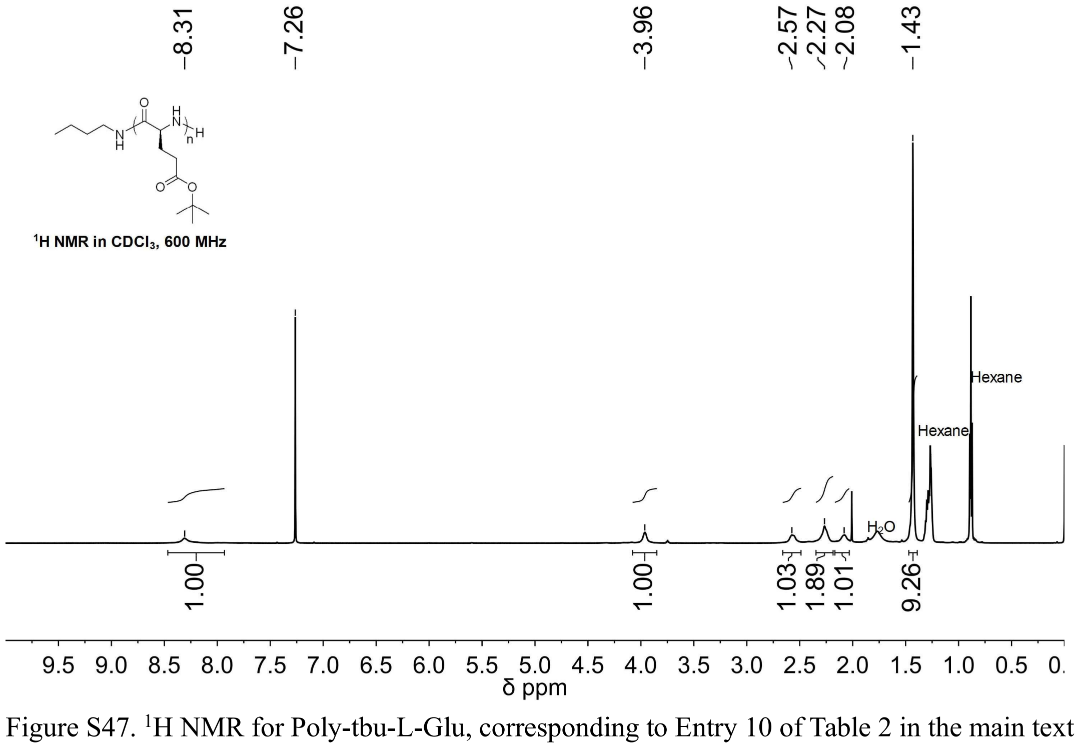

Figure S47. ^{1}H NMR for Poly-tbu-L-Glu, corresponding to Entry 10 of Table 2 in the main text ($CDCl_3$, 600 MHz).

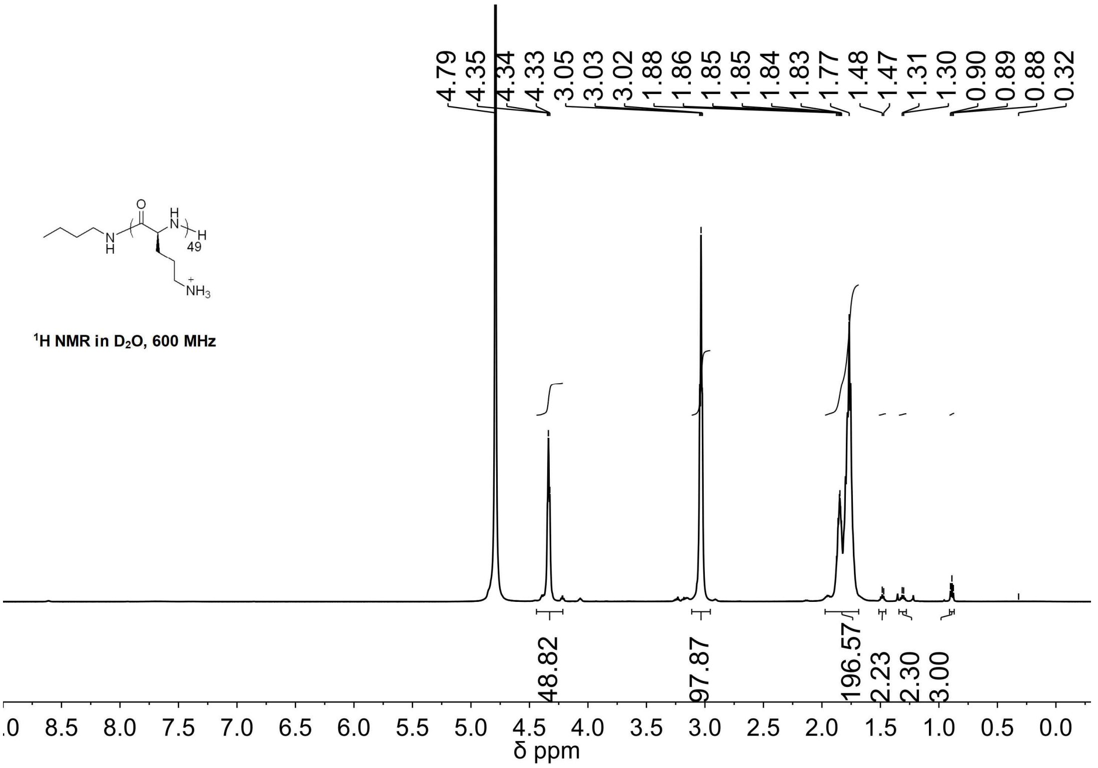

Figure S48. ^{1}H NMR for Poly-L-Orn, corresponding to Entry 11 of Table 2 in the main text (D_2O, 600 MHz).

Table S4 Coordinates of the optimized geometries

Nle NCA

0 1

C	-2.19085100	-1.06309600	-0.07707100
N	-0.94056800	-1.00123300	0.42083400
C	-0.44682500	0.35412000	0.58928500
C	-1.63897800	1.14655700	0.07713900
O	-2.62033100	0.26676300	-0.30280900
O	-2.89685400	-1.99889800	-0.32287200
O	-1.75875400	2.33025800	-0.02215600
C	0.82959200	0.67004600	-0.19982100
C	2.04000100	-0.12553500	0.29285500
C	3.31954700	0.20284400	-0.48265600
C	4.52833000	-0.59871900	0.00419000
H	-0.45017200	-1.83303400	0.71269700
H	-0.30190700	0.59303600	1.64879100
H	1.01816400	1.74363700	-0.10368300
H	0.64585300	0.46308800	-1.25993200
H	1.83604000	-1.19970400	0.20451200
H	2.19714000	0.07743700	1.35973600
H	3.52805000	1.27568800	-0.39411300

H	3.15373000	0.00740500	-1.54882800
H	5.42849800	-0.34799400	-0.56469700
H	4.35600700	-1.67502100	-0.10189700
H	4.73520100	-0.39818300	1.06074600

n-Butylamine
0 1

C	-2.53129500	-0.32147200	-0.00002000
C	-1.25451800	0.52246800	0.00001400
C	0.02351300	-0.32264800	0.00003100
C	1.30879700	0.51496800	-0.00000900
N	2.56554500	-0.24941800	-0.00000800
H	-3.42767100	0.30634500	-0.00005400
H	-2.57642200	-0.96685600	-0.88369300
H	-2.57648000	-0.96683900	0.88366200
H	-1.25102400	1.18088000	0.87746400
H	-1.25098100	1.18089200	-0.87742900
H	0.02936900	-0.98009700	-0.87969700
H	0.02939600	-0.98006400	0.87978600
H	1.31379300	1.17221200	0.87735400
H	1.31377500	1.17217500	-0.87740000
H	2.57921100	-0.86631600	0.80943600
H	2.57924100	-0.86629400	-0.80946900

DMAPPCl
0 1

C	-1.35877800	-1.21311300	-0.27169300
C	-0.02614200	-1.18160400	-0.56715600
N	0.65416900	-0.01598300	-0.72730400
C	-0.02148100	1.15704900	-0.60554900
C	-1.35341900	1.20329100	-0.30744800
C	-2.09420500	-0.00077600	-0.11334500
C	2.10842300	-0.02402600	-1.01172700
H	-1.82809800	-2.17849700	-0.16203400
H	0.54898300	-2.08827500	-0.69004200
H	0.55544900	2.05670900	-0.76547300
H	2.31252900	-0.92045500	-1.59847200
H	2.31484600	0.84338500	-1.63974100
C	3.01652600	0.00365500	0.24086900
H	-1.82059200	2.17335100	-0.23638000
N	-3.40041100	0.00556600	0.18981300
C	-4.13696400	-1.25353200	0.33171200
H	-5.18163800	-1.03015700	0.53038500
H	-4.08032700	-1.84526000	-0.58597400

H	-3.74392100	-1.84702100	1.16234500
C	-4.11598500	1.27112100	0.37292400
H	-5.13372500	1.05813500	0.68945200
H	-3.63728200	1.88085900	1.14369500
H	-4.15303500	1.84182600	-0.55994300
C	2.78208300	1.28231600	1.05969600
H	2.93223800	2.17871100	0.44981300
H	1.77262800	1.31710600	1.47863600
H	3.48455600	1.32503600	1.89658500
C	4.46396800	-0.01441400	-0.28220900
H	5.16674300	0.00736200	0.55490000
H	4.66483300	-0.91792200	-0.86625200
H	4.66912400	0.85397200	-0.91591800
C	2.77325300	-1.23081600	1.12273200
H	1.76254900	-1.23895300	1.54028500
H	2.92082100	-2.15835300	0.56095200
H	3.47333600	-1.23354500	1.96278500

Nle NCA + DMAPPCl (cation-dipole interaction)
1 1

C	1.92361300	-0.02074700	0.67112300
N	2.04309800	0.65966100	-0.49786500
C	2.77943000	0.11389700	-1.49941000
C	3.41712500	-1.08647500	-1.36554200
C	3.32444000	-1.82166800	-0.14747600
C	2.53027700	-1.22722700	0.87628000
H	1.30828500	0.43922500	1.42838000
H	2.82864000	0.68330500	-2.41631300
H	2.38748300	-1.70748800	1.83221100
C	-2.11106100	0.31131500	2.06571600
N	-3.15566500	-0.40385400	1.61377300
C	-3.01410500	-0.84692800	0.23603200
C	-1.64231100	-0.28559800	-0.09554600
O	-1.17429200	0.39657500	1.00003800
O	-1.88759500	0.82152300	3.12508500
O	-1.02230000	-0.36519100	-1.11342800
C	-4.09131500	-0.31491300	-0.71889700
C	-5.48268400	-0.87418300	-0.41414900
C	-6.54965900	-0.35502600	-1.38322300
C	-7.94250700	-0.91178800	-1.08299600
H	-3.94729200	-0.61105600	2.20455200
H	-2.96567200	-1.93941500	0.17644000
H	-3.79048600	-0.58728200	-1.73520200
H	-4.09923100	0.77908400	-0.66064500

H	-5.77361000	-0.61050400	0.61018600
H	-5.44948200	-1.96994200	-0.45892300
H	-6.26127700	-0.61598800	-2.40847300
H	-6.57324300	0.74034200	-1.33827500
H	-8.68535400	-0.52693400	-1.78770800
H	-8.26766500	-0.63811600	-0.07367300
H	-7.95341900	-2.00467900	-1.15068400
C	3.75805200	-3.76081700	1.26285200
H	2.70100100	-3.98033700	1.43822700
H	4.29346000	-4.70307800	1.18085900
H	4.15319300	-3.21003400	2.12156000
C	4.76858700	-3.57179200	-1.04177300
H	4.16675500	-3.83691300	-1.91665800
H	5.54743800	-2.86803800	-1.34663400
H	5.25141200	-4.47106300	-0.66794000
N	3.93750100	-3.00336100	0.02201100
H	3.97440700	-1.45975800	-2.21077400
C	1.37751600	1.96937600	-0.68075600
H	0.43934500	1.92838500	-0.13022700
H	1.13990500	2.05607600	-1.74140200
C	2.20911800	3.18993900	-0.22129400
C	1.34548300	4.42795500	-0.52278600
H	0.39529600	4.39001300	0.01915800
H	1.86998900	5.33746000	-0.21781900
H	1.12559200	4.50824600	-1.59198800
C	2.49553200	3.12201500	1.28722200
H	1.56955700	3.03438800	1.86393400
H	3.14005800	2.27583000	1.54129700
H	3.00892800	4.03162800	1.61109900
C	3.53027100	3.27899400	-1.00008100
H	4.18105800	2.42524600	-0.79057600
H	3.35450000	3.31940600	-2.07967200
H	4.07457900	4.18363900	-0.71508700

n-Butylamine + DMAPPCl

1 1

C	0.11728400	1.59283800	-1.21093700
C	-0.81457800	0.59568500	-1.26357500
N	-1.27778000	-0.02302200	-0.14694500
C	-0.79033100	0.35712100	1.06221400
C	0.13522700	1.35312300	1.19269800
C	0.64224900	2.02501200	0.04245000
C	-2.28640500	-1.10231000	-0.24217600
H	0.44148700	2.03090800	-2.14199900

H	-1.22119000	0.24821700	-2.20268800
H	-1.16254100	-0.18773800	1.91608400
H	-2.12539200	-1.60173800	-1.19873100
H	-2.04930600	-1.81419600	0.54785400
C	5.28808700	-2.56268900	-0.76087700
C	4.05925000	-1.76032800	-0.32751500
C	2.88202700	-2.64513200	0.09590400
C	1.65526800	-1.83681500	0.53171200
N	0.48684600	-2.62303600	0.95440700
H	6.11114900	-1.90648800	-1.05976500
H	5.64890200	-3.19982200	0.05327800
H	5.05410000	-3.21236200	-1.61076800
H	3.74064600	-1.10618900	-1.14891700
H	4.32919500	-1.09871200	0.50495800
H	3.19116800	-3.29891300	0.92186100
H	2.59940400	-3.30453700	-0.73522100
H	1.34219500	-1.18334000	-0.28983900
H	1.93151200	-1.17511300	1.35965900
H	0.20121600	-3.23805600	0.19604000
H	0.75200200	-3.23686200	1.72131100
C	-3.75546800	-0.63009300	-0.13768300
H	0.48161900	1.59235800	2.18625000
N	1.56773000	2.99301800	0.13143200
C	2.08003200	3.64524900	-1.07643400
H	2.81290400	4.39415900	-0.78866900
H	2.56597600	2.92154300	-1.73743200
H	1.27518800	4.14284300	-1.62492400
C	2.09139000	3.39919800	1.43759400
H	2.81687700	4.19585800	1.29569900
H	1.29026100	3.77252200	2.08167800
H	2.58838700	2.56341500	1.93886300
C	-4.02465200	0.03503300	1.22103400
H	-3.77103700	-0.63352200	2.04937700
H	-3.45334500	0.96005100	1.33830400
H	-5.08416500	0.29126300	1.30788100
C	-4.62004000	-1.89669000	-0.27015100
H	-5.68038900	-1.63821800	-0.20532900
H	-4.45361000	-2.39417100	-1.23087100
H	-4.39824100	-2.61301000	0.52709500
C	-4.09762000	0.34836100	-1.27189600
H	-3.52023600	1.27431000	-1.19672900
H	-3.90627600	-0.09700100	-2.25323100
H	-5.15636300	0.61844500	-1.22751500

Nle NCA + DMAPPCl (C-H--O hydrogen bonding interaction)

1 1

C	-1.81598300	-0.16505800	-0.18967500
N	-2.61374500	0.74547200	0.42844900
C	-3.89056100	0.39740100	0.73594800
C	-4.40204400	-0.83332000	0.43822600
C	-3.59535400	-1.81218400	-0.21482500
C	-2.25938700	-1.41505000	-0.51539400
H	-0.80571500	0.15264500	-0.40542800
H	-4.48081500	1.15220900	1.23615000
H	-1.56320600	-2.08556400	-0.99529000
C	2.27375100	-0.60834900	2.11975500
N	3.49276600	-0.22390900	1.70245200
C	3.49144900	0.42941800	0.40381200
C	2.01136900	0.37043900	0.07150900
O	1.34985800	-0.23820500	1.10023900
O	1.90657400	-1.15581500	3.11762000
O	1.45015500	0.75380900	-0.91460500
C	4.34835000	-0.26393400	-0.66324900
C	5.84543300	-0.19825800	-0.35356900
C	6.69797000	-0.87922400	-1.42876000
C	8.19600600	-0.81492900	-1.12553000
H	4.31193000	-0.36054900	2.27608800
H	3.78281700	1.48202500	0.49228100
H	4.14013600	0.22680100	-1.61875700
H	4.02206800	-1.30608700	-0.75086400
H	6.04584600	-0.67266700	0.61508400
H	6.14792300	0.85196900	-0.25696800
H	6.49750900	-0.40713600	-2.39793500
H	6.38650100	-1.92622400	-1.52454600
H	8.78162700	-1.30709500	-1.90746800
H	8.42789900	-1.30791500	-0.17559000
H	8.54013100	0.22217900	-1.05441500
C	-2.10291400	2.09801700	0.75189700
H	-2.61540300	2.42163500	1.65919400
H	-1.04385800	1.99020200	0.98652700
C	-2.29541300	3.14933200	-0.36731400
C	-1.65756100	4.44871900	0.15597000
H	-1.76784100	5.24665800	-0.58319500
H	-2.13392800	4.78125500	1.08360700
H	-0.58866500	4.31500200	0.34988300
C	-1.58764000	2.71476200	-1.66036300
H	-0.52954400	2.50053000	-1.48661200
H	-2.04784500	1.82203700	-2.09351200

H	-1.65578100	3.51152400	-2.40636800
C	-3.78866400	3.38390200	-0.64411400
H	-4.27500400	2.48667000	-1.03712900
H	-4.31873200	3.69355000	0.26201200
H	-3.91048100	4.17426600	-1.39008200
C	-5.43576900	-3.40924400	-0.17513300
H	-5.59629600	-3.36708900	0.90617400
H	-6.15684700	-2.75011100	-0.66684800
H	-5.61666700	-4.42704600	-0.51027500
C	-3.19691100	-4.01664800	-1.17373500
H	-2.33434900	-4.26081600	-0.54666400
H	-3.76571500	-4.92640300	-1.34678300
H	-2.84069700	-3.64389300	-2.13814900
N	-4.06226000	-3.03276400	-0.51833300
H	-5.42378300	-1.03721600	0.71914600

TS1
1 1

C	2.19290500	-2.14277600	-0.39503800
H	3.05556100	-2.67326900	-0.80339500
C	0.92400000	-2.56678400	-1.13025300
H	0.05666800	-2.03266600	-0.73536100
H	1.01376300	-2.33988400	-2.19493600
C	2.40572900	-0.61248000	-0.46818200
N	2.11054300	-2.33677600	1.05053500
H	1.81387900	-3.22012900	1.44531000
C	1.71470000	-1.19255300	1.68468100
O	1.86942300	-0.14000300	0.82539300
O	1.94612500	0.06338200	-1.52070700
O	1.29893800	-1.05828500	2.81790700
H	0.76244900	-3.64240700	-1.00837600
N	3.84769100	-0.08525500	-0.51030100
H	3.15878100	0.53133700	-1.38146400
H	4.09604100	0.38937900	0.35933400
C	4.94066800	-0.95500800	-0.97721500
H	5.14335000	-1.76063800	-0.26589900
H	4.67371100	-1.37580300	-1.94867400
H	5.83715900	-0.34304400	-1.09376200
C	-1.91611400	-0.66105700	1.04936500
C	-1.06154100	0.40864400	1.02143300
N	-0.79105400	1.08762000	-0.12329500
C	-1.35788300	0.67033300	-1.28474500
C	-2.23223900	-0.38257400	-1.33199100
C	-2.56524800	-1.09820900	-0.14318900

C	0.11006900	2.25991000	-0.10619300
H	-2.07079600	-1.15748400	1.99656600
H	-0.54292900	0.74087900	1.91000300
H	-1.07541100	1.21645700	-2.17471100
H	0.82669400	2.08782800	0.69702000
H	0.66703300	2.23795300	-1.04380400
C	-0.60859700	3.61478200	0.09136300
H	-2.63803400	-0.66130600	-2.29402800
N	-3.42100600	-2.13769500	-0.14943000
C	-3.68420600	-2.88389400	1.08219300
H	-4.35629900	-3.71039200	0.85839000
H	-2.75824400	-3.29656700	1.49711900
H	-4.15609000	-2.24621700	1.83782000
C	-4.08073500	-2.54774300	-1.39018900
H	-4.78198700	-3.35054600	-1.16918300
H	-4.64111400	-1.71614500	-1.82972700
H	-3.35381300	-2.91286000	-2.12462900
C	-1.58811000	3.89717300	-1.05950800
H	-1.08226700	3.86525900	-2.03121400
H	-2.41390400	3.17795200	-1.07938900
H	-2.02720500	4.89372100	-0.94358500
C	0.49756000	4.68603800	0.09789100
H	0.06214400	5.68111300	0.23538000
H	1.21169900	4.51659300	0.91187700
H	1.05390700	4.69062300	-0.84644500
C	-1.35959000	3.64286000	1.43282200
H	-2.16958200	2.90601800	1.46064300
H	-0.68474700	3.43935300	2.27205800
H	-1.80772600	4.62891000	1.59428100

INT2

1 1

N	3.95045700	0.05148900	0.58700900
C	4.36205800	1.25547400	0.12024600
C	5.84738000	1.57197900	0.42629100
N	6.72480500	0.75675600	-0.42373600
C	2.62639700	-0.46990400	0.29838800
O	3.63351500	2.03408600	-0.49477000
C	6.15617300	3.04879400	0.22623300
C	7.04417600	-0.52541200	-0.09991700
O	6.64421500	-1.10468700	0.90297300
H	4.65602500	-0.57067700	0.97035500
H	6.05770000	1.27638300	1.45823100
H	6.91739000	1.11195100	-1.35212600

H	2.56208500	-0.90082700	-0.70928000
H	2.38048300	-1.24447900	1.02731100
H	1.89465400	0.33752000	0.36952100
H	7.21050700	3.24619800	0.43846100
H	5.54172000	3.65489800	0.89630300
H	5.92926400	3.35641400	-0.79866300
O	7.87277600	-1.17052000	-0.95601700
H	8.21181900	-0.57439300	-1.64386900
C	-4.37668100	-0.96948300	1.08570800
C	-5.33686700	0.00290100	1.15551400
C	-5.81587000	0.63300800	-0.03301700
C	-5.24411000	0.17166800	-1.25729000
C	-4.28433200	-0.80359400	-1.25016100
H	-4.00092000	-1.46501000	1.97187300
H	-5.71996300	0.26644500	2.13110000
H	-5.55176500	0.57195200	-2.21265100
H	-3.83445500	-1.16733000	-2.16501400
N	-6.75369500	1.59534100	-0.00171800
C	-7.23414400	2.20163000	-1.24584500
H	-7.97678300	2.96024500	-1.00645000
H	-6.41429900	2.68105300	-1.79099800
H	-7.70061000	1.45157700	-1.89363500
C	-7.31662400	2.03788600	1.27602500
H	-6.53406800	2.43329200	1.93223800
H	-8.04080700	2.82842100	1.08944900
H	-7.82659800	1.21481300	1.78823100
N	-3.83896300	-1.37368000	-0.09744400
C	-2.76411900	-2.39173400	-0.12715800
H	-2.95532100	-3.08075700	0.69853900
H	-2.87736100	-2.94617200	-1.06171700
C	-1.32948500	-1.81844100	-0.01932800
C	-0.38012800	-3.02991200	-0.07218000
H	-0.49523700	-3.58660600	-1.00897700
H	0.65984400	-2.69637800	-0.00649600
H	-0.56585000	-3.71964900	0.75893600
C	-1.13759900	-1.07024800	1.31013500
H	-1.77193600	-0.17992400	1.37157400
H	-1.36487100	-1.71383800	2.16765500
H	-0.09971900	-0.73767300	1.40697400
C	-1.02602600	-0.87597100	-1.19540200
H	-1.16597900	-1.37983700	-2.15853200
H	-1.66146700	0.01575200	-1.17944900
H	0.01306400	-0.53558700	-1.14462600

TS2

1 1

N	-3.78967000	-0.94965100	1.18922800
C	-4.70137400	-0.25370200	0.47127100
C	-4.13903500	0.69429400	-0.61673500
N	-2.91841100	0.16274100	-1.29257400
C	-4.20743600	-1.80767400	2.28731200
O	-5.91441900	-0.34306200	0.65371300
C	-5.19022000	0.99648300	-1.67862900
C	-1.56324100	0.42128000	-0.68253700
O	-1.18539000	0.10987800	0.42866000
H	-2.80391000	-0.71248200	1.11233000
H	-3.83875600	1.62485300	-0.12055100
H	-3.03204100	-0.82626200	-1.53181400
H	-4.56192700	-1.22606500	3.14705300
H	-5.02119600	-2.46075600	1.96285000
H	-3.35841000	-2.41935000	2.59819400
H	-4.81059800	1.74133100	-2.38281000
H	-6.09489300	1.37657900	-1.20248400
H	-5.45489100	0.08931500	-2.23115000
O	-1.00243800	1.05578200	-1.66304100
H	-2.15063100	0.85490300	-2.13211700
C	1.89278100	0.09014100	-1.04533000
C	2.23771600	-1.21683800	-1.26300800
C	2.52195400	-2.08258400	-0.16526400
C	2.41540000	-1.49860000	1.13249200
C	2.06580200	-0.18269300	1.27267000
H	1.64795500	0.75829900	-1.85861400
H	2.27549400	-1.56646400	-2.28496600
H	2.60117900	-2.07146900	2.03002300
H	1.97163000	0.28121700	2.24592200
N	2.85971100	-3.37356400	-0.34115200
C	3.12673100	-4.23125300	0.81488400
H	3.33471400	-5.24122100	0.46663900
H	3.99450600	-3.87332100	1.38026400
H	2.26040000	-4.26945900	1.48390400
C	2.98007100	-3.93054800	-1.69005400
H	3.73264900	-3.38904400	-2.27330600
H	3.28712500	-4.97248400	-1.61767400
H	2.02290100	-3.88807700	-2.22136900
N	1.81485100	0.61804800	0.20432200
C	1.44464700	2.03721400	0.39891600
H	0.74398100	2.28980500	-0.39845600
H	0.90640800	2.09504800	1.34693100

C	2.63998700	3.01932400	0.40147600
C	2.04013200	4.42049300	0.62027900
H	1.50550700	4.48105900	1.57509900
H	2.83337200	5.17491100	0.63132200
H	1.33830900	4.68266700	-0.17960600
C	3.38261500	2.98261000	-0.94457900
H	3.85164600	2.00931100	-1.12433100
H	2.70543400	3.19551600	-1.77968800
H	4.17709600	3.73601900	-0.95664900
C	3.61606400	2.69158400	1.54328100
H	3.11141400	2.70345000	2.51617800
H	4.08083500	1.70871800	1.41113500
H	4.42176700	3.43247300	1.57565500

INT3

1 1

N	2.69558300	0.29500400	0.86756200
C	3.59926600	-0.44958100	0.19492000
C	3.49538500	-1.97368400	0.42719600
N	2.53721800	-2.52505100	-0.57845500
C	2.63932900	1.73811100	0.70313300
O	4.45257300	0.02521300	-0.55409600
C	4.85768300	-2.64469600	0.31569300
C	0.87025800	-2.18329300	-0.31414300
O	0.67221800	-1.88291500	0.85113200
H	1.92213000	-0.18439500	1.31311600
H	3.03782900	-2.17626500	1.39754200
H	2.76367300	-2.21430600	-1.52682400
H	3.62181700	2.17650700	0.89696400
H	2.33601000	2.01730000	-0.31157700
H	1.91946500	2.14204300	1.41508900
H	4.76158600	-3.72911900	0.42808400
H	5.51624800	-2.27508500	1.10654800
H	5.32196000	-2.41720800	-0.64662400
O	0.27503400	-2.34466100	-1.36192400
H	2.59859600	-3.54711200	-0.59223300
C	-1.26770700	0.30463400	-1.03304000
C	-0.68128200	1.52786600	-1.22110100
C	-0.57235400	2.45254400	-0.14069900
C	-1.07296400	2.00162500	1.11774000
C	-1.64517200	0.76277300	1.23099600
H	-1.34338700	-0.42208500	-1.82905100
H	-0.30447800	1.76108500	-2.20676700
H	-1.01432000	2.61445000	2.00616800

H	-2.03160000	0.39792300	2.17359600
N	-0.00927700	3.66515700	-0.29391400
C	0.10052100	4.58151200	0.84215400
H	0.55676900	5.51053300	0.50492600
H	-0.88609300	4.81246900	1.25745100
H	0.72705000	4.15710400	1.63457900
C	0.51447800	4.07497600	-1.59786800
H	-0.27984700	4.09391100	-2.35199700
H	0.93271200	5.07606700	-1.51261500
H	1.30661600	3.39780100	-1.93611500
N	-1.76422600	-0.07998400	0.17210900
C	-2.42657100	-1.39505900	0.32557900
H	-1.93012500	-2.07512000	-0.36749600
H	-2.21287300	-1.73944000	1.33873600
C	-3.95236000	-1.37357200	0.07153700
C	-4.43806400	-2.82002000	0.27978400
H	-4.23598400	-3.16490000	1.30038500
H	-5.51780500	-2.88582400	0.11077400
H	-3.94620400	-3.50996500	-0.41516600
C	-4.25972000	-0.92879200	-1.36795600
H	-3.94971100	0.10580300	-1.54842200
H	-3.75527000	-1.56953500	-2.10009400
H	-5.33666900	-0.98555000	-1.55813000
C	-4.66468000	-0.44379300	1.06744700
H	-4.45067700	-0.72857600	2.10395000
H	-4.36849200	0.60189700	0.93189800
H	-5.74888100	-0.49669100	0.92307300

TS4

1 1

C	-2.19007100	2.14526200	-0.39363500
H	-3.05146900	2.67752100	-0.80237200
C	-0.91969800	2.56800200	-1.12702200
H	-0.05365500	2.03223200	-0.73157500
H	-1.00858100	2.34210100	-2.19198800
C	-2.40522900	0.61537600	-0.46868700
N	-2.10896200	2.33755400	1.05225900
H	-1.81109800	3.21994800	1.44829600
C	-1.71560200	1.19196000	1.68551900
O	-1.87100400	0.14064200	0.82487800
O	-1.94559200	-0.06005500	-1.52148800
O	-1.30134500	1.05568800	2.81905100
H	-0.75664100	3.64327600	-1.00406500
N	-3.84796100	0.09038600	-0.51283400

H	-3.15910200	-0.52629500	-1.38397900
H	-4.09791900	-0.38491100	0.35599200
C	-4.93914200	0.96231800	-0.97988400
H	-5.14102900	1.76773000	-0.26809600
C	1.91773300	0.65906300	1.04945800
C	1.06135400	-0.40920000	1.02144500
N	0.78957900	-1.08751300	-0.12338300
C	1.35689700	-0.67091000	-1.28483800
C	2.23293700	0.38058600	-1.33203200
C	2.56726500	1.09547700	-0.14316300
C	-0.11294600	-2.25873300	-0.10641400
H	2.07362600	1.15492900	1.99676200
H	0.54239200	-0.74090000	1.91002300
H	1.07344200	-1.21647900	-2.17483400
H	-0.82949200	-2.08584300	0.69668100
H	-0.66972500	-2.23602200	-1.04412000
C	0.60399000	-3.61452800	0.09110000
H	2.63893500	0.65890200	-2.29409700
N	3.42461500	2.13363600	-0.14942900
C	3.69002500	2.87868900	1.08240100
H	4.36156400	3.70551100	0.85810700
H	2.76478700	3.29088100	1.49934600
H	4.16332100	2.24039100	1.83664600
C	4.08593400	2.54177200	-1.38998000
H	4.78776700	3.34409300	-1.16909700
H	4.64583100	1.70914900	-1.82823500
H	3.36004400	2.90693600	-2.12540100
C	1.58385200	-3.89772500	-1.05927400
H	1.07865600	-3.86476100	-2.03127900
H	2.41061000	-3.17958700	-1.07836000
H	2.02156100	-4.89489800	-0.94348300
C	-0.50350300	-4.68441700	0.09661300
H	-0.06942400	-5.68007000	0.23414900
H	-1.21798000	-4.51427100	0.91015700
H	-1.05922800	-4.68807500	-0.84809100
C	1.35414900	-3.64406600	1.43300200
H	2.16517100	-2.90838700	1.46153600
H	0.67909400	-3.43986000	2.27189700
H	1.80080200	-4.63079500	1.59442600
C	-4.56056892	1.55627533	-2.34937190
H	-5.44388550	1.68242651	-2.93991208
H	-4.08748844	2.50553810	-2.20796800
H	-3.88729837	0.89354582	-2.85176254
C	-8.70500988	0.35803258	-1.23985101

H	-9.50686025	0.90049020	-1.69556552
H	-8.73950998	-0.66320140	-1.55732684
H	-8.80289517	0.40410629	-0.17533437
C	-6.20510196	0.10129640	-1.14597791
O	-6.28698362	-1.13039985	-0.90145733
N	-7.36040832	0.98080115	-1.65914319
H	-8.10075727	1.47394741	-2.11596941

INT5

1 1

N	-5.75410200	-0.52942400	0.12421400
C	-7.04993400	-0.80188700	0.40393100
C	-7.78921600	0.33361300	1.14867400
N	-8.16941000	1.37591400	0.18718400
C	-4.93959600	-1.41806400	-0.68519200
O	-7.61274600	-1.84806500	0.07550600
C	-9.02259700	-0.18602900	1.87367400
C	-7.27655100	2.33116700	-0.20358900
O	-6.15307000	2.45800900	0.25988400
H	-5.37379600	0.39213700	0.32298000
H	-7.09993500	0.80294400	1.85522400
H	-8.97429100	1.17447600	-0.39380500
H	-5.16947500	-2.44718700	-0.39053200
H	-9.54784600	0.63841400	2.36345000
H	-8.73178600	-0.91857300	2.63115600
H	-9.70048900	-0.68358100	1.17456500
O	-7.71361600	3.19702200	-1.15242000
H	-8.65263700	3.05759600	-1.35726600
C	-5.23228700	-1.25007500	-2.18689200
H	-4.62518800	-1.93688200	-2.78474500
H	-6.28821200	-1.46108400	-2.37452700
H	-5.01397700	-0.22434800	-2.50111000
C	-1.16678700	-1.96419200	-0.50187100
H	-0.88113300	-0.92891200	-0.69284000
H	-0.85911200	-2.22255600	0.51861000
H	-0.64612900	-2.61897100	-1.20436100
N	-2.60171200	-2.10169300	-0.68540800
H	-2.97286300	-3.00965800	-0.92857800
C	-3.46604300	-1.10218900	-0.39376500
O	-3.11125600	0.00420600	0.01672200
C	7.43760500	1.38959900	-0.53050300
C	6.10447000	1.12233700	-0.68491800
N	5.58718800	-0.12178300	-0.49541600
C	6.42697900	-1.13629500	-0.15287600

C	7.77056300	-0.94399400	0.01952300
C	8.34550400	0.35133500	-0.15915500
C	4.13172900	-0.36180900	-0.62993900
H	7.77273600	2.40229700	-0.70515100
H	5.39826200	1.89120600	-0.96988400
H	5.96989000	-2.10950500	-0.02827600
H	3.76464800	0.33309000	-1.38870300
H	4.01194200	-1.37728900	-1.01532600
C	3.32374500	-0.18930800	0.68000400
H	8.37311400	-1.80119000	0.28531600
N	9.66083300	0.57676400	0.00230000
C	10.21509900	1.91572200	-0.21216000
H	11.28653900	1.89003900	-0.02265500
H	10.05364800	2.24794500	-1.24337800
H	9.76077900	2.64167600	0.47021300
C	10.56108100	-0.51883200	0.36946500
H	11.57125500	-0.12716500	0.47206500
H	10.26586200	-0.96540900	1.32474200
H	10.56703000	-1.29847100	-0.40019400
C	3.79939400	-1.17980300	1.75560600
H	3.74199300	-2.21484800	1.39991900
H	4.83035500	-0.98072100	2.06696700
H	3.16790200	-1.09650000	2.64606100
C	1.85749400	-0.48953100	0.31648800
H	1.21936700	-0.38023200	1.19864100
H	1.48808800	0.19929500	-0.45120800
H	1.74244800	-1.51240500	-0.05900100
C	3.44039200	1.24957700	1.20760000
H	4.46812800	1.49462300	1.49581700
H	3.11059500	1.97818100	0.45838700
H	2.81196700	1.37570300	2.09515800

TS5
1 1

N	3.38602000	-0.16202700	-0.27685100
C	3.82206500	-1.20739800	-1.01464200
C	2.74092300	-2.23351600	-1.43240200
N	1.60626600	-1.57257700	-2.14535200
C	4.30249700	0.88117000	0.15571900
O	4.99910000	-1.36162800	-1.34292300
C	3.32117200	-3.32196800	-2.32428400
C	0.42296800	-1.11210900	-1.33024500
O	0.44861200	-0.39403300	-0.35411500
H	2.40674900	-0.06156100	-0.01014800

H	2.31815200	-2.67651400	-0.52385600
H	1.93814600	-0.82866700	-2.76536400
H	5.25725300	0.40906100	0.40836400
H	2.54884800	-4.05766300	-2.56390200
H	4.14651600	-3.82236400	-1.81446300
H	3.70949100	-2.89519500	-3.25322500
O	-0.52435000	-1.74209200	-1.95037100
H	0.49486000	-2.15238800	-2.57410700
C	-3.21406600	-0.54674800	-0.78245400
C	-3.68548600	0.58983800	-1.38270600
C	-3.41928300	1.87099600	-0.81393800
C	-2.63648100	1.87260000	0.37929900
C	-2.19826400	0.69483300	0.92128000
H	-3.39104000	-1.52732900	-1.20194800
H	-4.25057500	0.48037200	-2.29744700
H	-2.35774700	2.79129300	0.87615000
H	-1.59019700	0.67098400	1.81574400
N	-3.87014100	3.00846900	-1.37585300
C	-3.56890200	4.30531500	-0.76712300
H	-4.04610800	5.09108600	-1.35023000
H	-3.95217100	4.35726200	0.25743800
H	-2.48953300	4.49299500	-0.75063200
C	-4.65733800	2.96103600	-2.60929700
H	-5.58439700	2.39632900	-2.46243400
H	-4.91638200	3.97620800	-2.90419900
H	-4.08761500	2.50032600	-3.42380800
N	-2.49193400	-0.51041400	0.36724200
C	-2.01285100	-1.76123200	0.99446000
H	-1.80443700	-2.45907800	0.18131300
H	-1.06345200	-1.52373100	1.47650000
C	-2.99443000	-2.39029600	2.01082600
C	-2.30064900	-3.65692500	2.54540000
H	-1.35461300	-3.41420500	3.04263000
H	-2.94259100	-4.16182700	3.27449500
H	-2.08747400	-4.36667300	1.73791000
C	-4.31673900	-2.77720200	1.32853300
H	-4.85567800	-1.89874800	0.95824900
H	-4.14599400	-3.45491100	0.48416500
H	-4.97361300	-3.28740400	2.04072800
C	-3.27065400	-1.42436900	3.17445600
H	-2.34265000	-1.12762700	3.67627800
H	-3.78167400	-0.51668800	2.83620800
H	-3.91488700	-1.90440800	3.91859000
C	4.54134700	1.91791900	-0.95626200

H	5.25801900	2.68029000	-0.63635600
H	4.94168300	1.41401400	-1.83970600
H	3.60141600	2.41166500	-1.22338400
C	4.25728900	3.03518400	3.30684900
H	4.49875100	4.09355300	3.16056800
H	3.18443100	2.93448500	3.47305200
H	4.79068000	2.67222600	4.19172700
C	3.71313700	1.55552100	1.40287200
O	2.51236100	1.48206200	1.67004700
N	4.61168200	2.25549400	2.13350300
H	5.58127100	2.24185100	1.84712800

INT6

1 1

N	3.06017800	0.09601200	0.20332000
C	3.85253400	-0.89197100	-0.26371400
C	3.72354800	-2.24164300	0.47580500
N	2.72770200	-3.04849100	-0.29982800
C	2.92966400	1.37048900	-0.48785400
O	4.61543100	-0.77296800	-1.22294000
C	5.06306100	-2.95959900	0.56047600
C	1.12830900	-2.48105100	-0.24013800
O	0.92310700	-1.90896700	0.82367400
H	2.33436200	-0.12188500	0.87896300
H	3.28316100	-2.10615300	1.46469800
H	2.99851300	-3.10900200	-1.28528200
H	3.92940900	1.69605800	-0.78917200
H	4.94164100	-3.95172700	1.00595500
H	5.74856900	-2.38364300	1.18869200
H	5.50961600	-3.05758300	-0.43194400
O	0.54781000	-2.78473100	-1.26349100
H	2.69770800	-4.00696400	0.05884800
C	-3.75136900	0.18285600	-0.95186700
C	-3.68723600	1.54213100	-0.79661200
C	-2.52091800	2.14990400	-0.24193600
C	-1.47115400	1.26177700	0.13760700
C	-1.60493300	-0.08778600	-0.04801700
H	-4.61971000	-0.30294400	-1.37867500
H	-4.53409200	2.12996600	-1.12205000
H	-0.54729000	1.61478800	0.57786600
H	-0.81739200	-0.77584700	0.23667600
N	-2.40940800	3.48274600	-0.09146400
C	-1.15713800	4.06152700	0.40146500
H	-1.25071300	5.14599300	0.41561500

H	-0.93497900	3.71671100	1.41568000
H	-0.31835600	3.79011000	-0.24757600
C	-3.50814300	4.36967400	-0.47456700
H	-4.42863900	4.10411700	0.05532400
H	-3.24577600	5.39234600	-0.20895000
H	-3.69605200	4.32758100	-1.55362200
N	-2.72892600	-0.63556600	-0.58734000
C	-2.82950200	-2.10394900	-0.75297800
H	-3.48152600	-2.28891000	-1.61080600
H	-1.82732300	-2.45857400	-1.00392100
C	-3.36977200	-2.85753500	0.48759500
C	-3.33168900	-4.35315900	0.12308200
H	-2.30763900	-4.68387800	-0.08469000
H	-3.71874800	-4.95501100	0.95175600
H	-3.94365200	-4.56685200	-0.76085800
C	-4.81508600	-2.43328500	0.79303500
H	-4.87825200	-1.37188800	1.05569200
H	-5.47506300	-2.61483700	-0.06314700
H	-5.20530000	-3.00399700	1.64241800
C	-2.48337900	-2.60872400	1.71982900
H	-1.43224600	-2.83828600	1.51682600
H	-2.53946400	-1.56800600	2.05671400
H	-2.81732600	-3.23813200	2.55161300
C	2.03419600	1.24976700	-1.73392900
H	1.96046200	2.21036400	-2.25330900
H	2.46156900	0.51364600	-2.41910700
H	1.02857700	0.92661000	-1.44849600
C	2.12883400	4.76292200	1.08866300
H	1.52861900	5.44099700	0.47350400
H	1.50616200	4.35564300	1.88500400
H	2.95361600	5.32823500	1.53307700
C	2.31208000	2.36876800	0.50026900
O	1.52801900	1.98619000	1.37765800
N	2.64328700	3.65914100	0.29343300
H	3.32133400	3.87120000	-0.42666600

INT1’
0 1

C	1.11343800	-0.21775300	-0.76604900
H	0.62857100	-0.99373400	-1.36606900
C	2.59169400	-0.07974300	-1.12907000
H	3.07552400	0.67772000	-0.50532600
H	3.09761700	-1.03791500	-0.98243800
C	0.91241700	-0.58553000	0.69746600

N	0.36744900	1.02699200	-0.82469600
H	0.10310100	1.49034500	-1.68231200
C	-0.24926700	1.33279000	0.33804600
O	0.16049200	0.39573500	1.30120200
O	1.35619600	-1.52236200	1.30318100
O	-1.00928400	2.23178500	0.60861700
H	2.69475000	0.21098700	-2.17834400
N	-1.76853500	-1.45811900	-0.16787800
H	-1.82934200	-2.39967200	-0.55031600
H	-1.92023100	-1.55920600	0.83336400
C	-2.83323300	-0.62592300	-0.74193500
H	-2.79997200	0.36886200	-0.28726600
H	-2.65881000	-0.50230600	-1.81651500
H	-3.85312800	-1.02150900	-0.61151600

TS1'

0 1

C	-0.03868100	0.94930800	-0.65015800
H	0.68857700	1.04643000	-1.45939100
C	-0.48766000	2.33391000	-0.18661300
H	-1.20687300	2.25373500	0.63433400
H	0.37355100	2.90757500	0.16396900
C	0.54665700	0.12837500	0.51785100
N	-1.14102800	0.08063800	-1.05072900
H	-1.84158000	0.39738200	-1.70889300
C	-1.57419300	-0.67735800	0.01024900
O	-0.63094900	-0.63552900	0.99592400
O	1.23445200	0.73249200	1.47453700
O	-2.60198500	-1.31692800	0.09678200
H	-0.95991700	2.87247600	-1.01433600
N	1.59337700	-0.95382100	0.17166000
H	1.99208300	-0.29904200	1.17331400
H	1.21627000	-1.89151500	0.31820500
C	2.36307000	-0.87463000	-1.08048100
H	1.72573900	-1.04731200	-1.95248700
H	2.83145600	0.10899000	-1.15316600
H	3.14694600	-1.63435100	-1.05109500

INT2'

0 1

N	-1.32160600	-1.02321600	0.33784800
C	-1.29046900	0.27540600	-0.04662300
C	-0.05587600	1.05693000	0.46755100
N	1.14089200	0.66042700	-0.28704200

C	-2.33501700	-1.93927800	-0.15628500
O	-2.15481200	0.79789800	-0.75044900
C	-0.26458500	2.56072800	0.36072500
C	1.84203700	-0.46225200	0.03217900
O	1.56953300	-1.20489800	0.96703900
H	-0.50098900	-1.39647700	0.80481700
H	0.12866900	0.77224600	1.50720500
H	1.27681500	1.10501800	-1.18693300
H	-2.16553400	-2.21342100	-1.20551700
H	-2.32083100	-2.84627000	0.45120000
H	-3.32150100	-1.47479500	-0.08326100
H	0.62431800	3.09179900	0.71223300
H	-1.12061000	2.86430100	0.96841200
H	-0.47433100	2.84941300	-0.67311400
O	2.91898400	-0.73558500	-0.74381400
H	3.09280800	-0.02082600	-1.37816900

TS2’
0 1

N	1.27896300	-0.98708100	0.07660700
C	1.31324300	0.35062100	-0.12029400
C	-0.05657400	1.04856300	-0.30542200
N	-1.13956400	0.44917100	0.52641600
C	2.51094100	-1.75171100	0.19053600
O	2.35773800	1.00029700	-0.15976200
C	0.03796600	2.53717600	0.01036800
C	-1.92302400	-0.73235900	-0.01765800
O	-1.46954400	-1.80361300	-0.36552100
H	0.40675100	-1.49260100	-0.06571100
H	-0.35806400	0.91111100	-1.35092500
H	-0.81618900	0.27223400	1.48134700
H	3.16924800	-1.29627300	0.93496600
H	2.26542900	-2.76820300	0.50336600
H	3.05429800	-1.79298400	-0.76137200
H	-0.90979700	3.03134700	-0.21872400
H	0.83468100	2.99026500	-0.58115700
H	0.27161500	2.69390800	1.06840400
O	-3.10961800	-0.22718300	0.00066700
H	-2.41769400	0.74681600	0.42039100

INT3’
0 1

N	0.71803900	-1.20945700	0.11435400
C	1.57128800	-0.19314100	-0.14356400

C	0.90705500	1.18215100	-0.34805600
N	-0.47630400	1.19044200	0.15849900
C	1.16583200	-2.58308000	0.23691300
O	2.79243200	-0.33874100	-0.23671800
C	1.79217300	2.29462400	0.22257100
C	-2.95958500	-0.16284600	-0.06386600
O	-2.52629200	-1.24230500	-0.20173400
H	-0.27001500	-0.99478400	0.09412100
H	0.84421000	1.30824500	-1.43745700
H	-0.46075300	1.28256200	1.17410600
H	2.07891800	-2.62292800	0.83647500
H	0.38724600	-3.17080600	0.72799800
H	1.38298900	-3.03349200	-0.74028900
H	1.36933200	3.27548800	-0.01781200
H	2.80342300	2.23357200	-0.18611200
H	1.86081200	2.20753000	1.31344200
O	-3.46534100	0.88138100	0.07451900
H	-0.95527200	2.01879600	-0.18697100

INT4’

0 1

C	0.16437301	2.46482717	-0.40527497
H	0.77283901	2.05912017	0.41010703
C	0.69355401	3.82978317	-0.84136397
H	0.08781801	4.24329817	-1.65271597
H	0.67338101	4.51996817	0.00650403
C	-1.27821599	2.52209417	0.07435403
N	0.04691701	1.49825017	-1.49028097
H	0.83855101	0.94946917	-1.80563597
C	-1.18123799	0.91875317	-1.51754997
O	-2.03068499	1.65798817	-0.68392797
O	-1.75539599	3.21113017	0.93465703
O	-1.57531399	-0.03362083	-2.14613997
H	1.72672701	3.73533217	-1.18731697
N	-0.47339399	-0.33879883	1.02503203
H	-0.11414399	-0.07828083	1.94239303
H	-1.35657499	-0.82091183	1.17488903
C	0.48169901	-1.25810983	0.39941903
H	0.10828201	-1.49178083	-0.60443197
C	1.82517301	-0.51409583	0.28487503
H	2.52773201	-1.12555583	-0.24183897
H	1.68085601	0.40278917	-0.24747297
H	2.20038401	-0.30257583	1.26435303
C	0.71891601	-2.56364283	1.18002303

O	0.18973701	-2.83641583	2.28868703
N	1.68597401	-3.49760083	0.42894203
C	2.37114101	-4.79176383	0.30015503
H	1.66314301	-5.54395983	0.02115403
H	3.13015201	-4.72033383	-0.45064397
H	2.81885401	-5.05300983	1.23621303
H	2.62886901	-3.25088883	0.65273103

TS4’

0 1

C	-1.83129600	0.75442100	-0.77122900
H	-1.06027400	1.21548100	-1.39322500
C	-3.05540500	1.66641100	-0.70908700
H	-3.83293800	1.22857900	-0.07583700
H	-2.77748400	2.63713200	-0.29186200
C	-1.28905200	0.44737400	0.64075900
N	-2.12727600	-0.59554200	-1.23776900
H	-2.65648700	-0.74739000	-2.08709100
C	-2.39567600	-1.42845300	-0.17710900
O	-1.96635500	-0.83146600	0.97149400
O	-1.36437200	1.36715300	1.59199300
O	-2.90608000	-2.52887400	-0.20848900
H	-3.46378500	1.81557900	-1.71366000
N	0.21164300	0.13004300	0.78564300
H	-0.12373600	0.93643400	1.69509200
H	0.38413200	-0.84848700	1.03933500
C	1.20587100	0.53119400	-0.23018500
H	0.83475700	0.24319800	-1.22247800
C	1.45362800	2.03948100	-0.17803000
H	0.50478400	2.58003000	-0.21512500
H	1.96485500	2.31406900	0.74945100
H	2.05547000	2.37177800	-1.02836700
C	2.45080800	-0.34967400	0.01720700
O	2.34636100	-1.42546000	0.61302000
C	4.83847800	-0.68616300	-0.36948600
H	5.66145900	-0.11448900	-0.80006100
H	5.06105400	-0.90233600	0.67967900
H	4.74565600	-1.63830400	-0.90212400
N	3.61667000	0.09764400	-0.48391800
H	3.65470800	0.98533600	-0.96260300

INT5’

0 1

N	-0.00114600	0.72969700	1.15798100

C	0.64183200	1.37423900	0.14244400
C	2.12054800	0.96602600	-0.02827100
N	2.16161100	-0.36182000	-0.65992900
C	-1.45080100	0.63375100	1.12641200
O	0.08508200	2.17167500	-0.60874100
C	2.89422500	1.97137200	-0.86810500
C	1.98896900	-1.49430500	0.08630000
O	1.93666800	-1.51408100	1.30912700
H	0.49385500	-0.06435800	1.55797000
H	2.57977300	0.85239100	0.95700400
H	1.87993700	-0.38467800	-1.63343600
H	-1.86124700	1.64712000	1.07759300
H	3.92540100	1.63487600	-1.00530200
H	2.90383800	2.94627300	-0.37338900
H	2.42328400	2.10043500	-1.84672000
O	1.94045100	-2.65580300	-0.60265300
H	2.03211000	-2.50769800	-1.55773500
C	-1.95576400	-0.07726000	2.38538500
H	-3.04760900	-0.13209000	2.38119800
H	-1.56172600	-1.09837800	2.43172800
H	-1.63470800	0.46102200	3.28205000
C	-3.72522600	-0.53026700	-1.72125400
H	-3.30312500	-0.14026500	-2.65581100
H	-3.52524200	-1.60367900	-1.67973600
H	-4.80452300	-0.36657100	-1.72594600
C	-1.89262400	-0.14556900	-0.13509200
O	-1.16790500	-0.98460300	-0.67033100
N	-3.15081700	0.12222100	-0.55660900
H	-3.64887600	0.89949500	-0.14568900

TS5’

0 1

N	0.14451200	0.39407700	-0.05773100
C	-0.83593100	1.29449200	-0.29747300
C	-2.24261300	0.71090000	-0.56885600
N	-2.62707000	-0.30201400	0.45595400
C	1.49821400	0.83912200	0.23860900
O	-0.64663000	2.51209800	-0.30706800
C	-3.30007900	1.80597300	-0.61363800
C	-2.32635000	-1.76400500	0.16641100
O	-1.24905100	-2.22810200	-0.13468600
H	-0.02114900	-0.61251500	-0.10673400
H	-2.21044300	0.18330200	-1.52869700
H	-2.29667600	-0.03638100	1.38747100

H	1.71003800	1.71623300	-0.38178700
H	-4.27195200	1.37437900	-0.86748700
H	-3.03276500	2.55330200	-1.36267600
H	-3.37673800	2.31116000	0.35358500
O	-3.51487800	-2.25199900	0.29769100
H	-3.72886100	-1.01086100	0.47644900
C	1.64785100	1.23285000	1.71860500
H	2.66002000	1.59117500	1.92873200
H	0.93885800	2.03075800	1.95417700
H	1.44148800	0.37183100	2.36247600
C	4.82014900	-0.85029200	-0.56485700
H	5.78302500	-0.39260500	-0.33026000
H	4.69154300	-1.74883800	0.04302200
H	4.81662800	-1.14819400	-1.62046400
N	3.75923000	0.09799800	-0.26762100
H	3.97681400	1.08472700	-0.27169300
C	2.47258400	-0.29153200	-0.11559800
O	2.10812400	-1.46486600	-0.19712800

INT6’

0 1

N	0.36286400	-0.65447700	-0.17373200
C	1.55117500	-1.27797100	-0.30731400
C	2.72647400	-0.34700200	-0.66644200
N	2.41332500	1.04848200	-0.31668600
C	-0.87677500	-1.33559500	0.13020700
O	1.69648400	-2.49812100	-0.18850200
C	4.03097400	-0.87552700	-0.06237900
C	0.17449300	2.89076500	0.42066500
O	0.14963400	3.41340700	-0.62397300
H	0.30755000	0.34732100	-0.30389600
H	2.80315100	-0.39049700	-1.76147100
H	2.54231800	1.17655100	0.68609000
H	-0.95144300	-2.23799100	-0.48813600
H	4.87720900	-0.26936000	-0.40151500
H	4.20077900	-1.91550100	-0.35114600
H	3.99157000	-0.82992300	1.03236500
O	0.20403700	2.42321000	1.49289100
H	3.08191300	1.66798000	-0.76857700
C	-0.95387200	-1.74133800	1.61344500
H	-1.88298300	-2.27775000	1.83014900
H	-0.10946800	-2.39391000	1.84759300
H	-0.90310000	-0.85253400	2.25018200
C	-4.45753200	-0.21013100	-0.59863200

H	-5.21033200	-0.43080500	0.16481300
H	-4.22628800	0.85500000	-0.57557800
H	-4.86821300	-0.46272900	-1.58240500
N	-3.23454000	-0.94999600	-0.33736200
H	-3.29647900	-1.95540800	-0.24998400
C	-2.02019000	-0.36932300	-0.22362900
O	-1.82468100	0.84378100	-0.35160900

B(TMP)MCl

N	1.08594200	0.00079600	0.62505400
C	0.68006400	0.00112900	1.95242900
C	-0.68108900	-0.00145100	1.95190800
N	-1.08598700	-0.00345000	0.62424500
C	0.00027400	-0.00199000	-0.15813200
C	2.45645300	0.00435300	0.16238000
C	-2.45624900	-0.00839100	0.16080600
C	3.10585400	-1.22711500	0.00134000
C	4.43621400	-1.19646100	-0.42886300
C	5.09963900	0.00820900	-0.68797600
C	4.40688200	1.21200200	-0.50550700
C	3.07669500	1.23929700	-0.07876800
C	-3.07406300	-1.24129800	-0.08342300
C	-4.40548400	-1.21423100	-0.51473900
C	-5.09758000	-0.01271100	-0.69541200
C	-4.43365900	1.19403000	-0.43423700
C	-3.10690600	1.22501200	-0.00124300
C	2.39762800	-2.52897200	0.27822700
C	2.33648900	2.54016600	0.10316000
C	6.52671700	0.01687500	-1.17509200
C	-2.39749600	2.52603500	0.27649300
C	-2.33490900	-2.54327700	0.09483300
C	-6.53210200	-0.00095400	-1.16029500
H	1.39636000	0.00347100	2.75827100
H	-1.39799400	-0.00269300	2.75721000
H	0.00067200	-0.00299800	-1.23635500
H	4.96502200	-2.13652500	-0.56077500
H	4.91511600	2.15366900	-0.69618900
H	-4.91079100	-2.15623000	-0.70970000
H	-4.96339000	2.13358800	-0.56886100
H	1.54175200	-2.66600600	-0.39235700
H	3.07372200	-3.37509900	0.13866200
H	2.01227400	-2.56649900	1.30296100
H	3.02282300	3.38752300	0.04208400

H	1.57321500	2.67192800	-0.67280900
H	1.82451500	2.58399600	1.07001100
H	7.10224700	0.82067600	-0.70521800
H	7.02790200	-0.93276900	-0.96856900
H	6.56426400	0.18340300	-2.25877600
H	-3.07211300	3.37299600	0.13480900
H	-2.01456800	2.56356300	1.30214100
H	-1.53975300	2.66143800	-0.39204300
H	-1.81051900	-2.58444600	1.05506900
H	-3.02420700	-3.38906600	0.04618300
H	-1.58243900	-2.68058700	-0.69074900
H	-6.91997500	-1.01369100	-1.29714100
H	-7.17392900	0.51516100	-0.43732700
H	-6.63011500	0.53221900	-2.11294800

1. Wu, Y.; Chen, K.; Wu, X.; Liu, L.; Zhang, W.; Ding, Y.; Liu, S.; Zhou, M.; Shao, N.; Ji, Z.; Chen, J.; Zhu, M.; Liu, R., Superfast and water-insensitive polymerization on α-amino acid N-carboxyanhydrides to prepare polypeptides using tetraalkylammonium carboxylate as the initiator. *Angew. Chem. Int. Ed.* **2021,** *60* (50), 26063-26071.
2. Chan, N. J.; Lentz, S.; Gurr, P. A.; Tan, S.; Scheibel, T.; Qiao, G. G., Crosslinked polypeptide films via RAFT-mediated continuous assembly of polymers. *Angew. Chem. Int. Ed.* **2022,** *61* (9), e202112842.
3. Wu, Y.; Zhang, D.; Ma, P.; Zhou, R.; Hua, L.; Liu, R., Lithium hexamethyldisilazide initiated superfast ring opening polymerization of alpha-amino acid N-carboxyanhydrides. *Nat. Commun.* **2018,** *9*, 5297.
4. Otake, Y.; Nakamura, H.; Fuse, S., Rapid and mild synthesis of amino acid N-carboxy anhydrides: Basic-to-acidic flash switching in a microflow reactor. *Angew. Chem. Int. Ed.* **2018,** *57* (35), 11389-11393.
5. Semple, J. E.; Sullivan, B.; Sill, K. N., Large-scale synthesis of α-amino acid-N-carboxyanhydrides. *Synth. Commun.* **2017,** *47* (1), 53-61.
6. D. Bull, S.; G. Davies, S.; M. Parkin, R.; Sánchez-Sancho, F., The biosynthetic origin of diketopiperazines derived from D-proline. *J. Chem. Soc., Perkin Trans. 1* **1998,** 15, 2313-2320.
7. Wu, L.; Song, J.; Zhang, B.; Zhou, B.; Zhou, H.; Fan, H.; Yang, Y.; Han, B., Very efficient conversion of glucose to 5-hydroxymethylfurfural in DBU-based ionic liquids with benzenesulfonate anion. *Green Chem.* **2014,** *16* (8), 3935-3941.
8. Frisch, M. J.; Trucks, G. W.; Schlegel, H. B.; Scuseria, G. E.; Robb, M. A.; Cheeseman, J. R.; Scalmani, G.; Barone, V.; Petersson, G. A.; Nakatsuji, H.; Caricato, M.; Li, X.; Hratchian, H. P.; Izmaylov, A. F.; Bloino, J.; Zheng, G.; Sonnenberg, J. L.; Hada, M.; Ehara, M.; Toyota, K.; Fukuda, R.; Hasegawa, J.; Ishida, M.; Nakajima, T.; Honda, Y.; Kitao, O.; Nakai, H.; Vreven, T.; Montgomery Jr., J. A.; Peralta, J. E.; Ogliaro, F.; Bearpark, M. J.; Heyd, J. J.; Brothers, E. N.; Kudin, K. N.; Staroverov, V. N.; Keith, T. A.; Kobayashi, R.; Normand, J.; Raghavachari, K.; Rendell, A. P.; Burant, J. C.; Iyengar, S. S.; Tomasi, J.; Cossi, M.; Rega, N.; Millam, J. M.; Klene, M.; Knox, J. E.; Cross, J. B.; Bakken, V.; Adamo, C.; Jaranillo, J.; Gomperts, R.; Stratmann, R. E.; Yazyev, O.; Austin, A. J.; Cammi, R.; Pomelli, C.; Ochterski, J. W.; Martin, R. L.; Morokuma, K.; Zakrzewski, V. G.; Voth, G. A.; Salvador, P.; Dannenberg, J. J.; Dapprich, S.; Daniels, A. D.; Farkas, Ö.; Foresman, J. B.; Ortiz, J. V.; Cioslowski, J.; Fox, D. J. *Gaussian 09 Rev. E.01*, Wallingford, CT, **2009**.
9. Miertus, S.; Tomasi, J. J. C. p., Approximate evaluations of the electrostatic free energy and internal energy changes in solution processes. *Chem. Phys.* **1982,** *65* (2), 239-245.
10. Grimme, S.; Antony, J.; Ehrlich, S.; Krieg, H., A consistent and accurate ab initio parametrization of density functional dispersion correction (DFT-D) for the 94 elements H-Pu. *J. Chem. Phys.* **2010,** *132* (15), 154104.
11. Lu, T.; Chen, Q., Interaction Region Indicator: A Simple Real Space Function Clearly Revealing Both Chemical Bonds and Weak Interactions. *Chem. Methods* **2021,** *1* (5), 231-239.
12. Johnson, E. R.; Keinan, S.; Mori-Sánchez, P.; Contreras-García, J.; Cohen, A. J.; Yang, W., Revealing Noncovalent Interactions. *J. Am. Chem. Soc.* **2010,** *132* (18), 6498-6506.
13. Lu, T.; Chen, F., Multiwfn: A multifunctional wavefunction analyzer. *J. Comput. Chem.* **2012,** *33* (5), 580-592.
14. Humphrey, W.; Dalke, A.; Schulten, K., VMD: Visual molecular dynamics. *J. Mol. Graph.*

1996, *14* (1), 33-38.
